# Supplementary figures and images for: PITAR, a DNA damage-inducible cancer/testis long noncoding RNA, inactivates p53 by binding and stabilizing TRIM28 mRNA
Source: eLife. 2024 Sep 20;12:RP88256. doi: 10.7554/eLife.88256 (PMC11415074; doi:10.7554/eLife.88256)

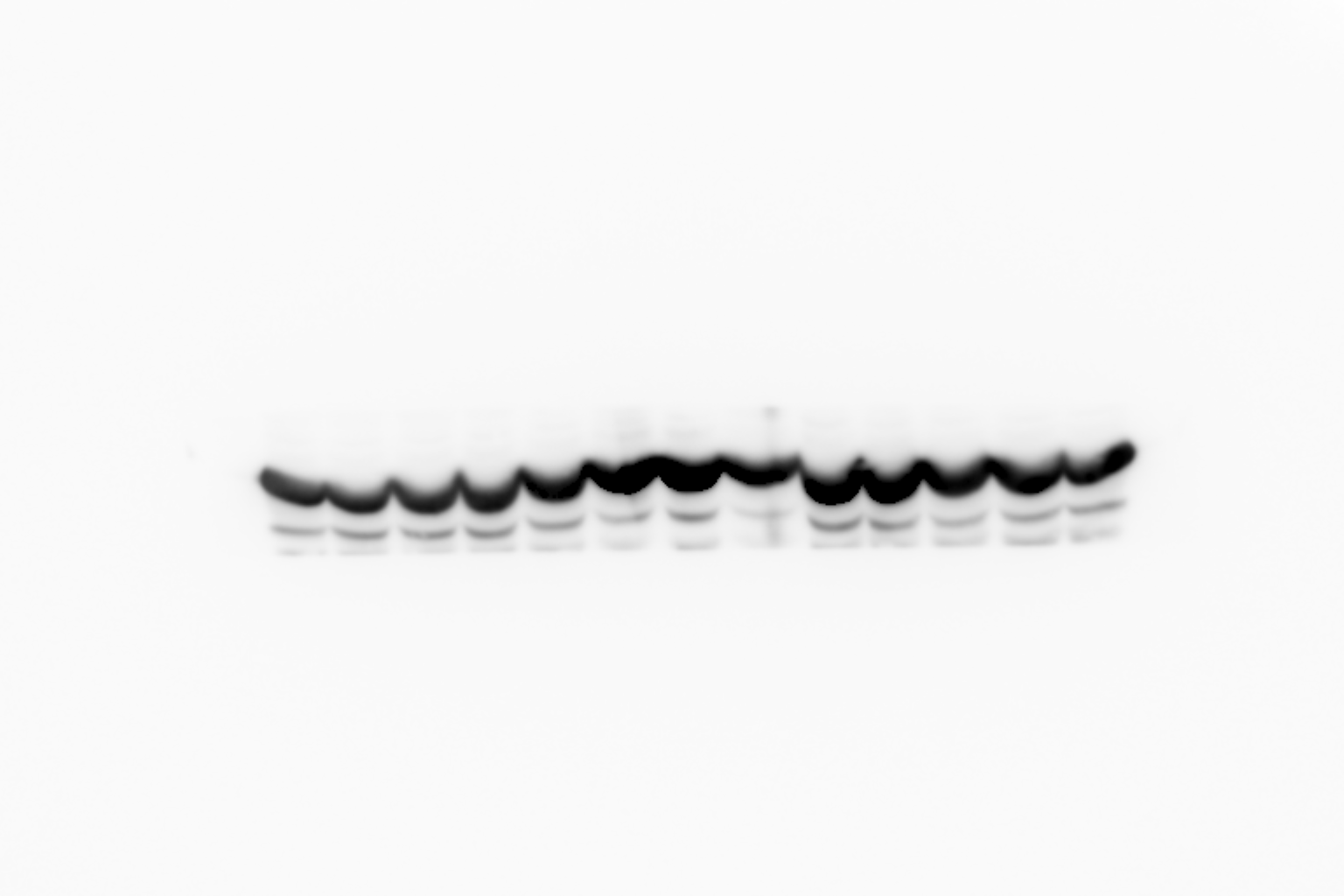

Supplement: Figure 4—source data 1. [file elife-88256-fig4-data1.zip › Figure 4-source data 1. Raw unedited blots for (Figure 4)/GAPDH.tif]

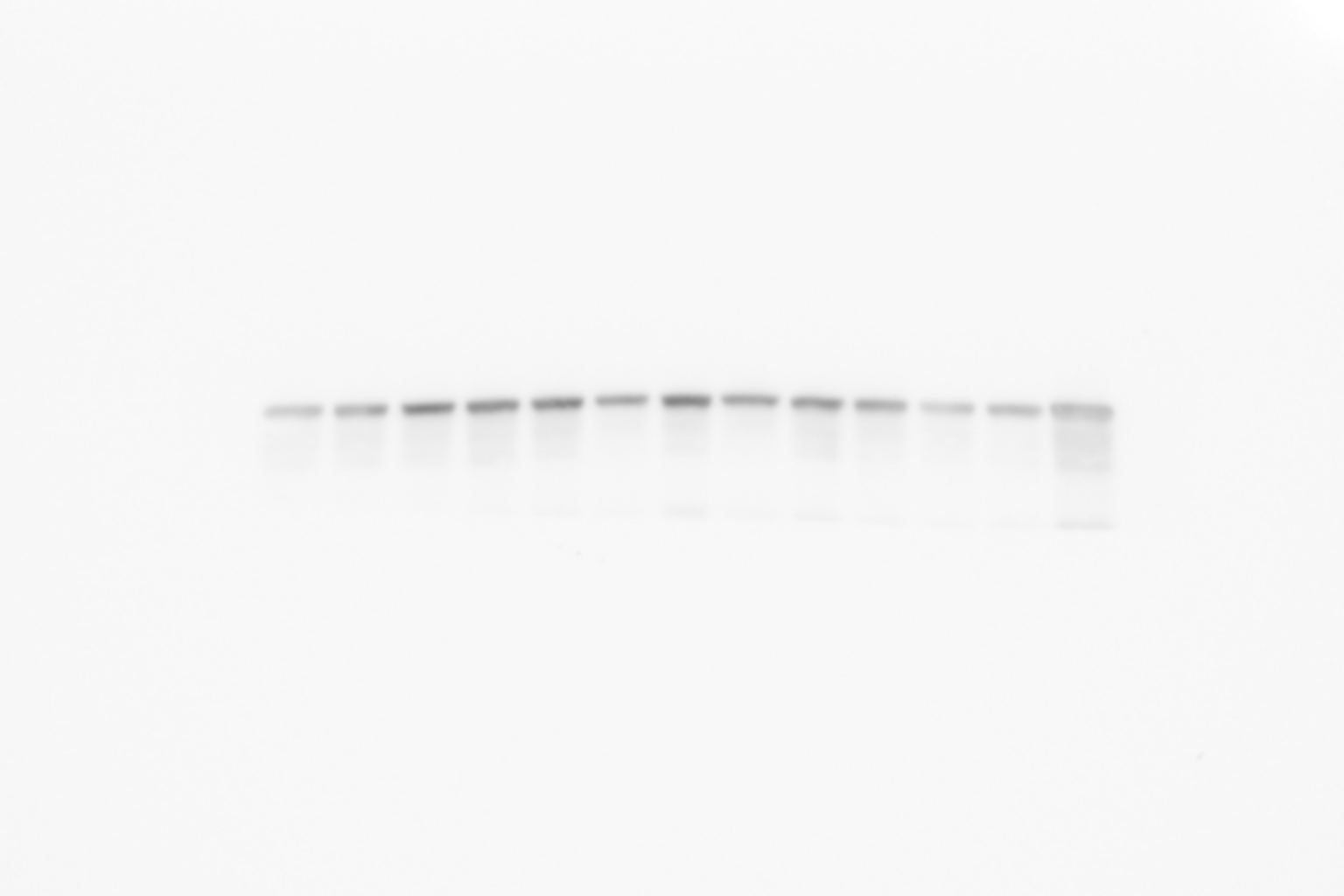

Supplement: Figure 4—source data 1. [file elife-88256-fig4-data1.zip › Figure 4-source data 1. Raw unedited blots for (Figure 4)/TRIM28.tif]

**Figure 4J:**

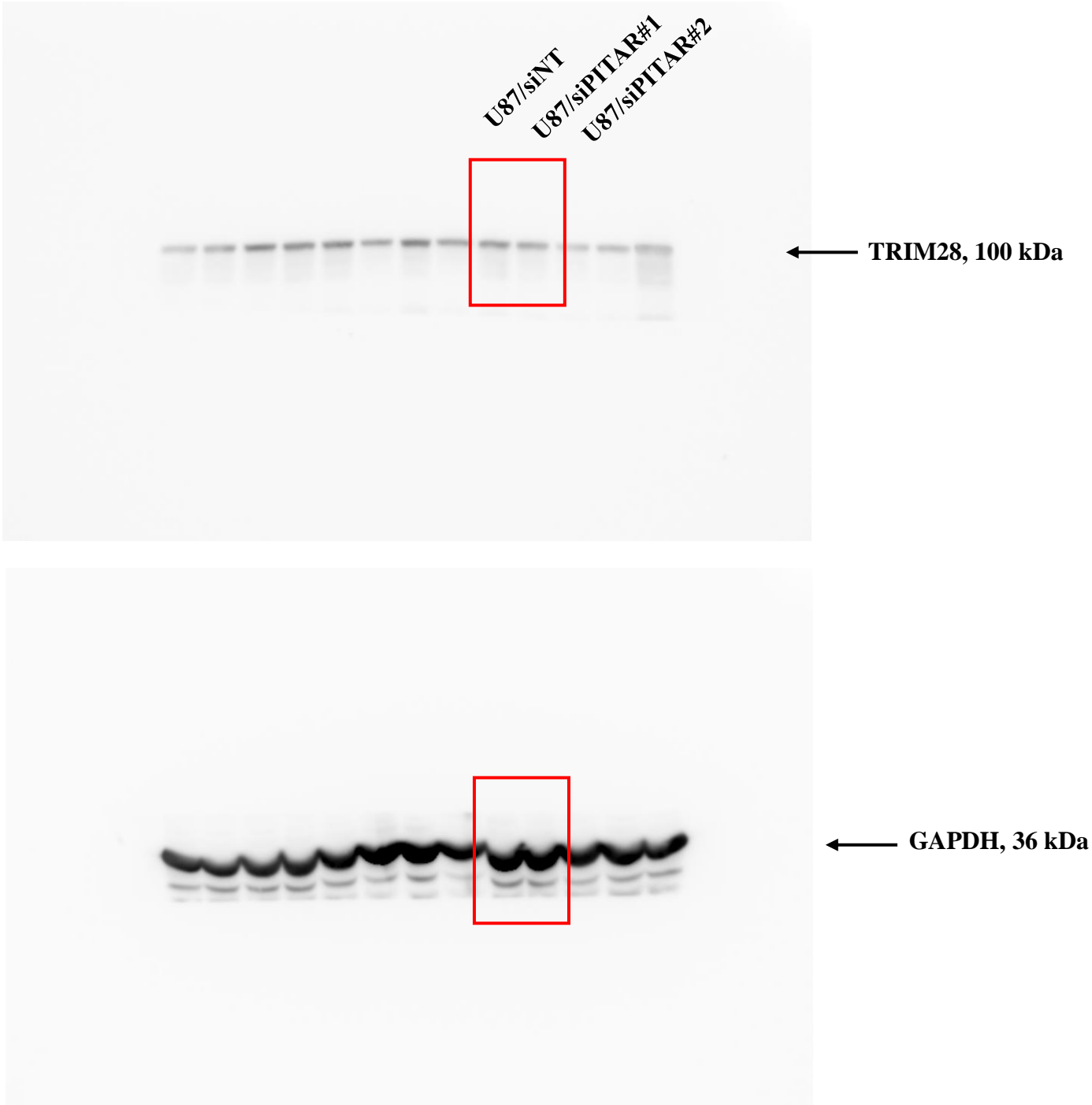

Supplement: Figure 4—source data 2. [file elife-88256-fig4-data2.pdf]

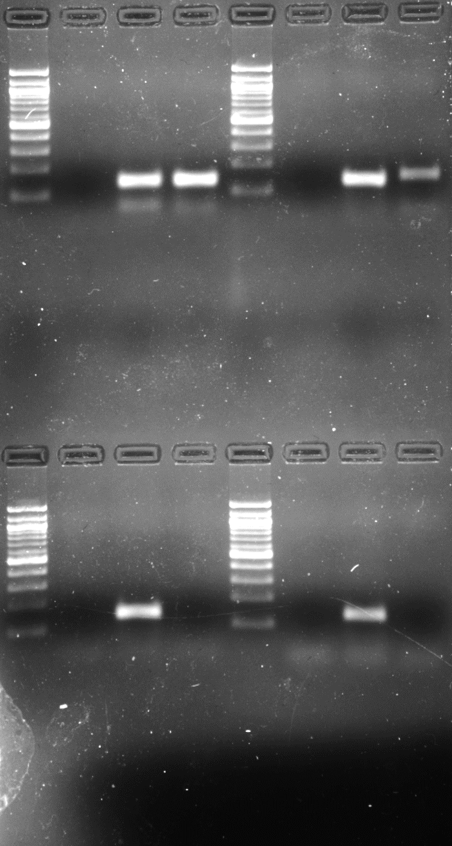

Supplement: Figure 4—figure supplement 2—source data 1. [file elife-88256-fig4-figsupp2-data1.zip › Figure 4-figure supplement 2-source data 1. Raw unedited gels for (Figure 4-figure supplement 2)/ChIRP_PD.tif]

Figure 4-figure supplement 2B

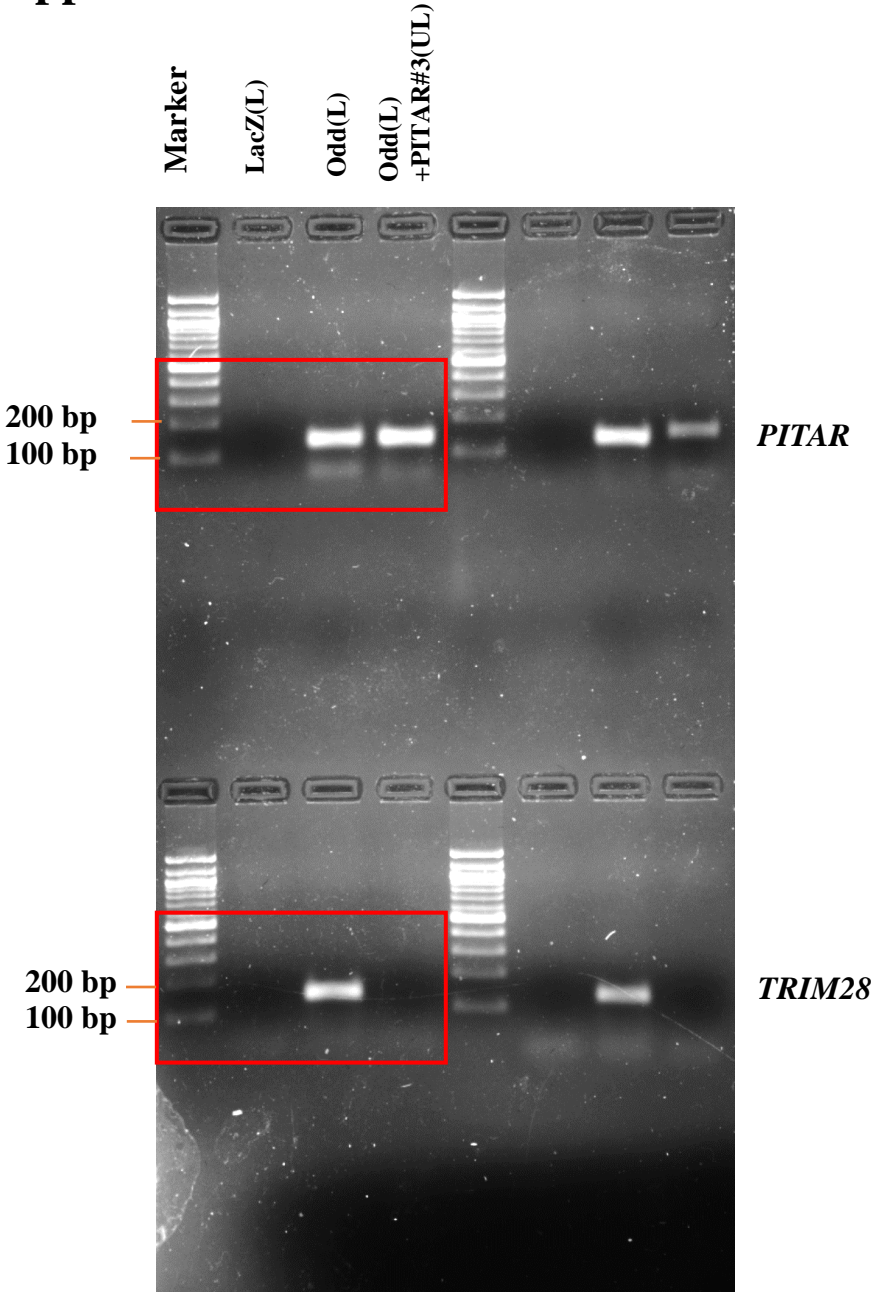

Supplement: Figure 4—figure supplement 2—source data 2. [file elife-88256-fig4-figsupp2-data2.pdf]

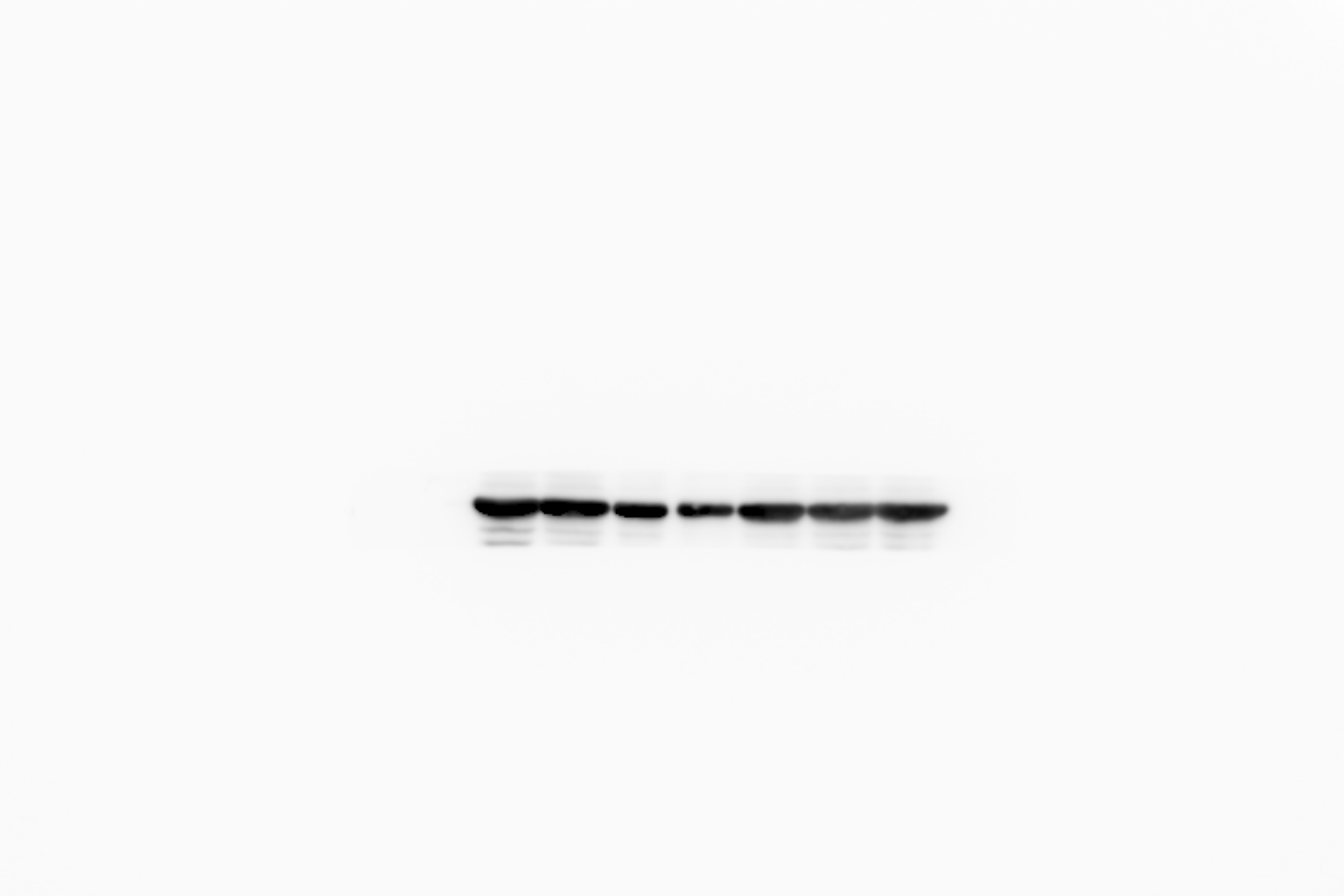

Supplement: Figure 5—source data 1. [file elife-88256-fig5-data1.zip › Figure 5-source data 1. Raw unedited blots for (Figure 5)/Figure 5B/GAPDH.tif]

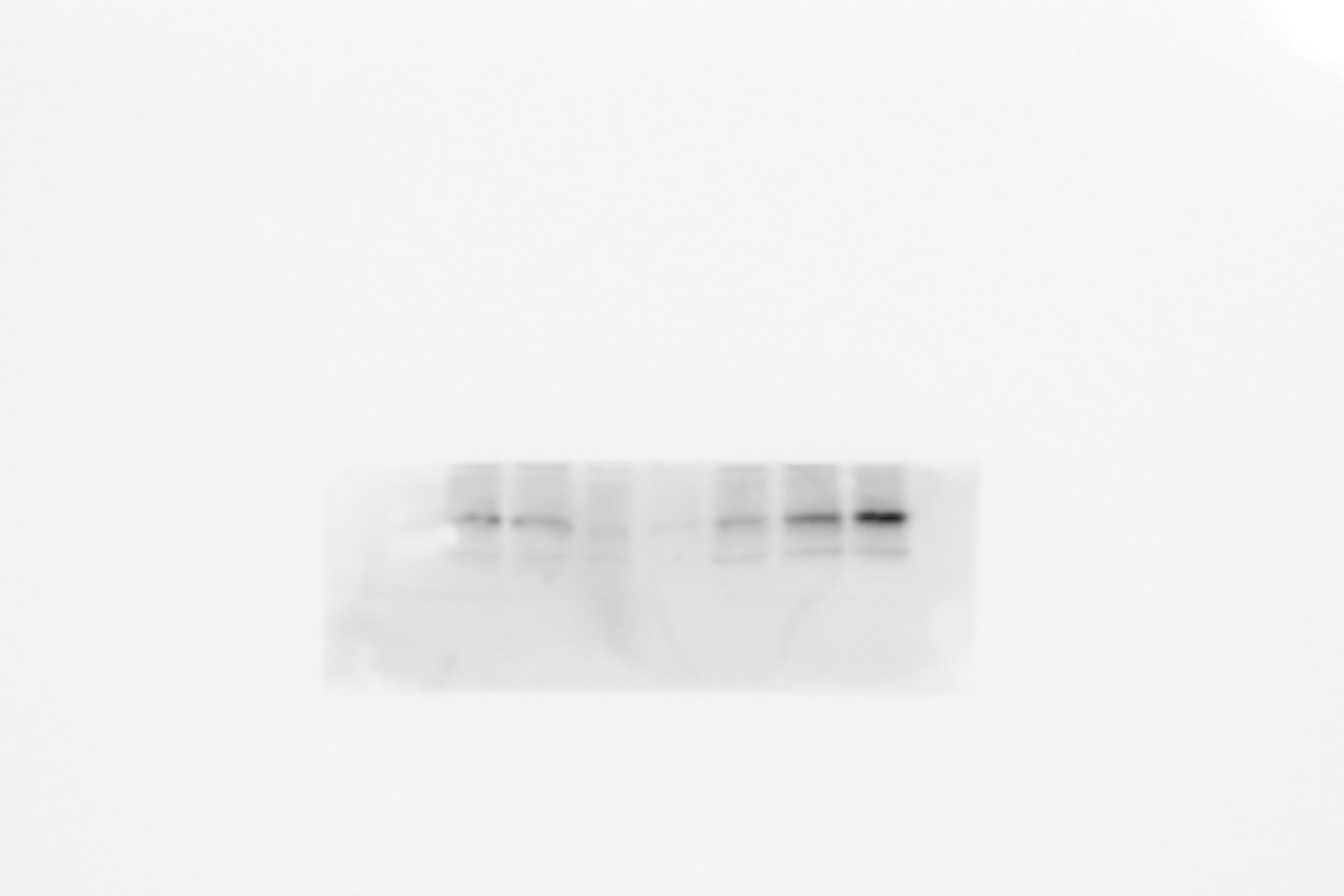

Supplement: Figure 5—source data 1. [file elife-88256-fig5-data1.zip › Figure 5-source data 1. Raw unedited blots for (Figure 5)/Figure 5B/p21.tif]

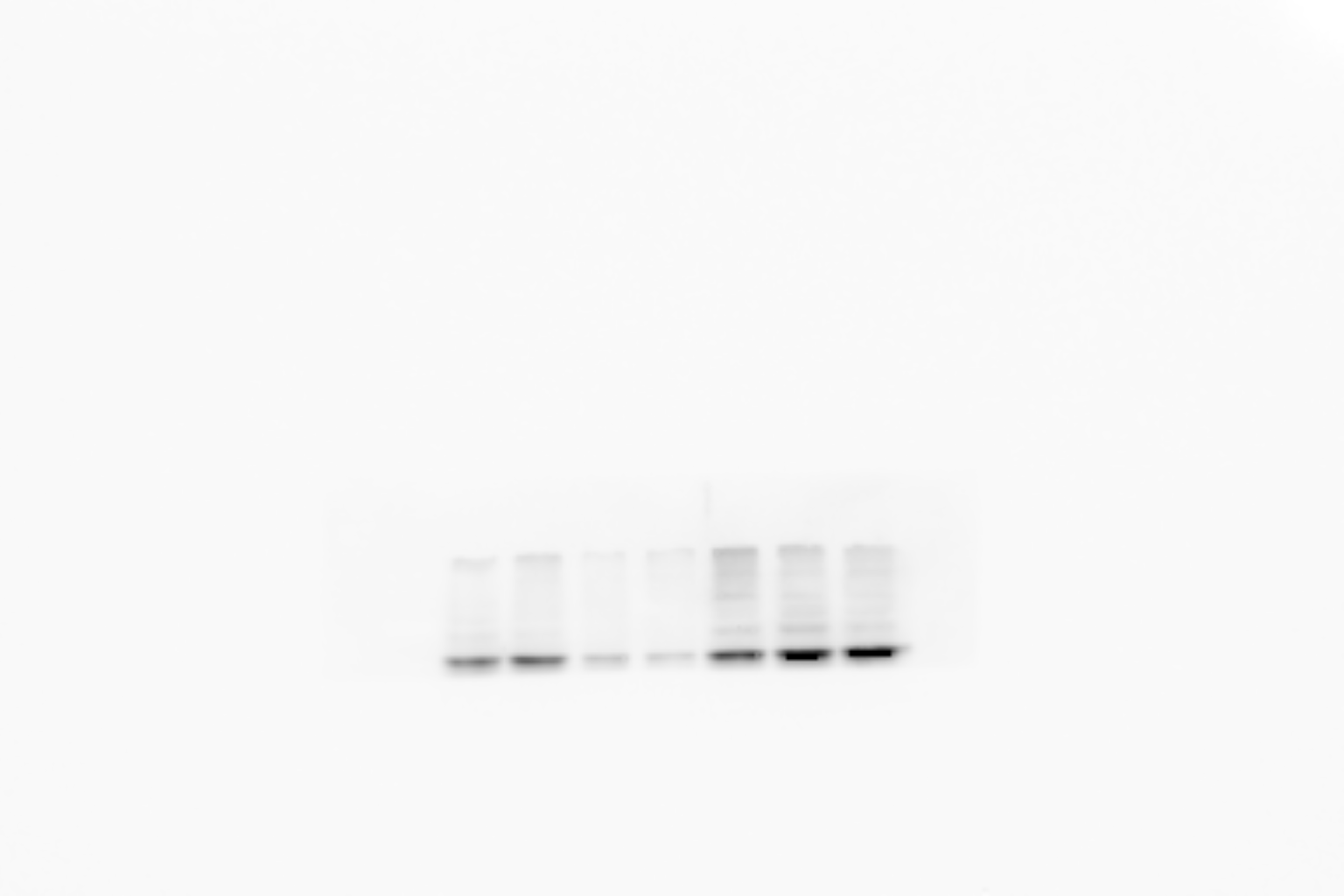

Supplement: Figure 5—source data 1. [file elife-88256-fig5-data1.zip › Figure 5-source data 1. Raw unedited blots for (Figure 5)/Figure 5B/p53.tif]

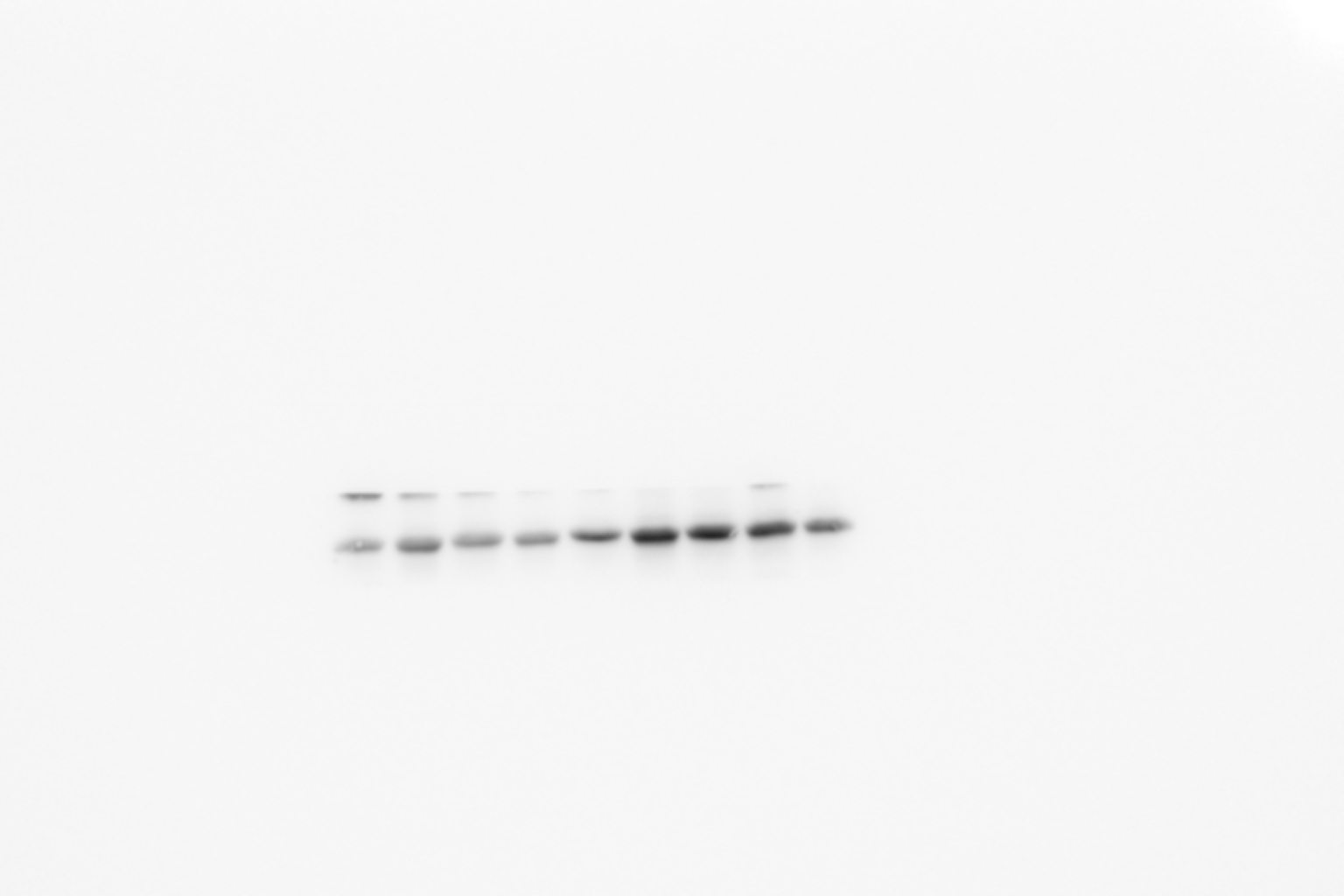

Supplement: Figure 5—source data 1. [file elife-88256-fig5-data1.zip › Figure 5-source data 1. Raw unedited blots for (Figure 5)/Figure 5D/GAPDH.tif]

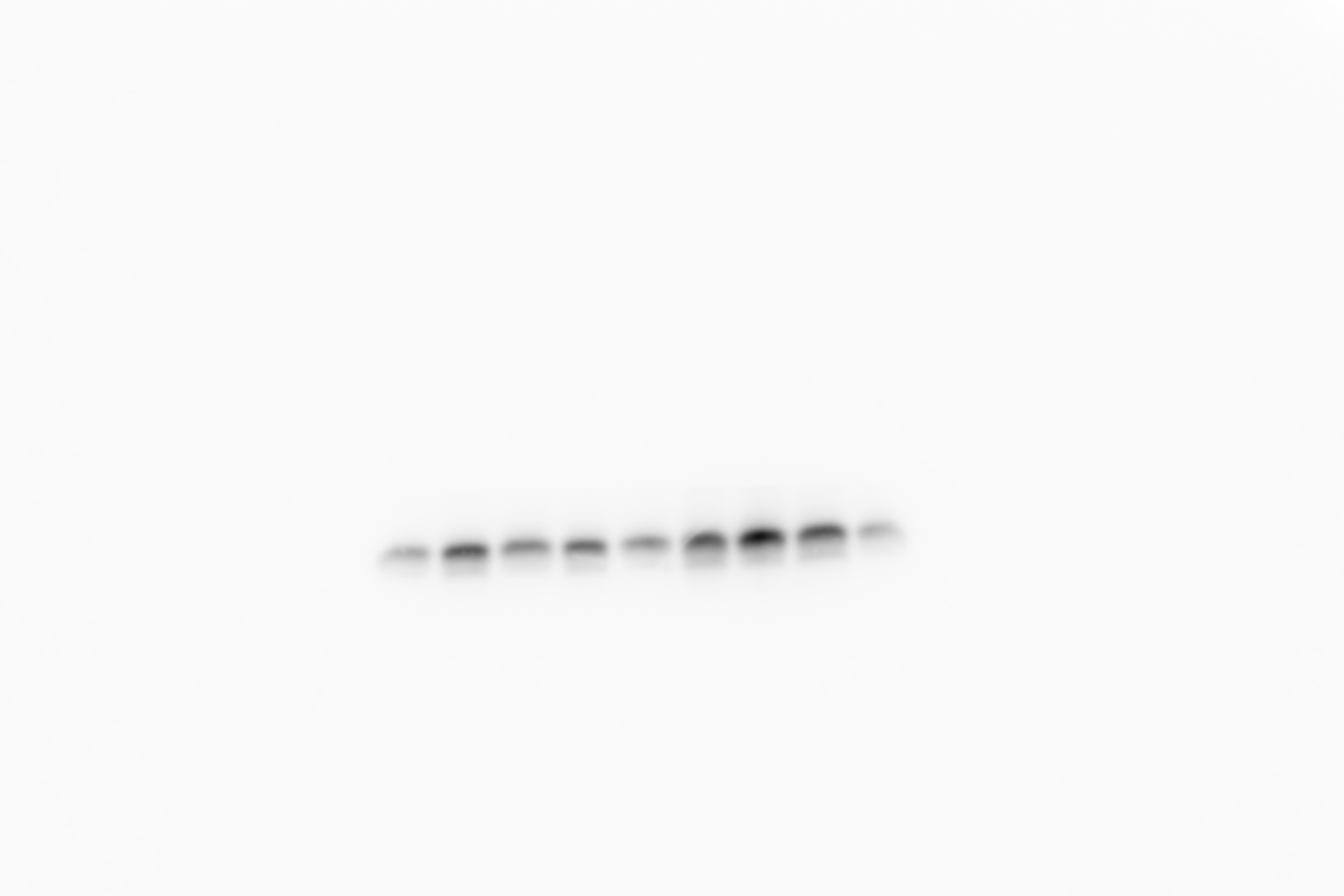

Supplement: Figure 5—source data 1. [file elife-88256-fig5-data1.zip › Figure 5-source data 1. Raw unedited blots for (Figure 5)/Figure 5D/p21.tif]

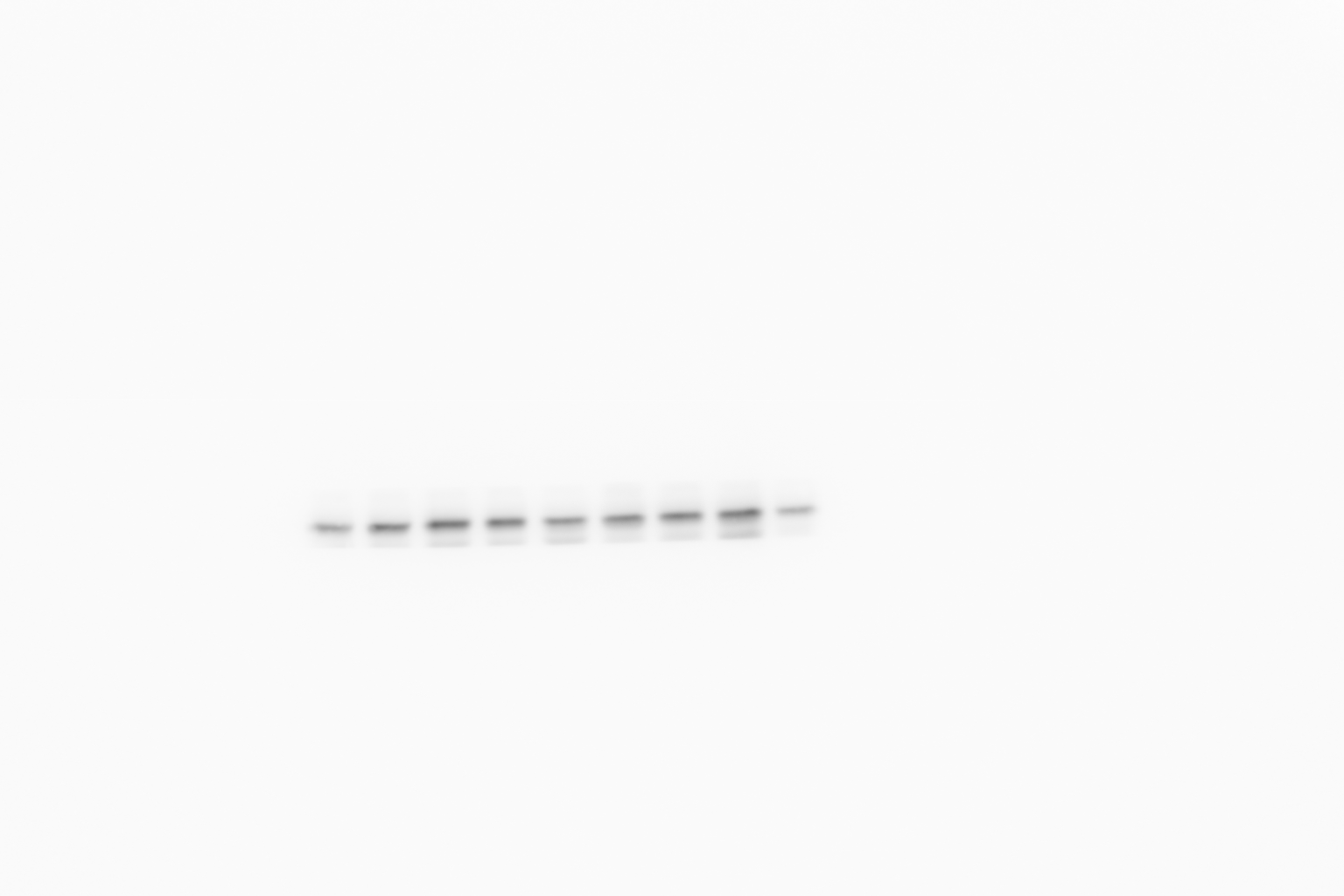

Supplement: Figure 5—source data 1. [file elife-88256-fig5-data1.zip › Figure 5-source data 1. Raw unedited blots for (Figure 5)/Figure 5D/p53.tif]

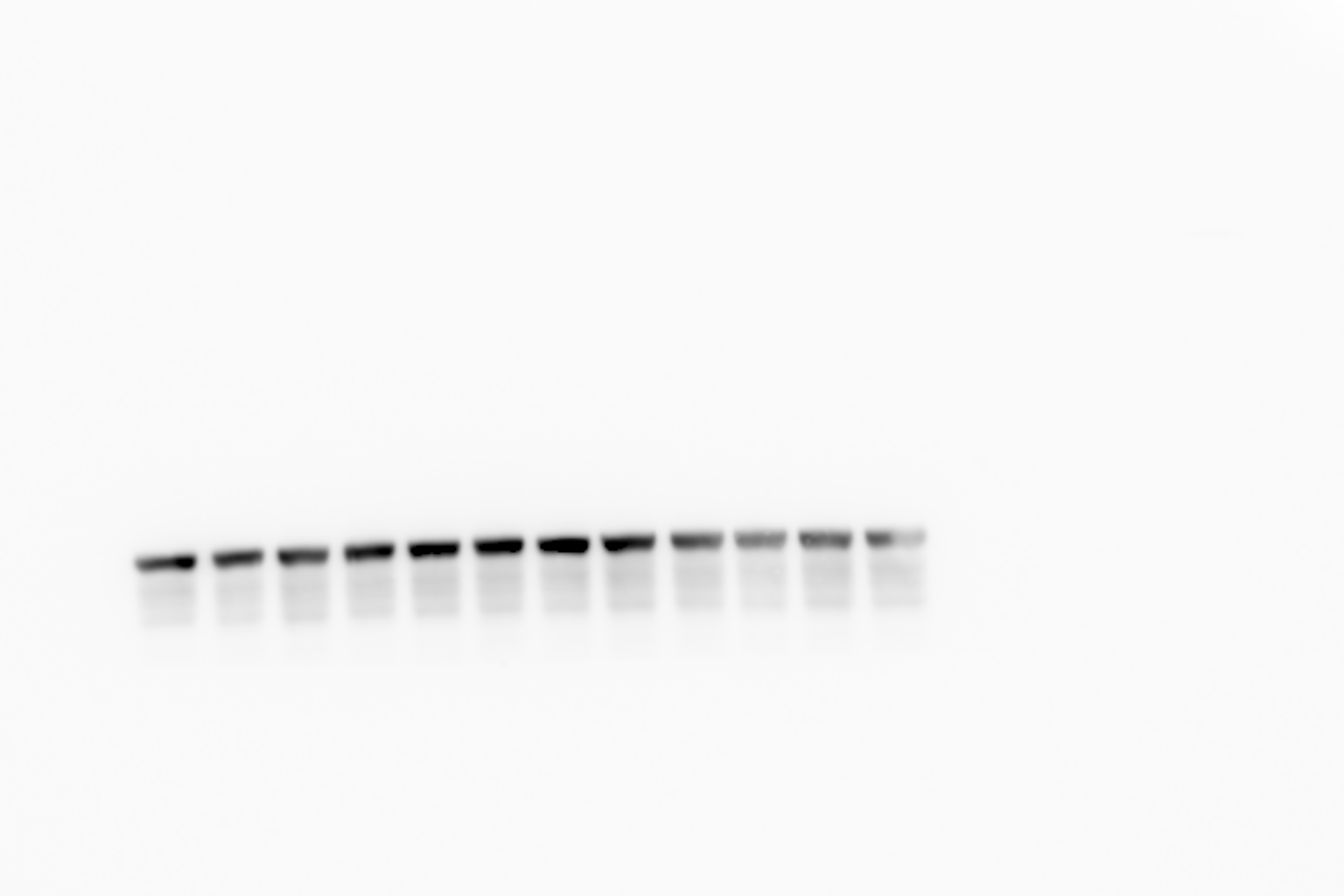

Supplement: Figure 5—source data 1. [file elife-88256-fig5-data1.zip › Figure 5-source data 1. Raw unedited blots for (Figure 5)/Figure 5D/TRIM28.tif]

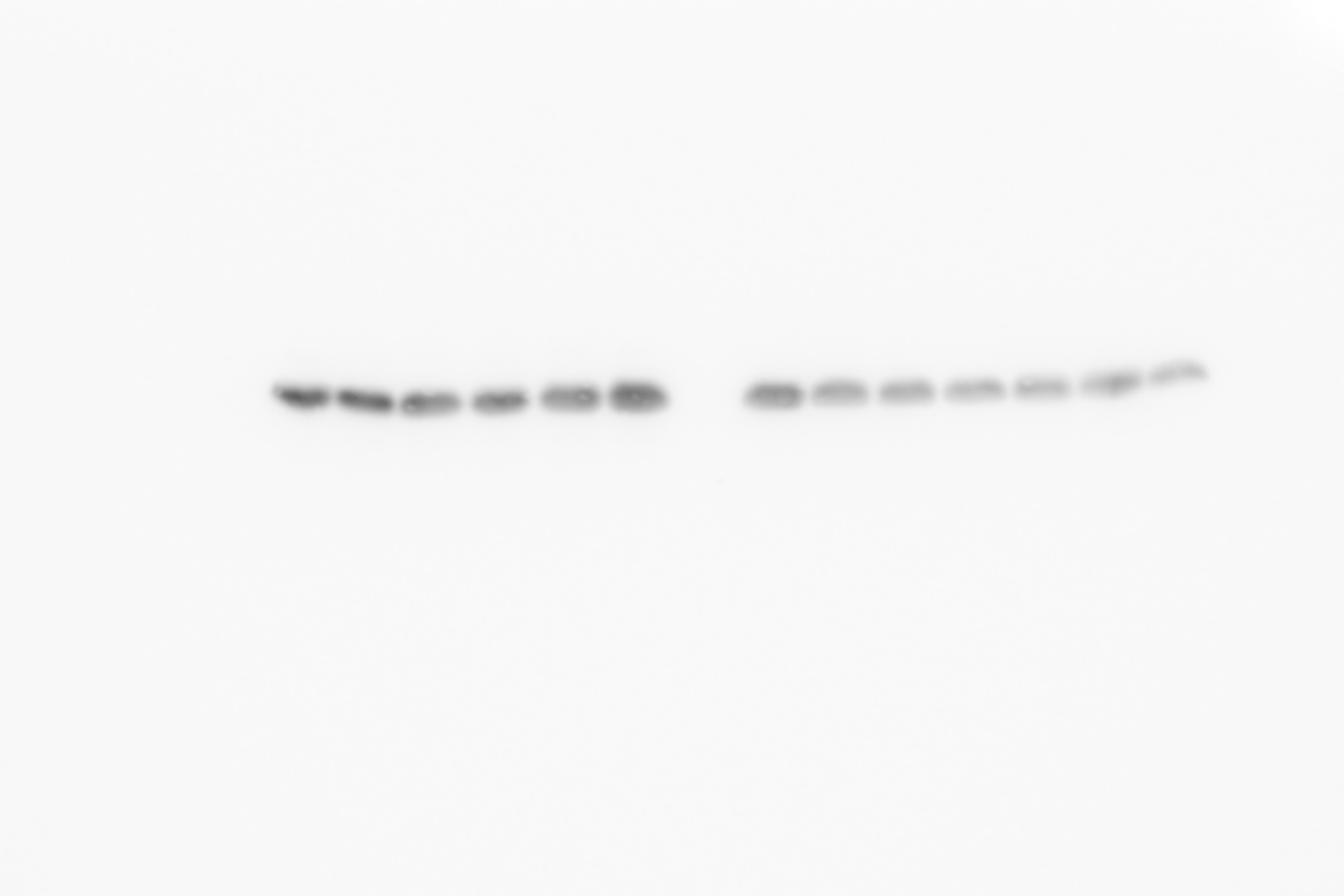

Supplement: Figure 5—source data 1. [file elife-88256-fig5-data1.zip › Figure 5-source data 1. Raw unedited blots for (Figure 5)/Figure 5E/GAPDH.tif]

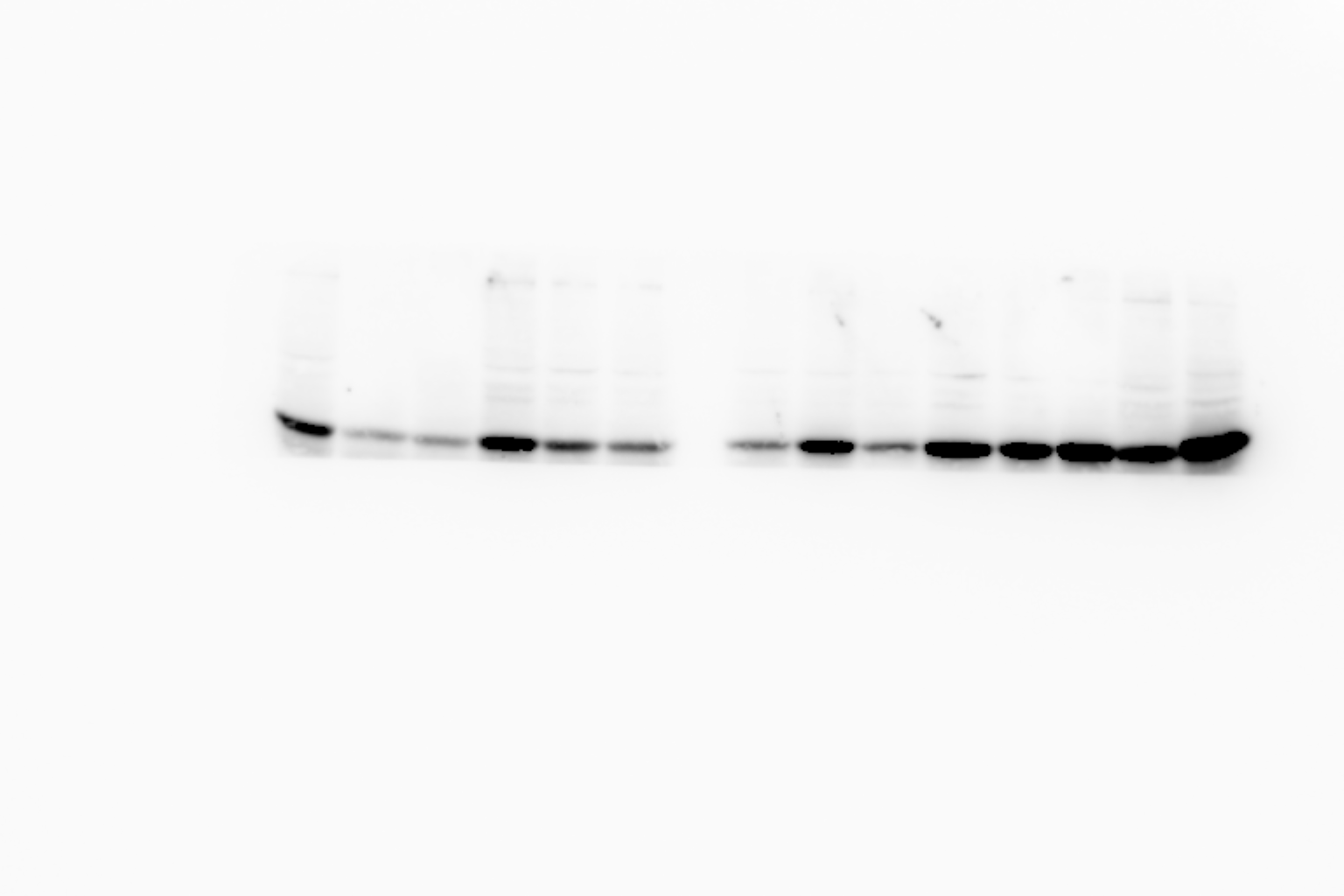

Supplement: Figure 5—source data 1. [file elife-88256-fig5-data1.zip › Figure 5-source data 1. Raw unedited blots for (Figure 5)/Figure 5E/p53.tif]

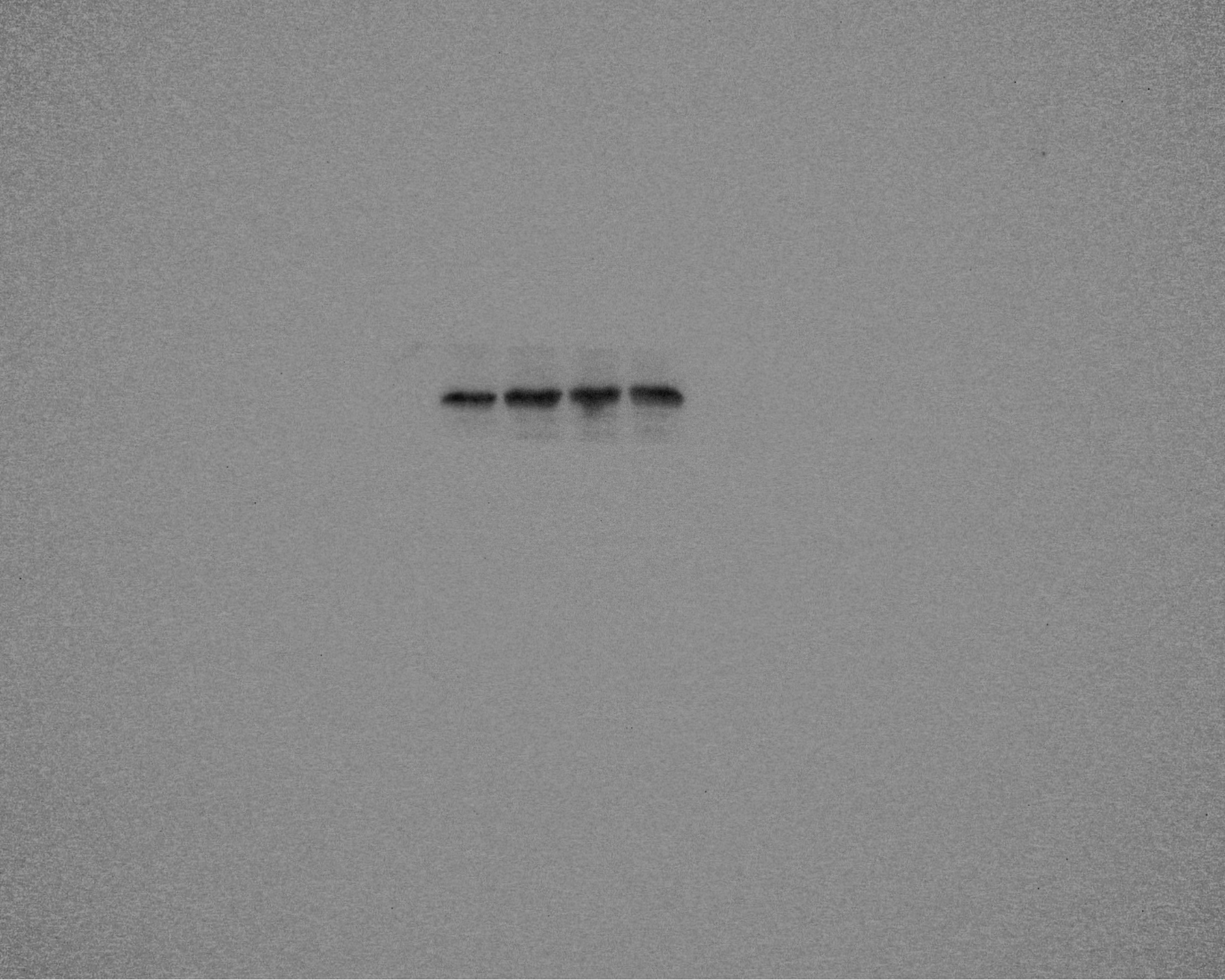

Supplement: Figure 5—source data 1. [file elife-88256-fig5-data1.zip › Figure 5-source data 1. Raw unedited blots for (Figure 5)/Figure 5F/GAPDH.tif]

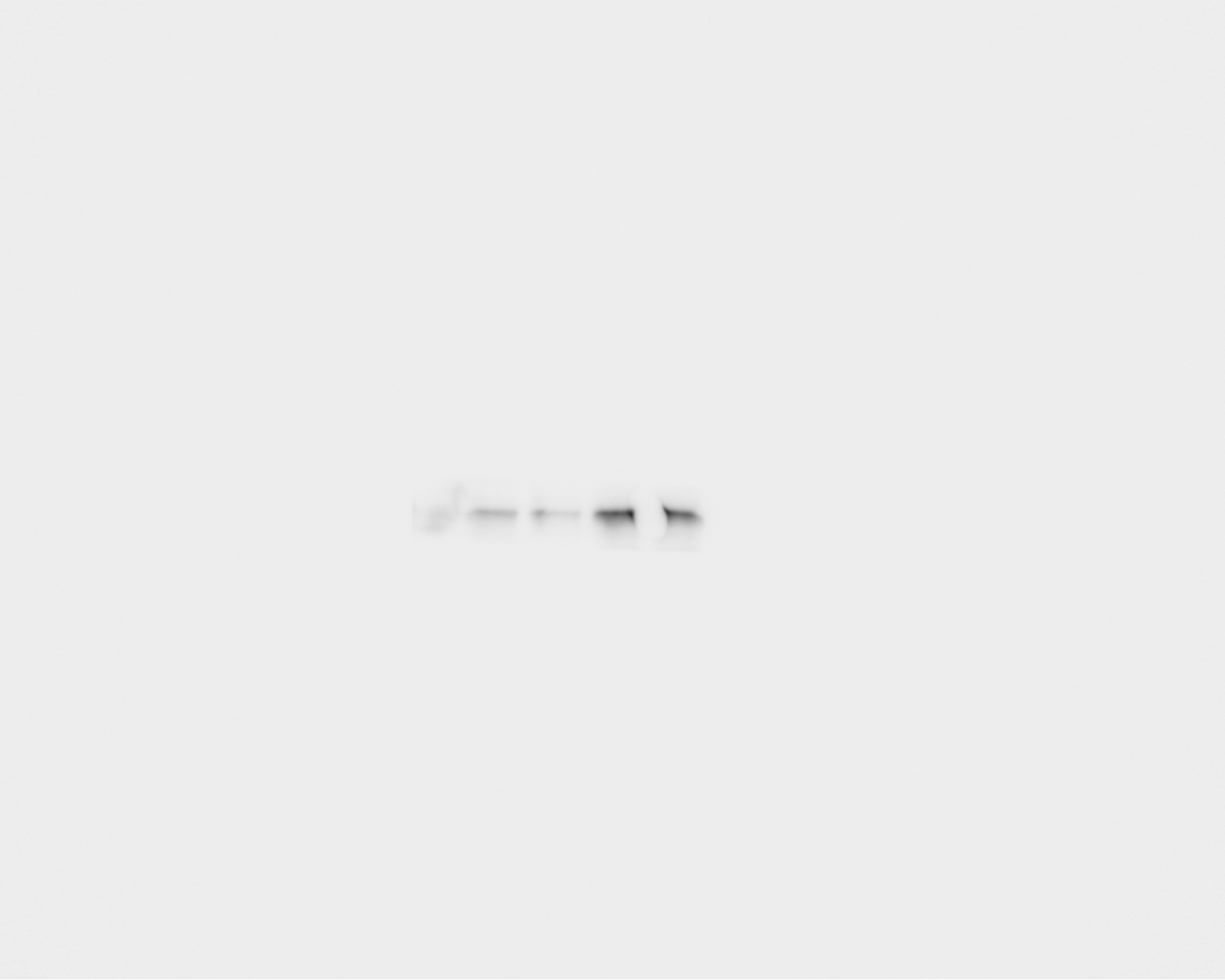

Supplement: Figure 5—source data 1. [file elife-88256-fig5-data1.zip › Figure 5-source data 1. Raw unedited blots for (Figure 5)/Figure 5F/p53.jpg]

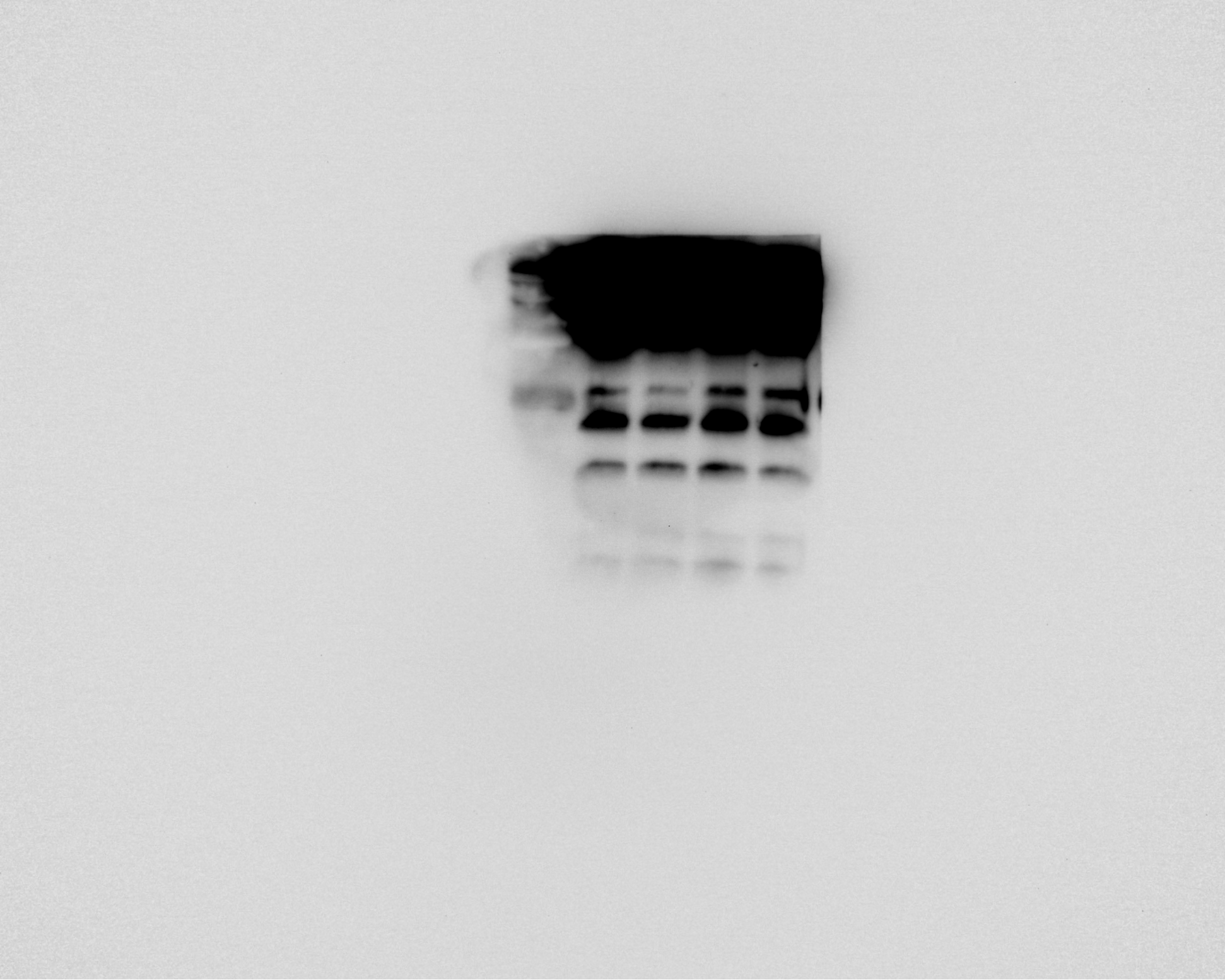

Supplement: Figure 5—source data 1. [file elife-88256-fig5-data1.zip › Figure 5-source data 1. Raw unedited blots for (Figure 5)/Figure 5F/p53.tif]

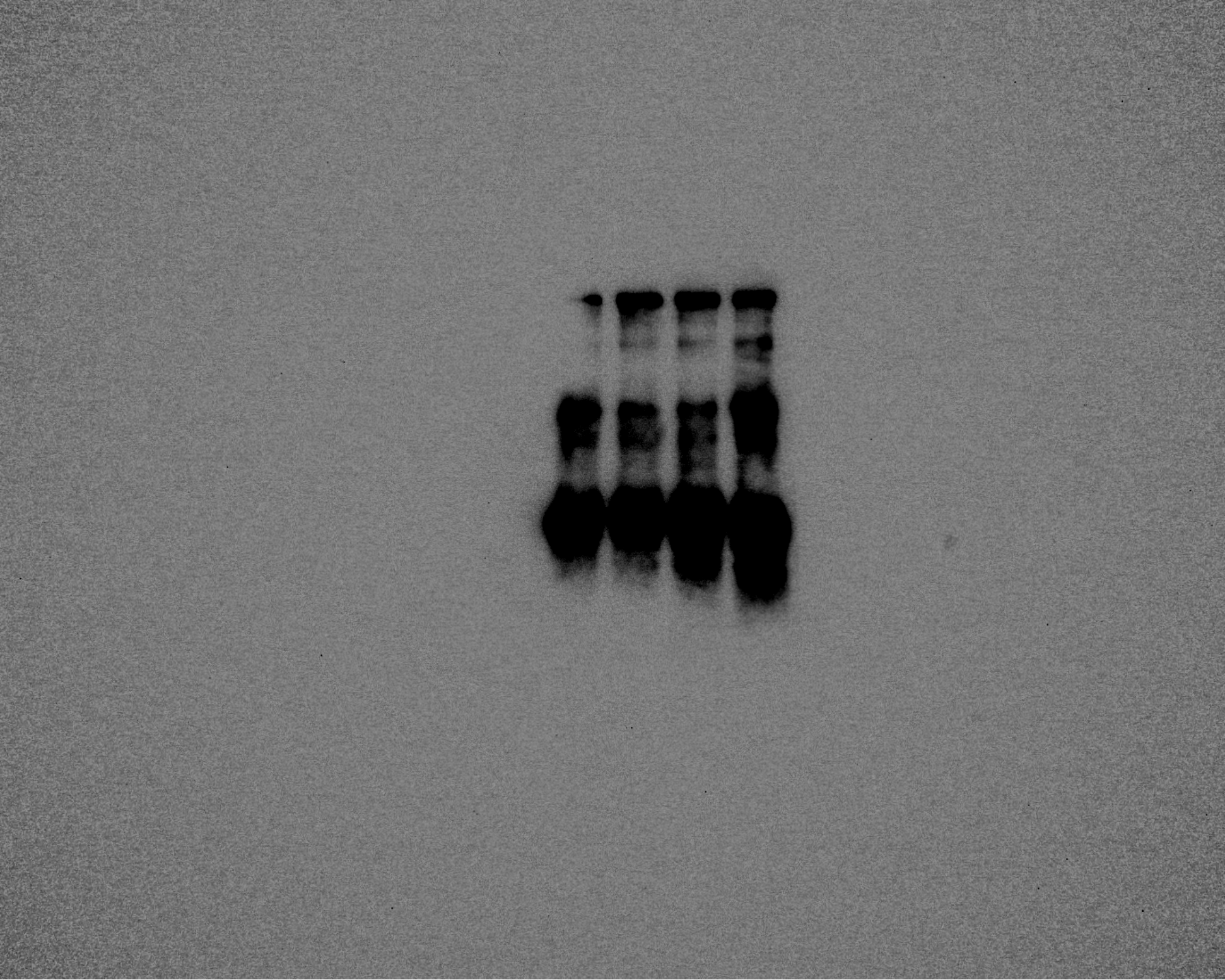

Supplement: Figure 5—source data 1. [file elife-88256-fig5-data1.zip › Figure 5-source data 1. Raw unedited blots for (Figure 5)/Figure 5F/Ub-p53.tif]

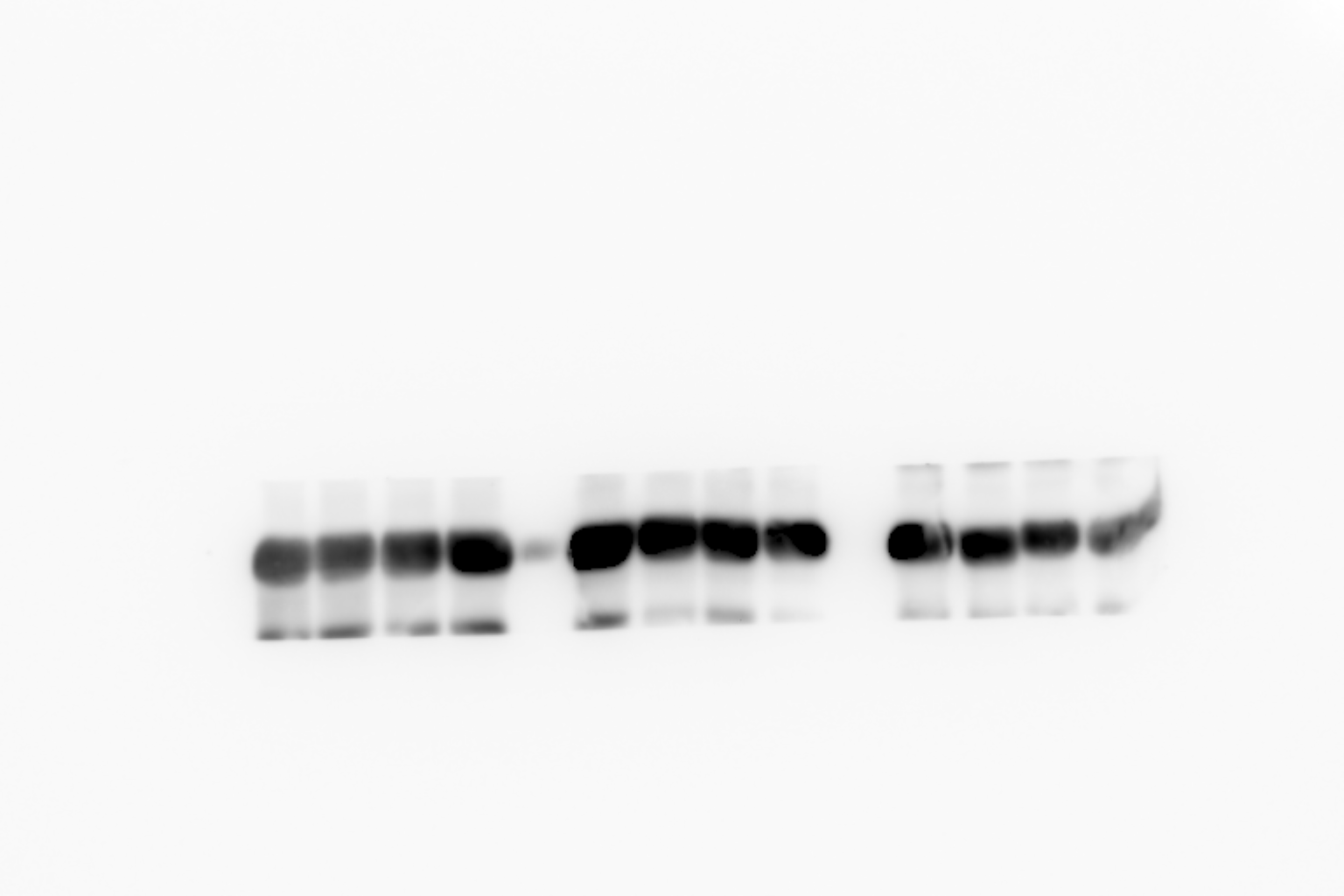

Supplement: Figure 5—source data 1. [file elife-88256-fig5-data1.zip › Figure 5-source data 1. Raw unedited blots for (Figure 5)/Figure 5J/GAPDH.tif]

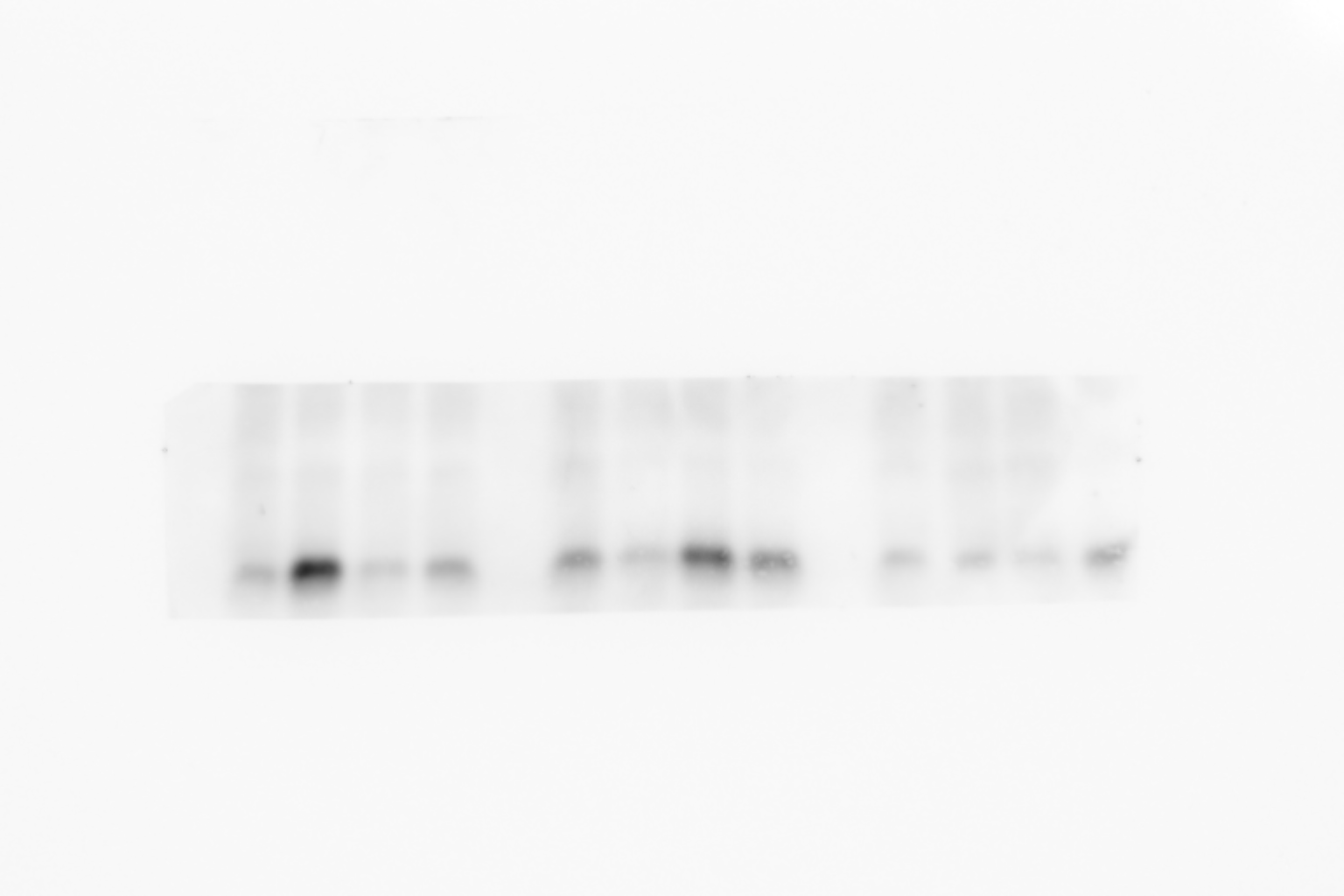

Supplement: Figure 5—source data 1. [file elife-88256-fig5-data1.zip › Figure 5-source data 1. Raw unedited blots for (Figure 5)/Figure 5J/p21.tif]

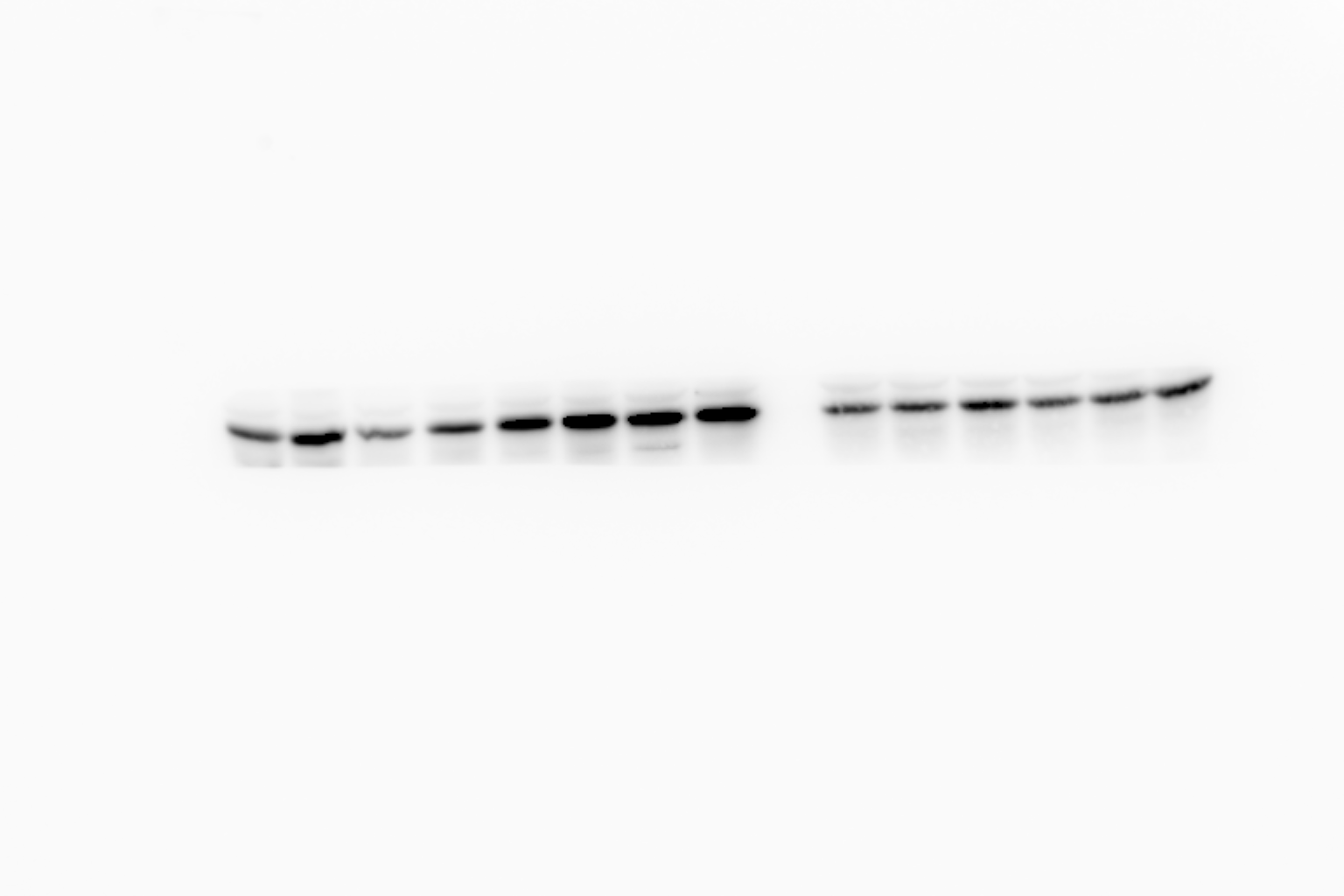

Supplement: Figure 5—source data 1. [file elife-88256-fig5-data1.zip › Figure 5-source data 1. Raw unedited blots for (Figure 5)/Figure 5J/p53.tif]

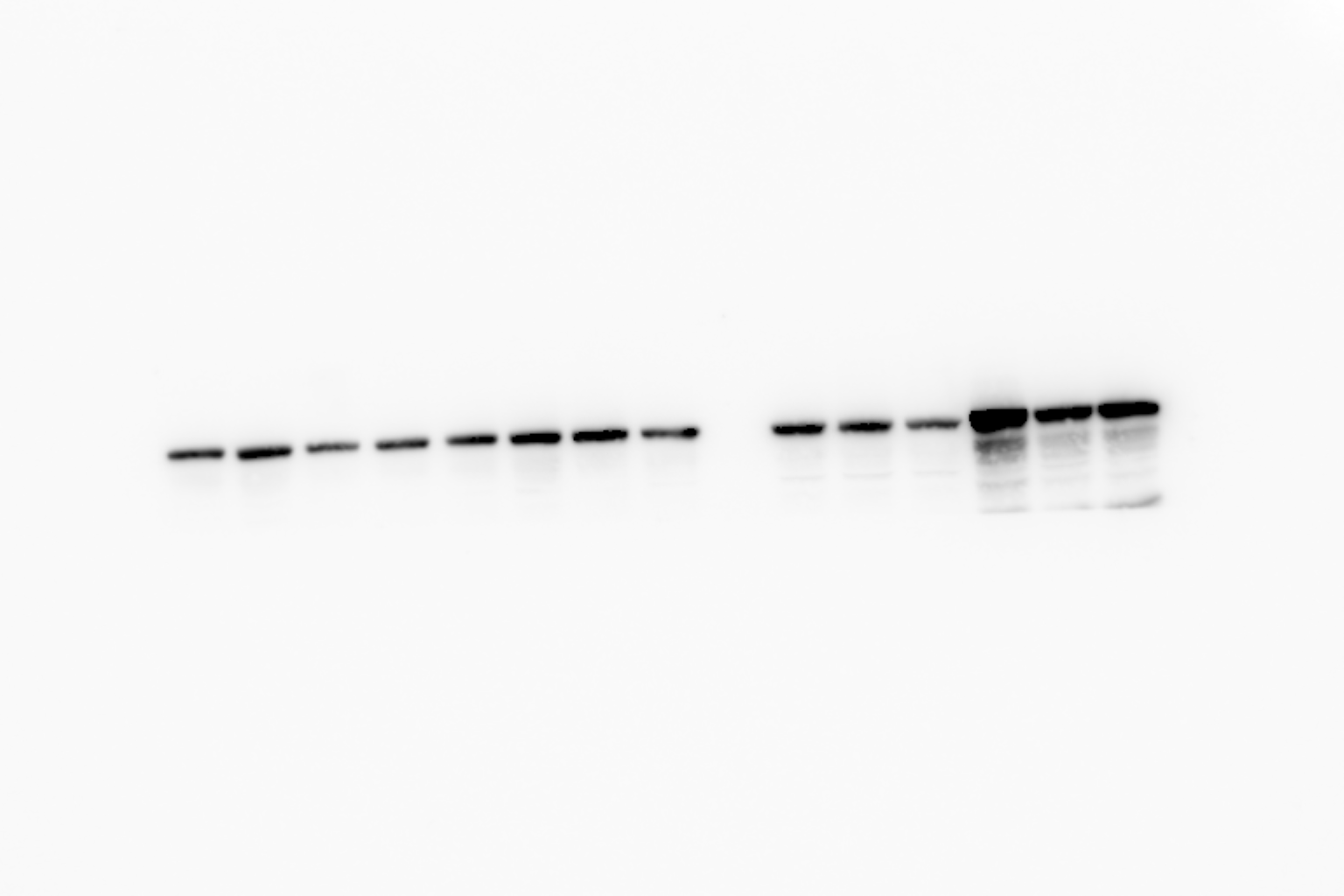

Supplement: Figure 5—source data 1. [file elife-88256-fig5-data1.zip › Figure 5-source data 1. Raw unedited blots for (Figure 5)/Figure 5J/TRIM28.tif]

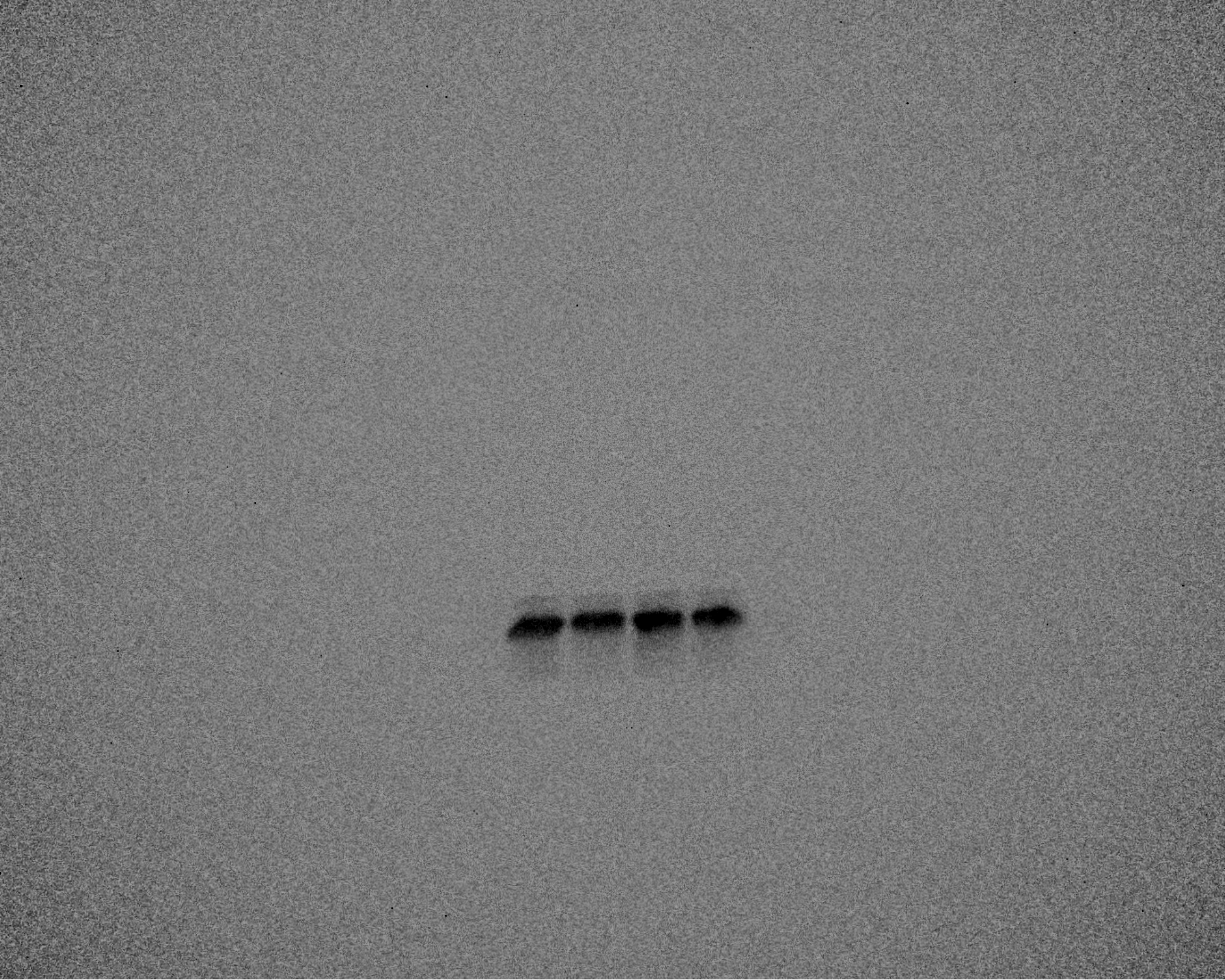

Supplement: Figure 5—source data 1. [file elife-88256-fig5-data1.zip › Figure 5-source data 1. Raw unedited blots for (Figure 5)/Figure 5N/GAPDH.jpg]

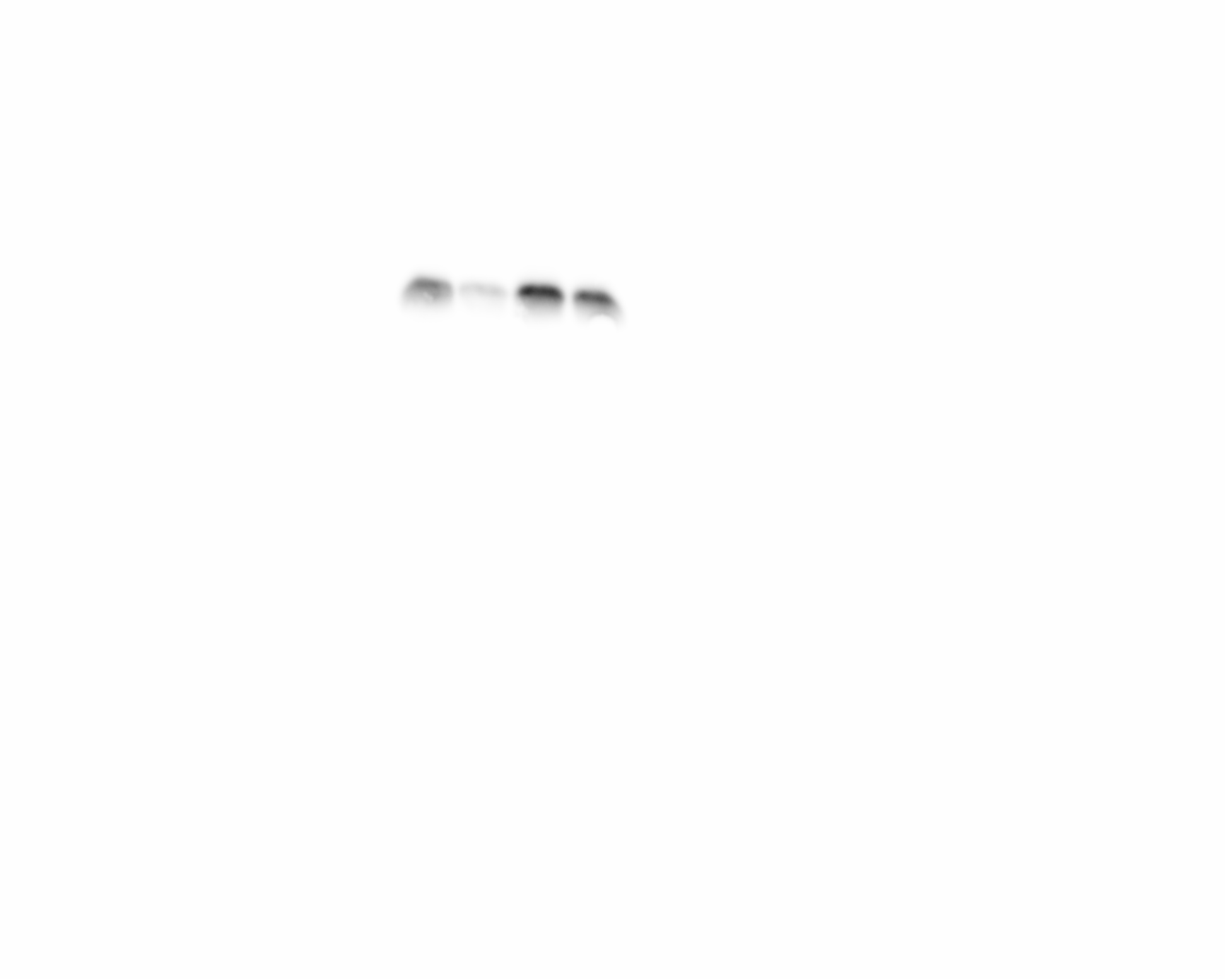

Supplement: Figure 5—source data 1. [file elife-88256-fig5-data1.zip › Figure 5-source data 1. Raw unedited blots for (Figure 5)/Figure 5N/p21.tif]

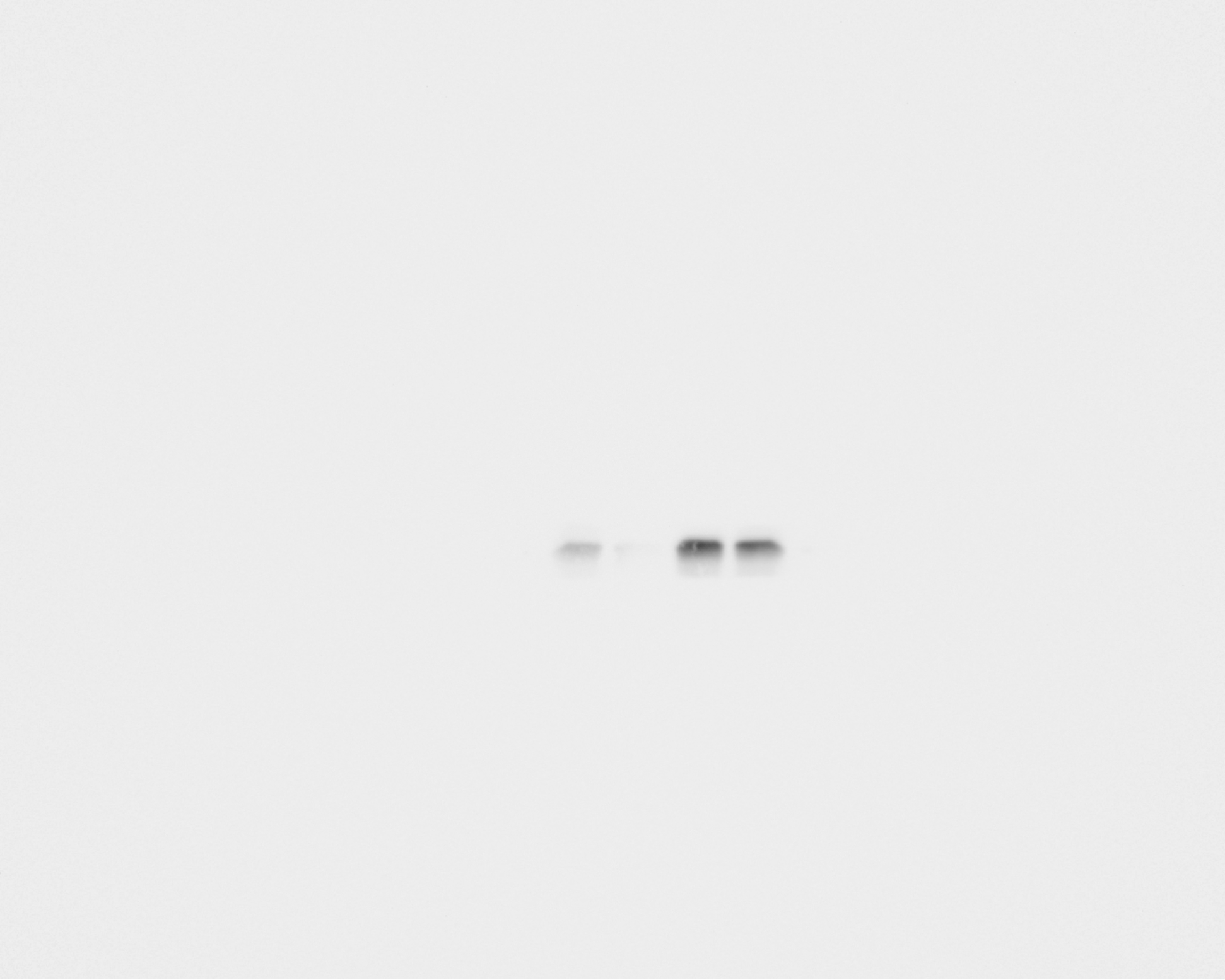

Supplement: Figure 5—source data 1. [file elife-88256-fig5-data1.zip › Figure 5-source data 1. Raw unedited blots for (Figure 5)/Figure 5N/p53.jpg]

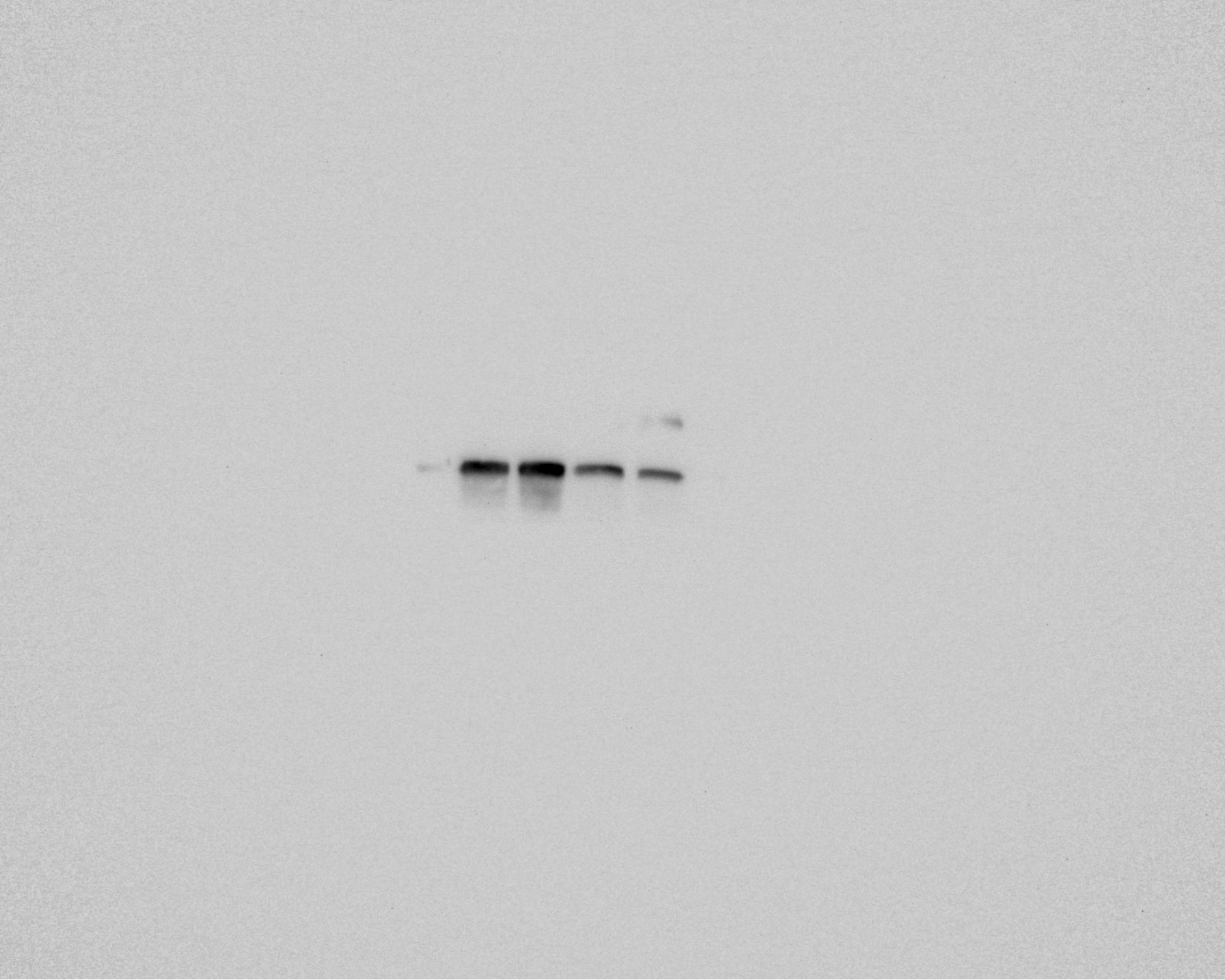

Supplement: Figure 5—source data 1. [file elife-88256-fig5-data1.zip › Figure 5-source data 1. Raw unedited blots for (Figure 5)/Figure 5N/TRIM28.jpg]

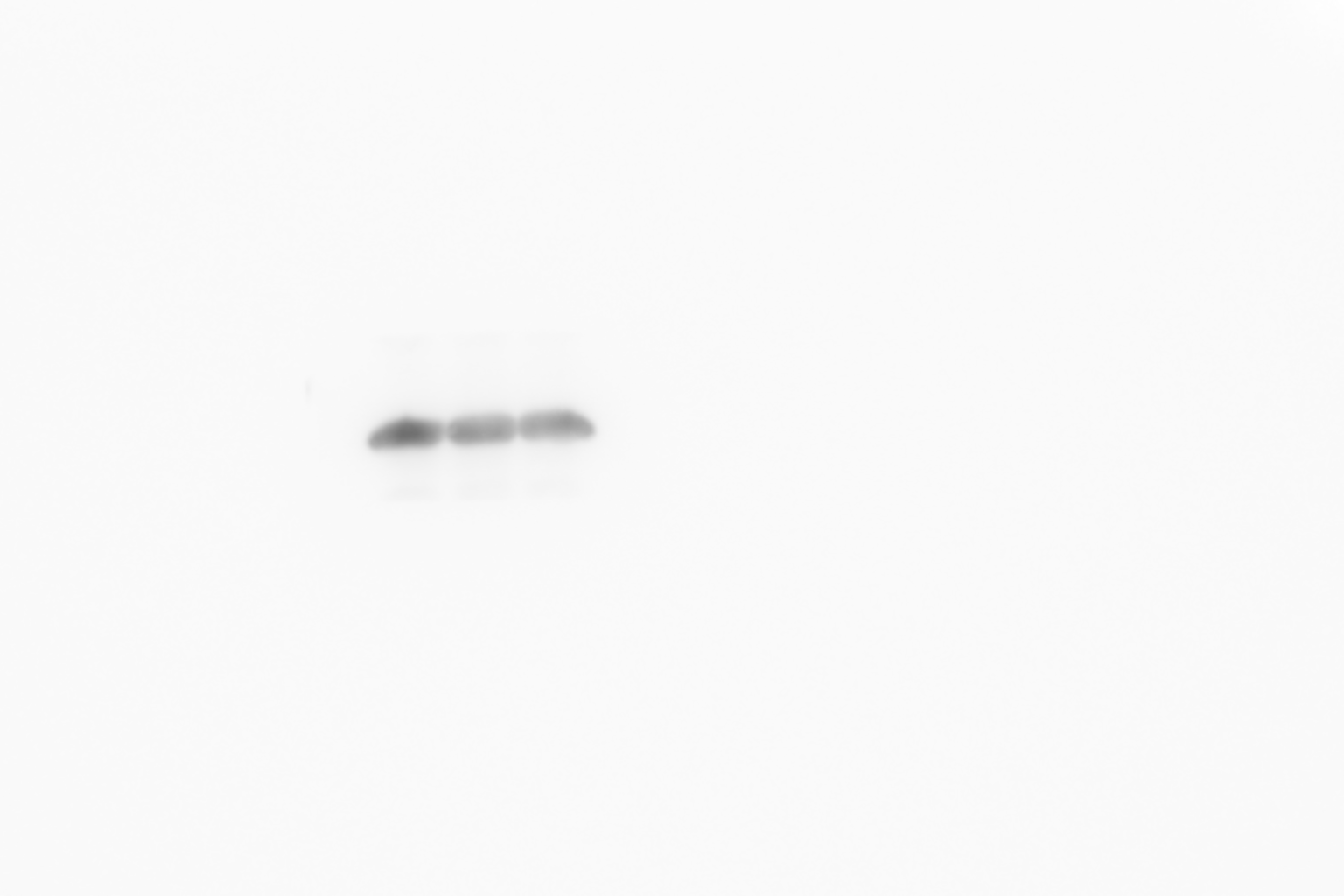

Supplement: Figure 5—figure supplement 1—source data 1. [file elife-88256-fig5-figsupp1-data1.zip › Figure 5-figure supplement 1-source data 1. Raw unedited blots for (Figure 5-figure supplement 1)/Figure 5-figure supplement 1C/GAPDH.tif]

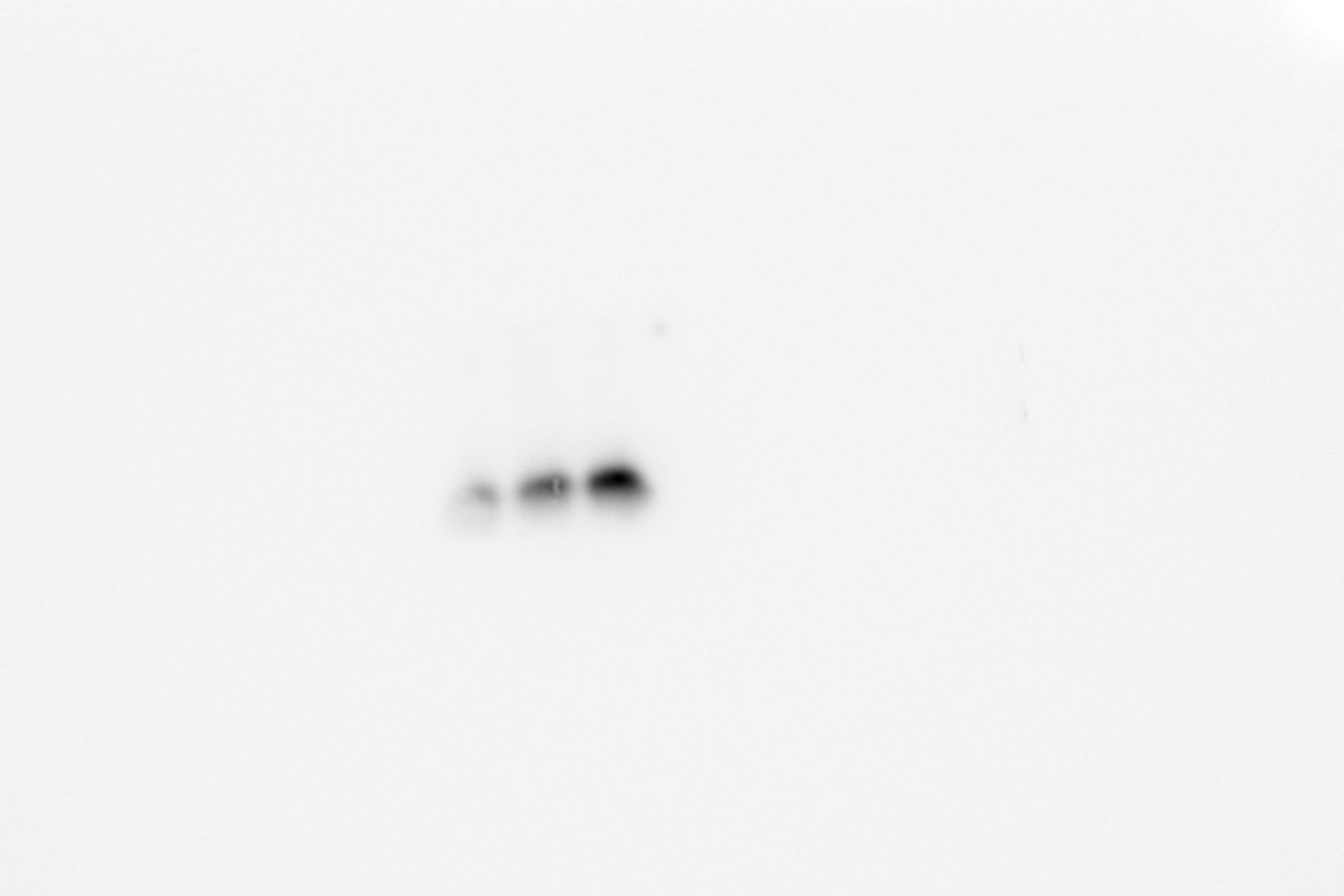

Supplement: Figure 5—figure supplement 1—source data 1. [file elife-88256-fig5-figsupp1-data1.zip › Figure 5-figure supplement 1-source data 1. Raw unedited blots for (Figure 5-figure supplement 1)/Figure 5-figure supplement 1C/p21.tif]

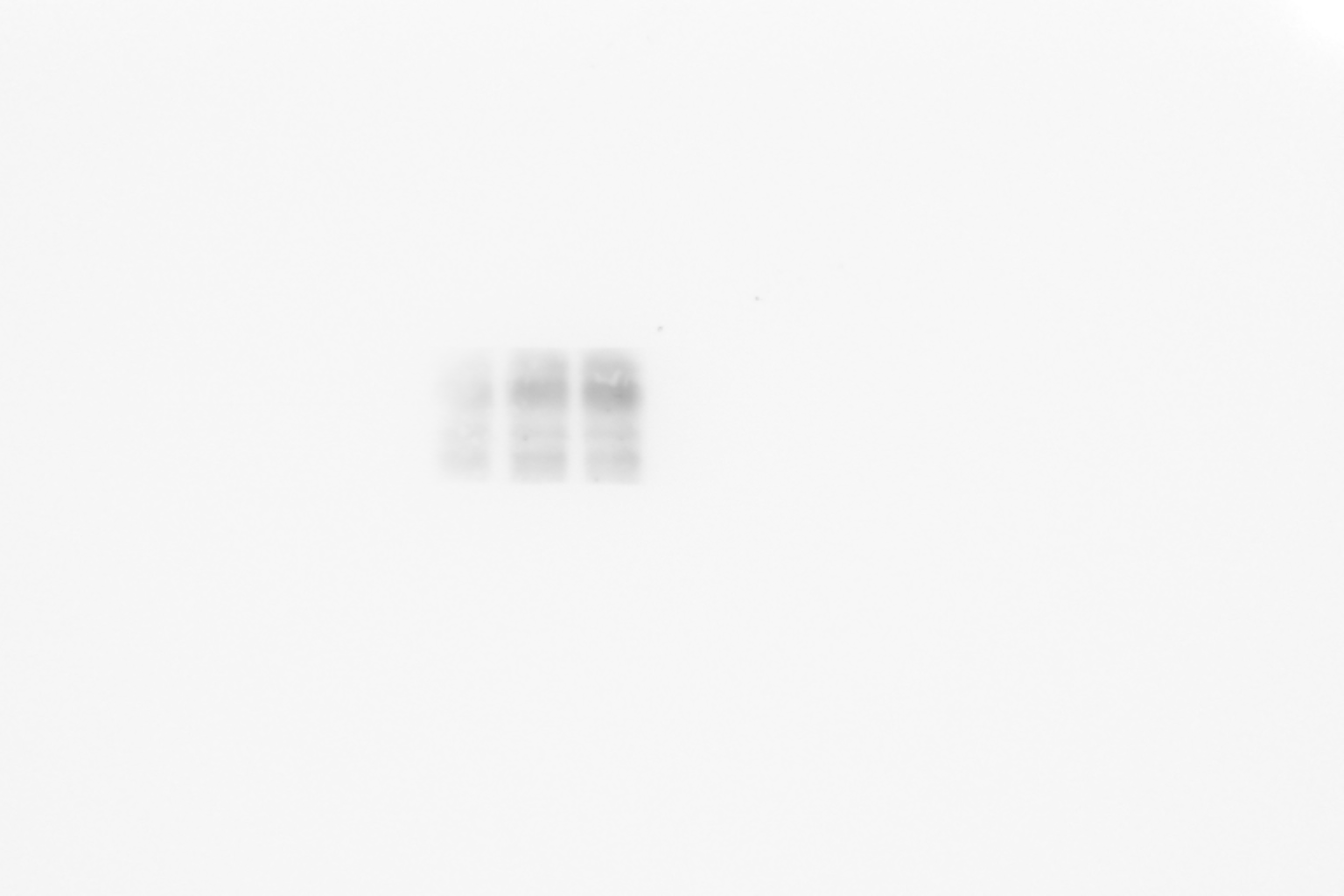

Supplement: Figure 5—figure supplement 1—source data 1. [file elife-88256-fig5-figsupp1-data1.zip › Figure 5-figure supplement 1-source data 1. Raw unedited blots for (Figure 5-figure supplement 1)/Figure 5-figure supplement 1C/p53.tif]

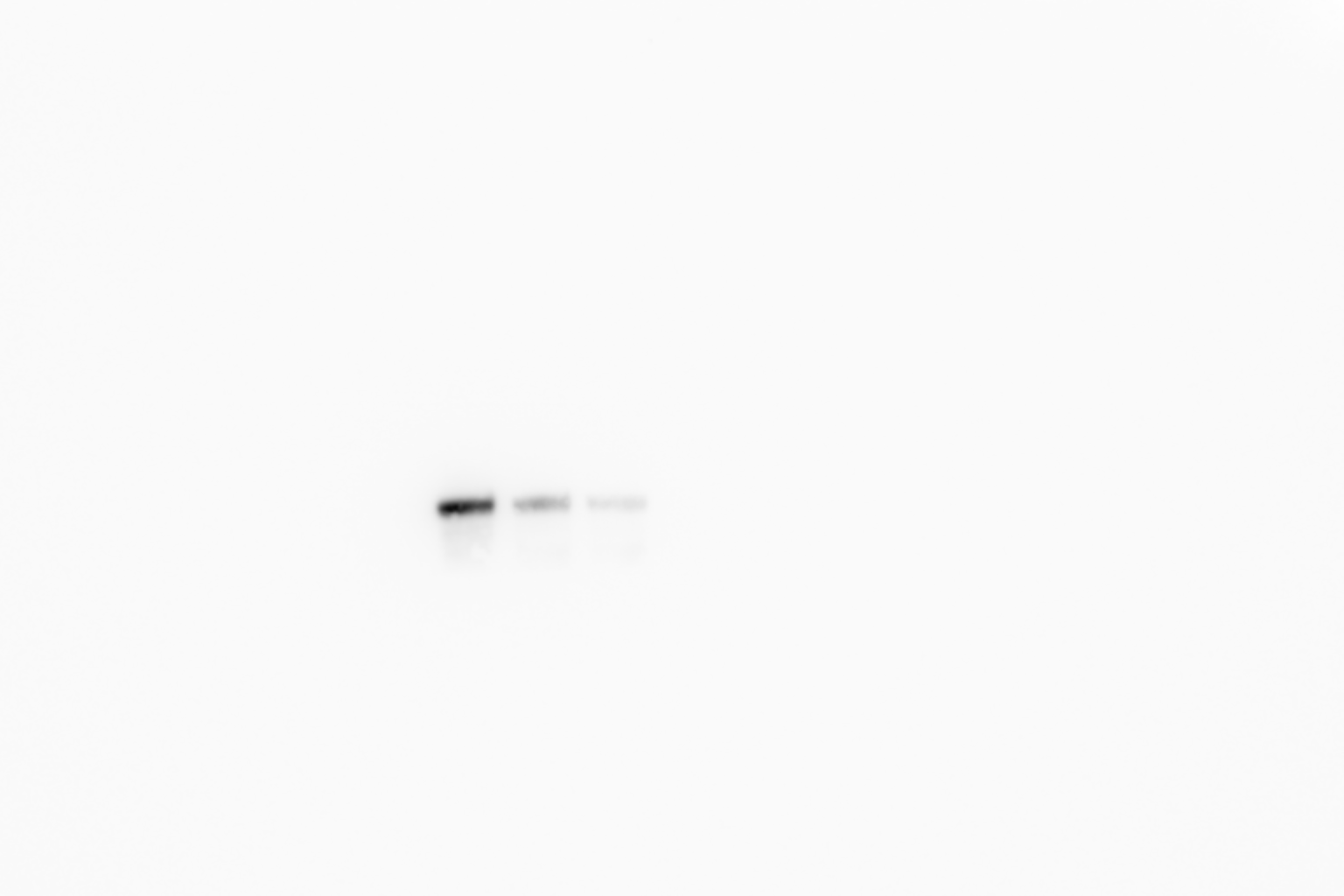

Supplement: Figure 5—figure supplement 1—source data 1. [file elife-88256-fig5-figsupp1-data1.zip › Figure 5-figure supplement 1-source data 1. Raw unedited blots for (Figure 5-figure supplement 1)/Figure 5-figure supplement 1C/TRIM28.tif]

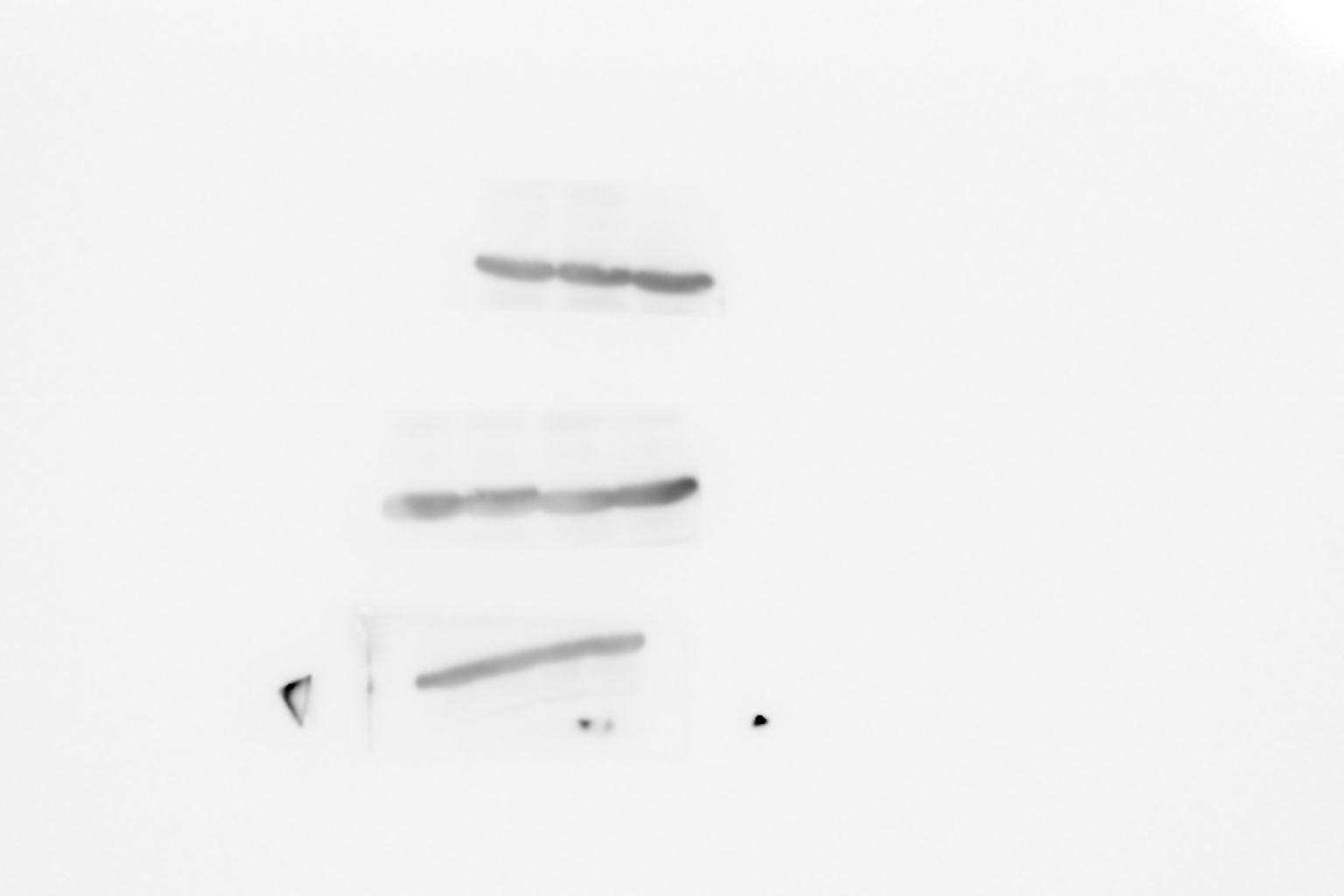

Supplement: Figure 5—figure supplement 1—source data 1. [file elife-88256-fig5-figsupp1-data1.zip › Figure 5-figure supplement 1-source data 1. Raw unedited blots for (Figure 5-figure supplement 1)/Figure 5-figure supplement 1E/GAPDH.tif]

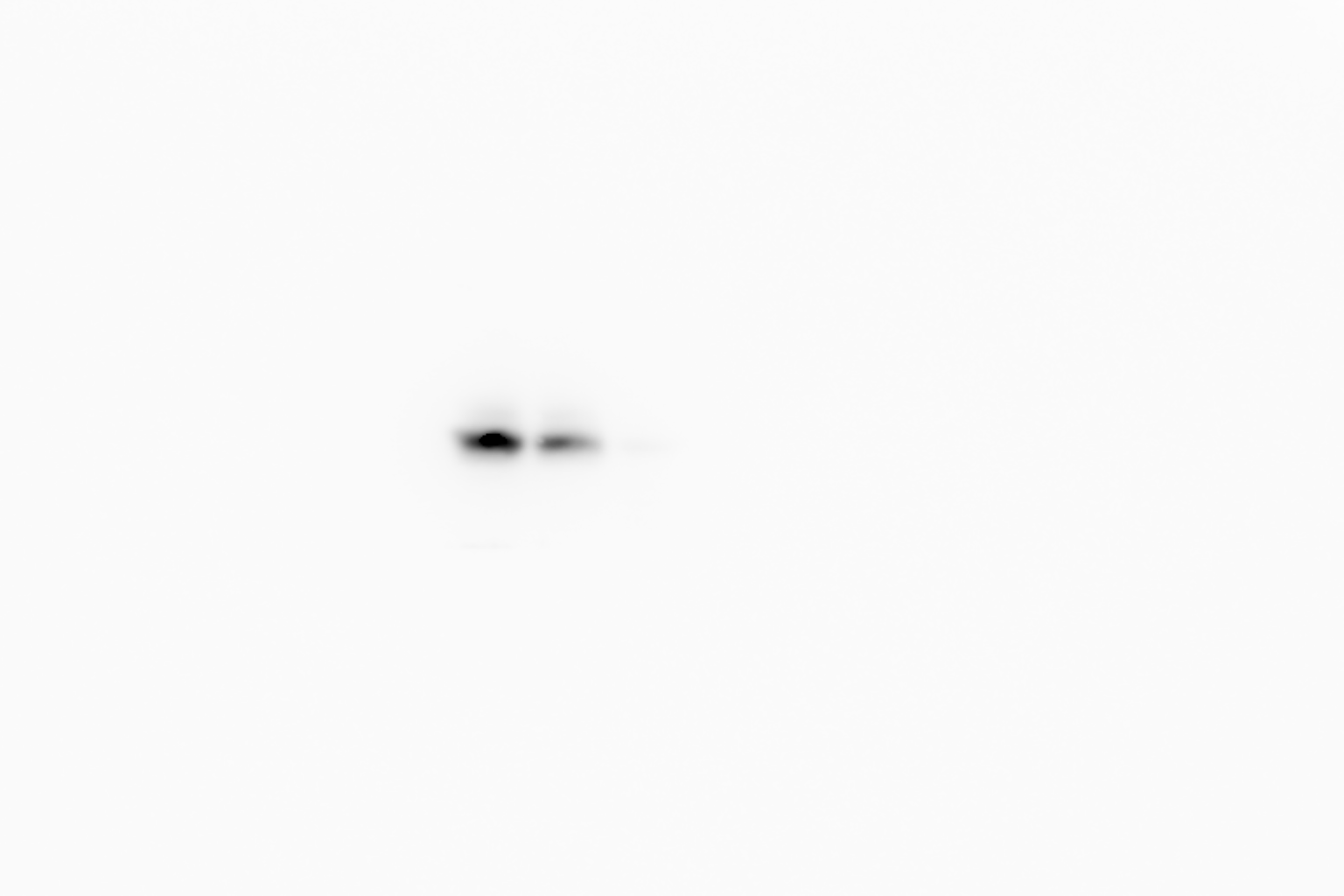

Supplement: Figure 5—figure supplement 1—source data 1. [file elife-88256-fig5-figsupp1-data1.zip › Figure 5-figure supplement 1-source data 1. Raw unedited blots for (Figure 5-figure supplement 1)/Figure 5-figure supplement 1E/p21.tif]

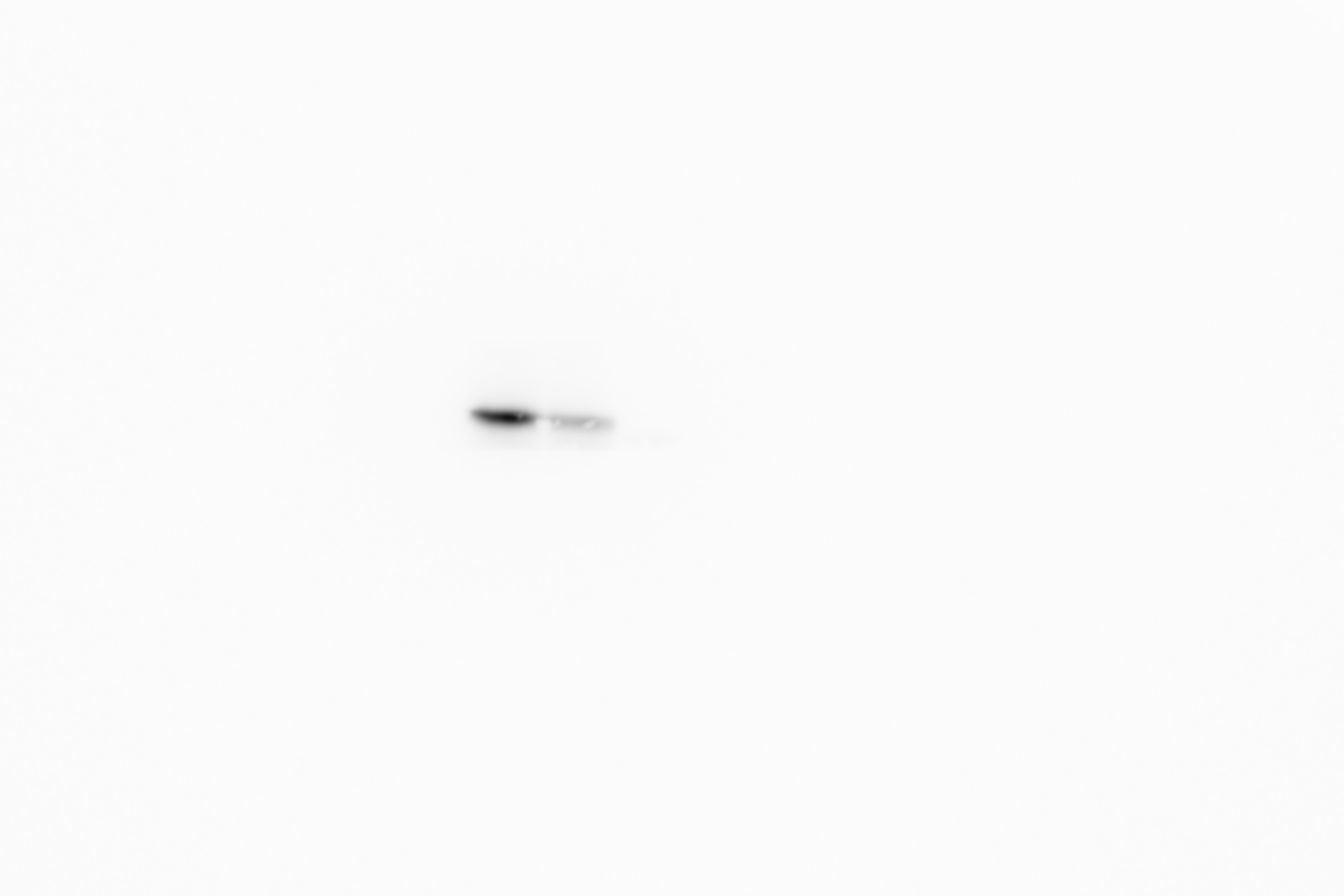

Supplement: Figure 5—figure supplement 1—source data 1. [file elife-88256-fig5-figsupp1-data1.zip › Figure 5-figure supplement 1-source data 1. Raw unedited blots for (Figure 5-figure supplement 1)/Figure 5-figure supplement 1E/p53.tif]

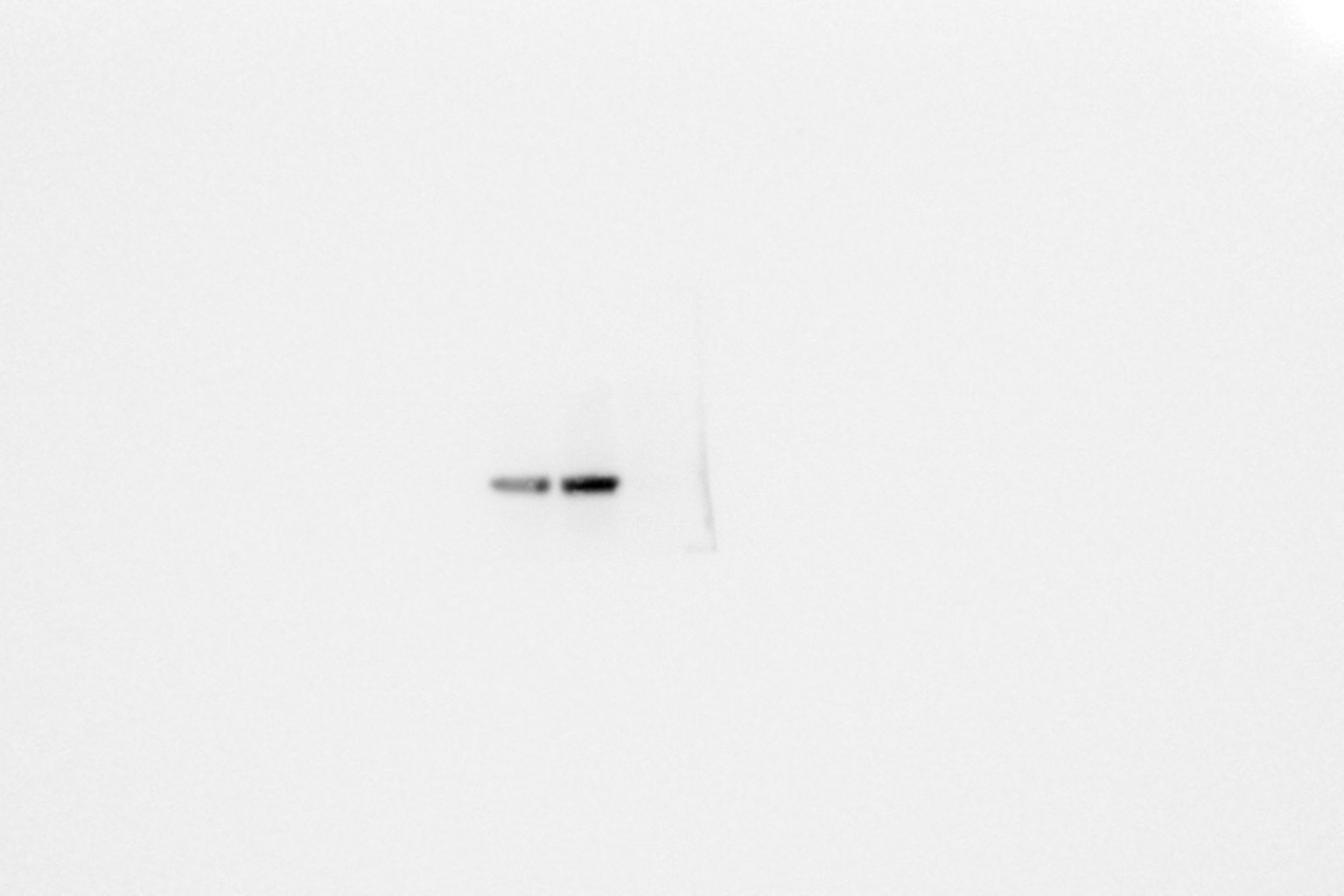

Supplement: Figure 5—figure supplement 1—source data 1. [file elife-88256-fig5-figsupp1-data1.zip › Figure 5-figure supplement 1-source data 1. Raw unedited blots for (Figure 5-figure supplement 1)/Figure 5-figure supplement 1E/TRIM28.tif]

Figure 5-figure supplement 1C:

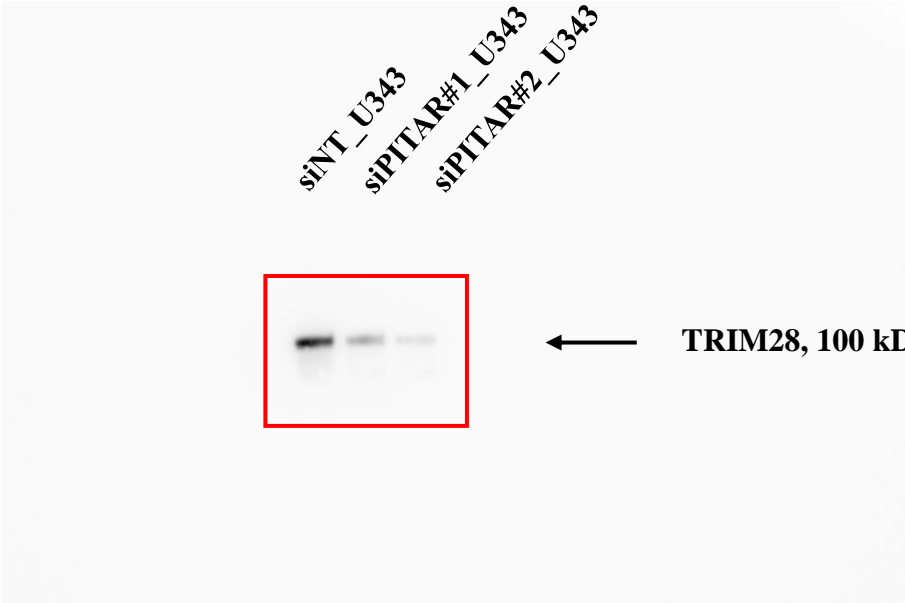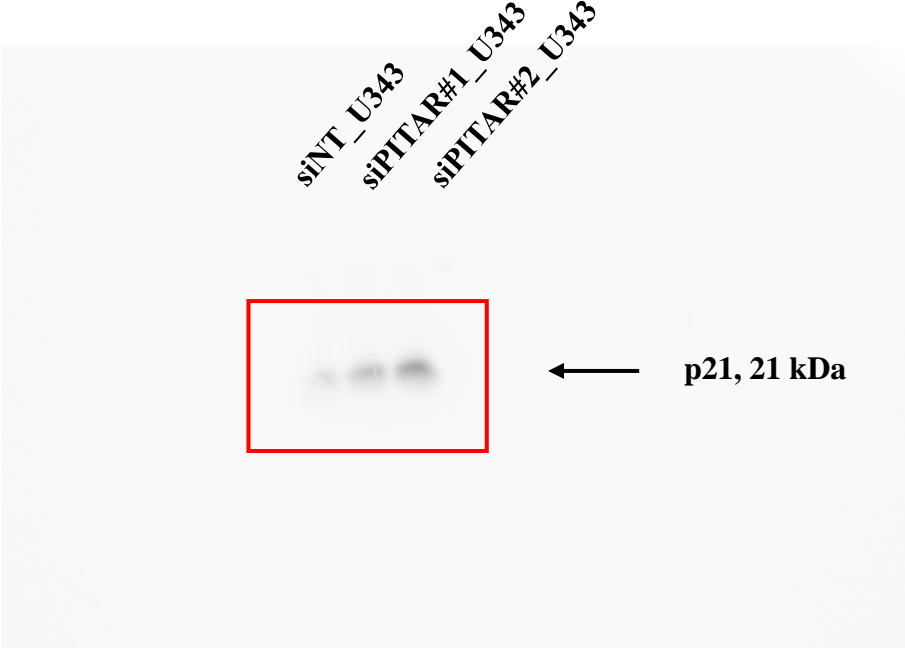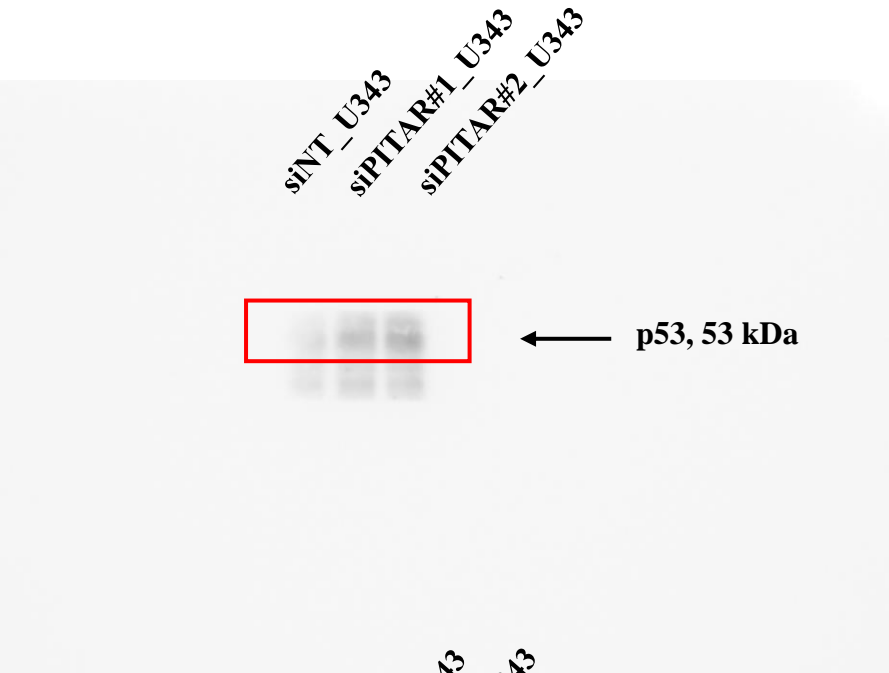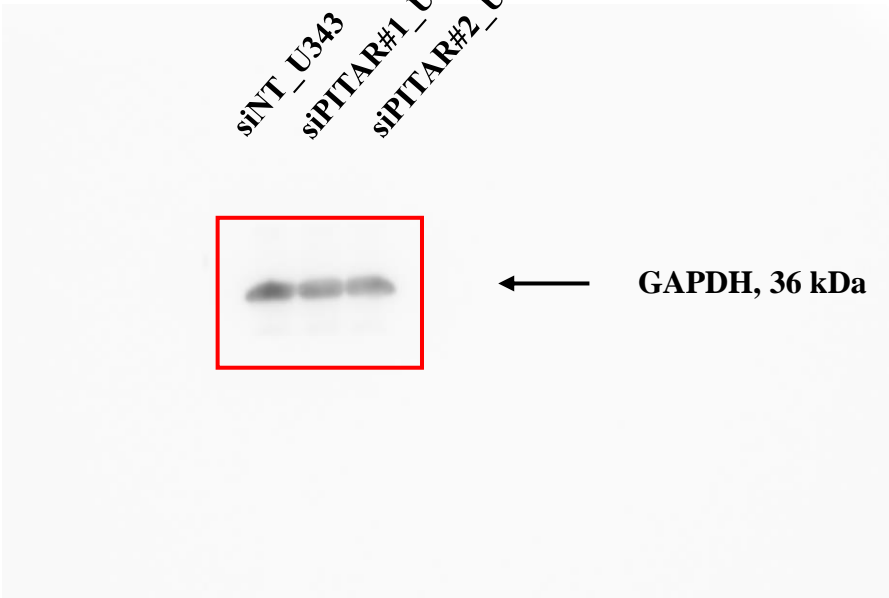

Figure 5-figure supplement 1E:

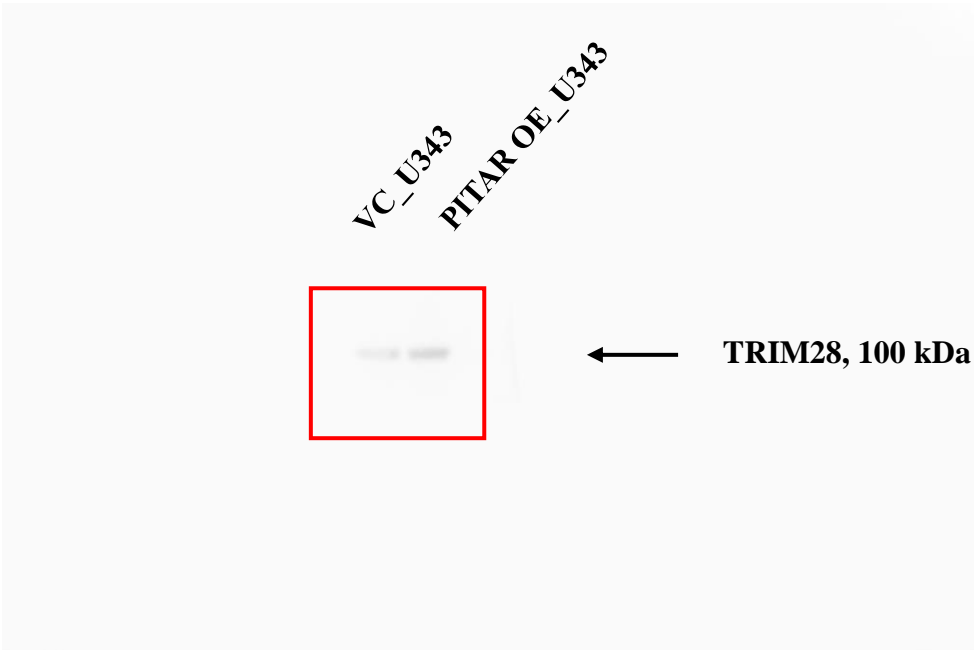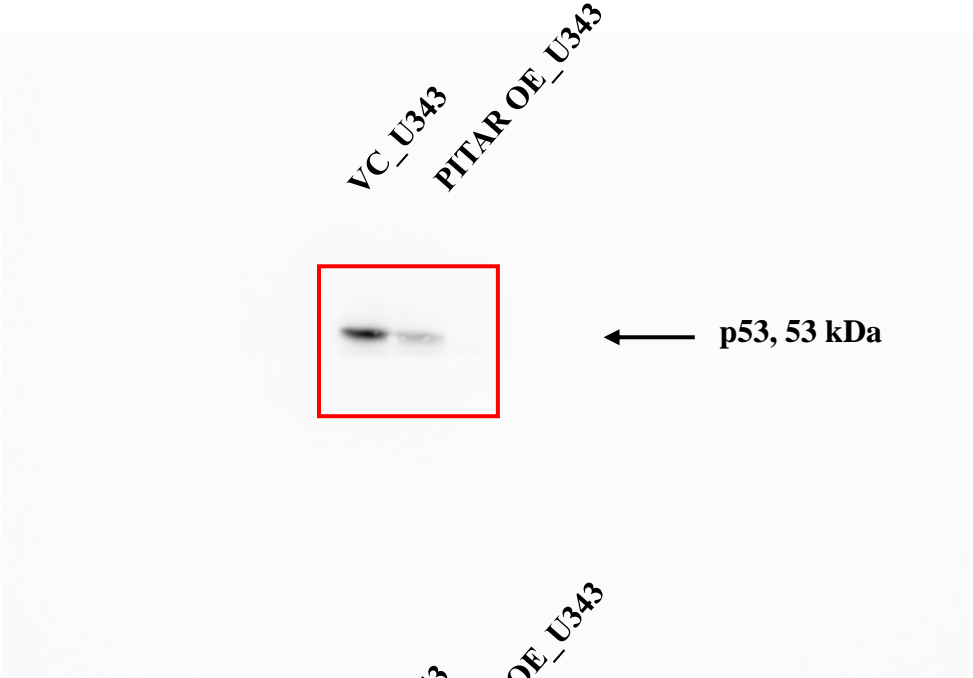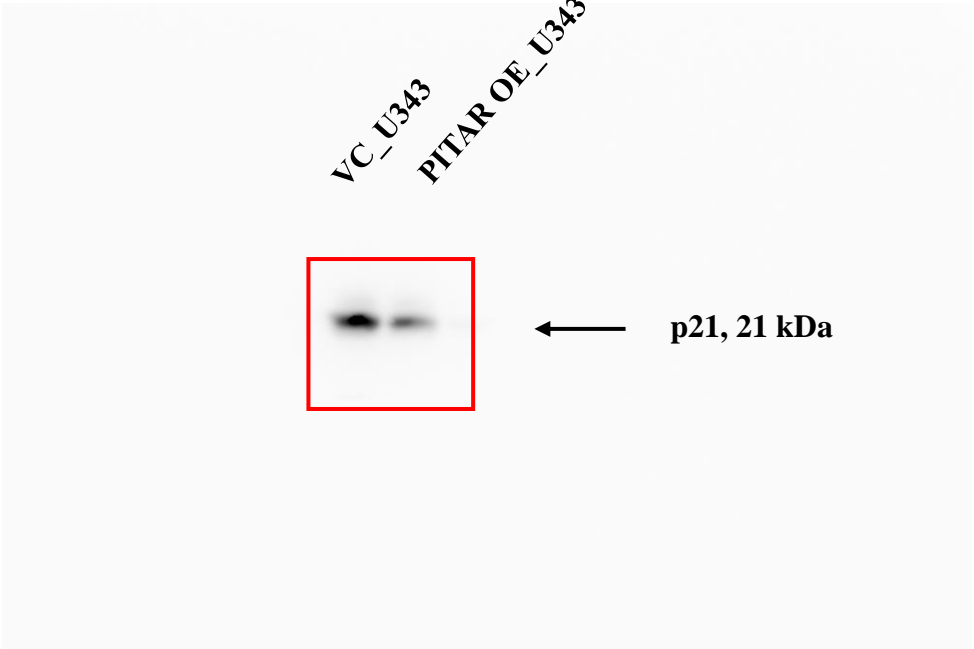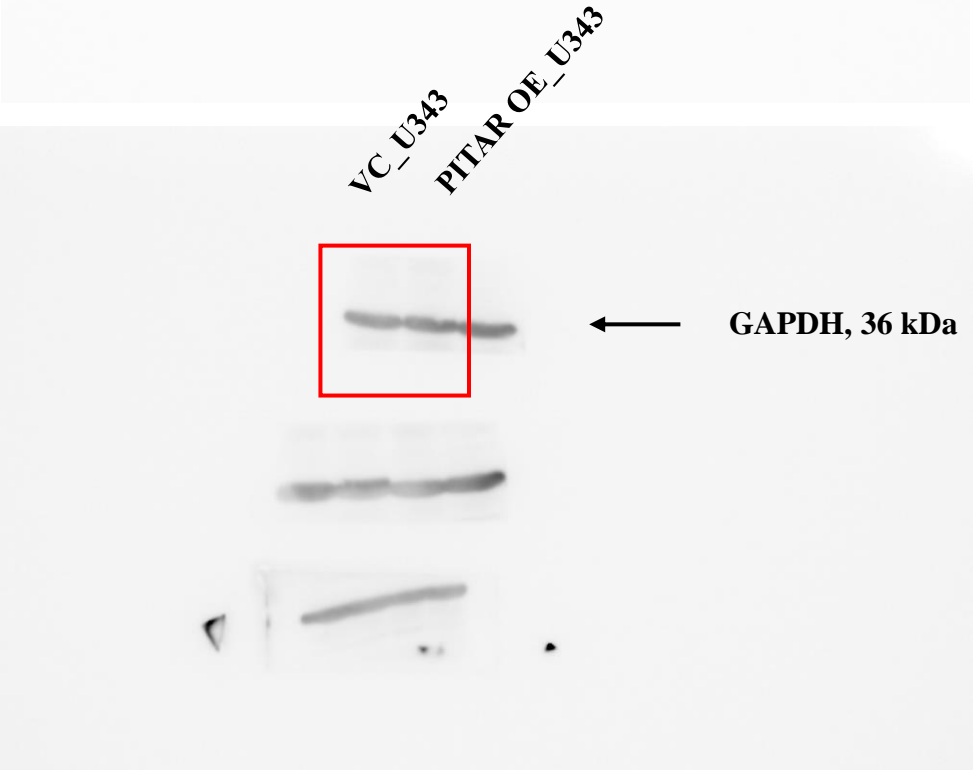

Supplement: Figure 5—figure supplement 1—source data 2. [file elife-88256-fig5-figsupp1-data2.pdf]

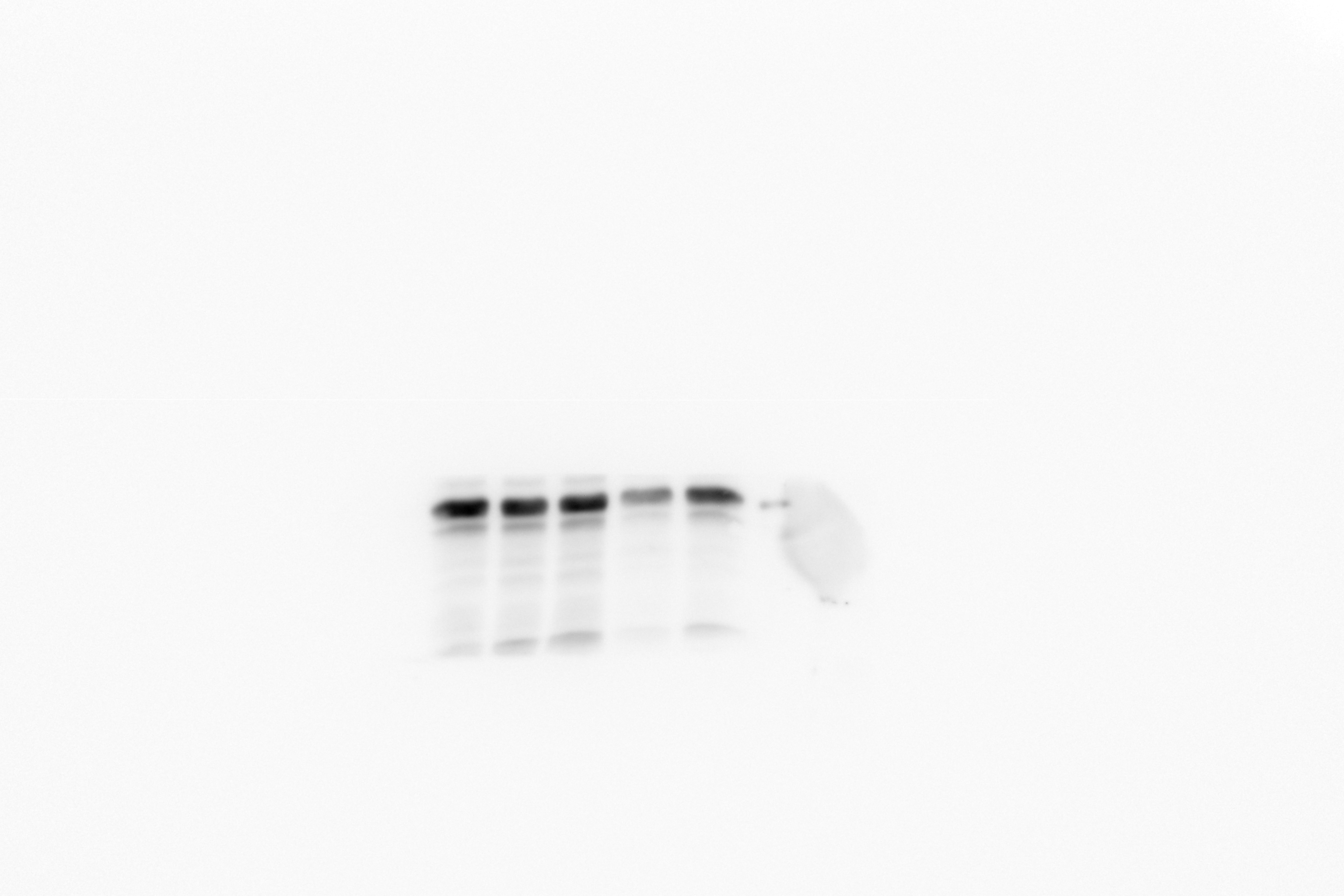

Supplement: Figure 5—figure supplement 2—source data 1. [file elife-88256-fig5-figsupp2-data1.zip › Figure 5-figure supplement 2-source data 1. Raw unedited blots for (Figure 5-figure supplement 2)/Figure 5-figure supplement 2B/GAPDH.tif]

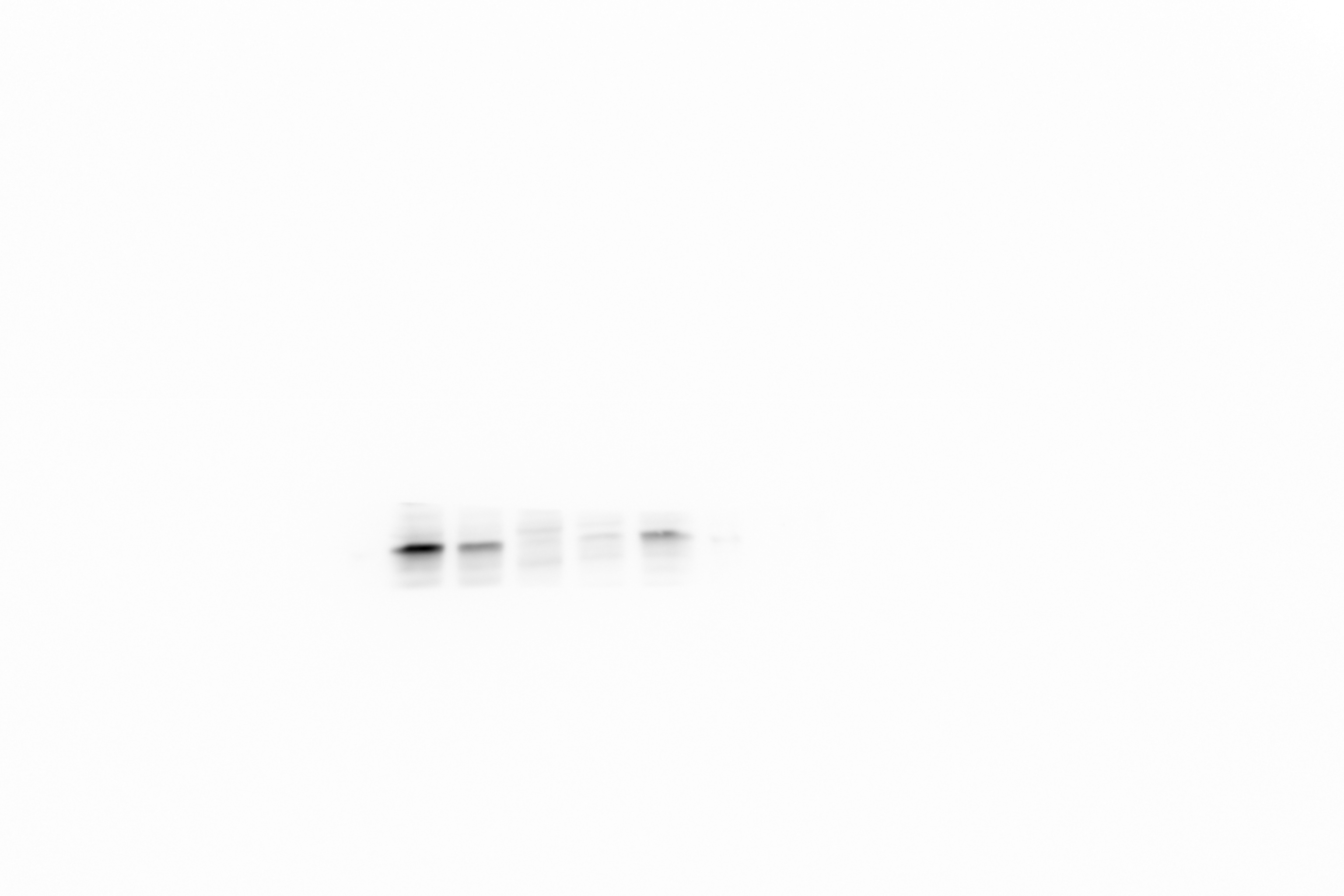

Supplement: Figure 5—figure supplement 2—source data 1. [file elife-88256-fig5-figsupp2-data1.zip › Figure 5-figure supplement 2-source data 1. Raw unedited blots for (Figure 5-figure supplement 2)/Figure 5-figure supplement 2B/p53.tif]

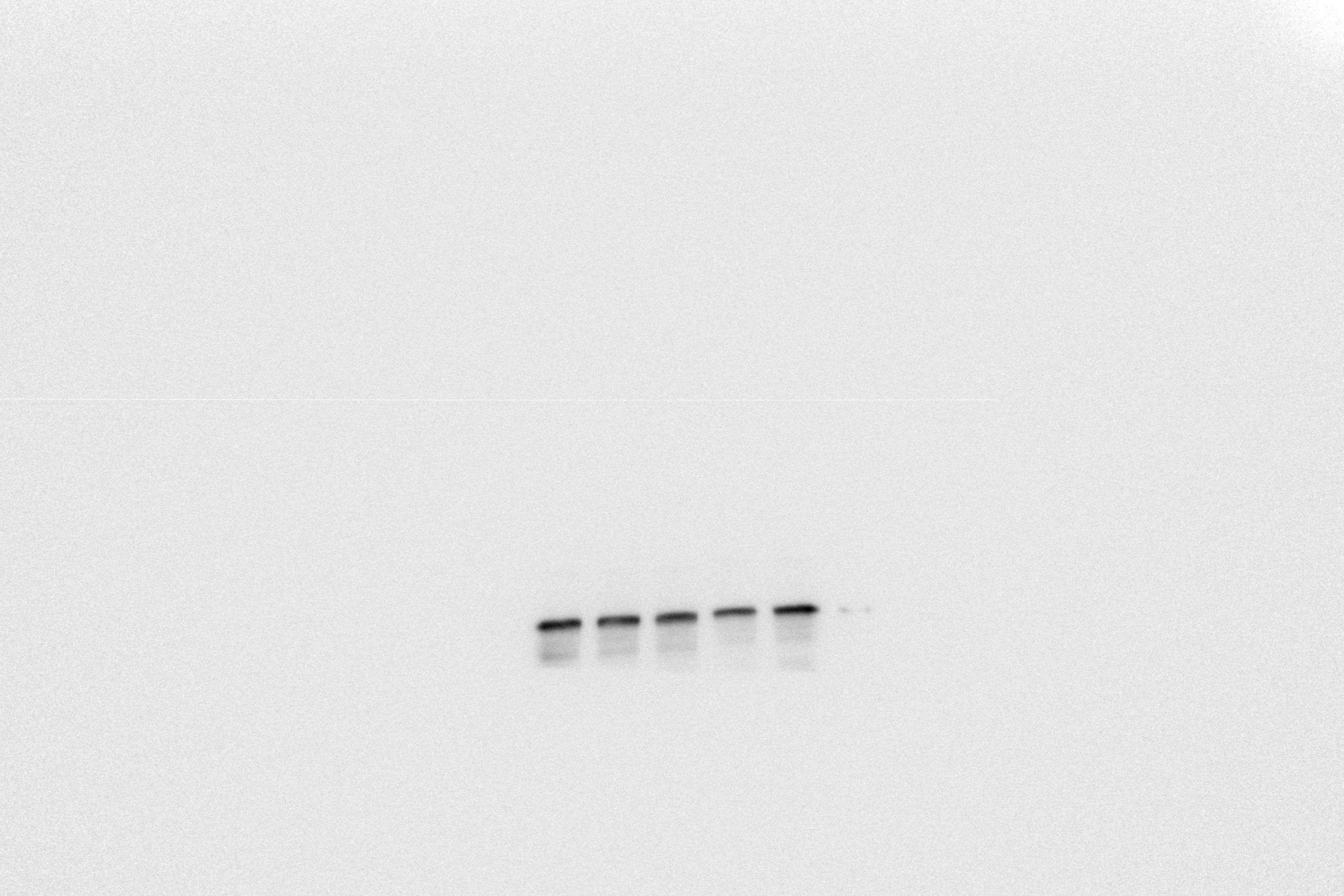

Supplement: Figure 5—figure supplement 2—source data 1. [file elife-88256-fig5-figsupp2-data1.zip › Figure 5-figure supplement 2-source data 1. Raw unedited blots for (Figure 5-figure supplement 2)/Figure 5-figure supplement 2B/TRIM28.tif]

**Figure 5-figure supplement 2B:**

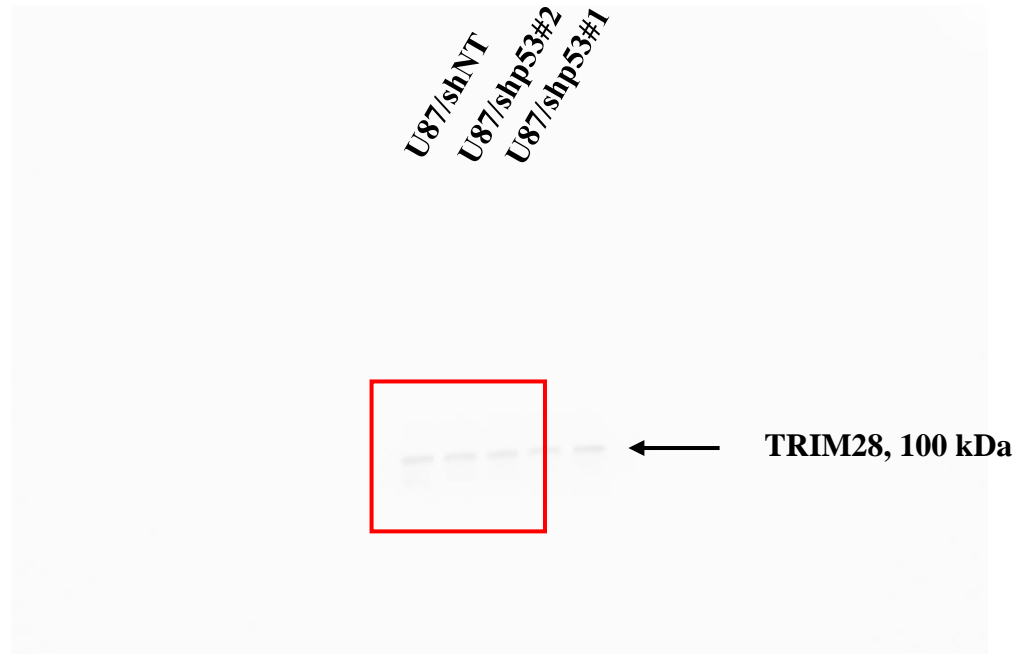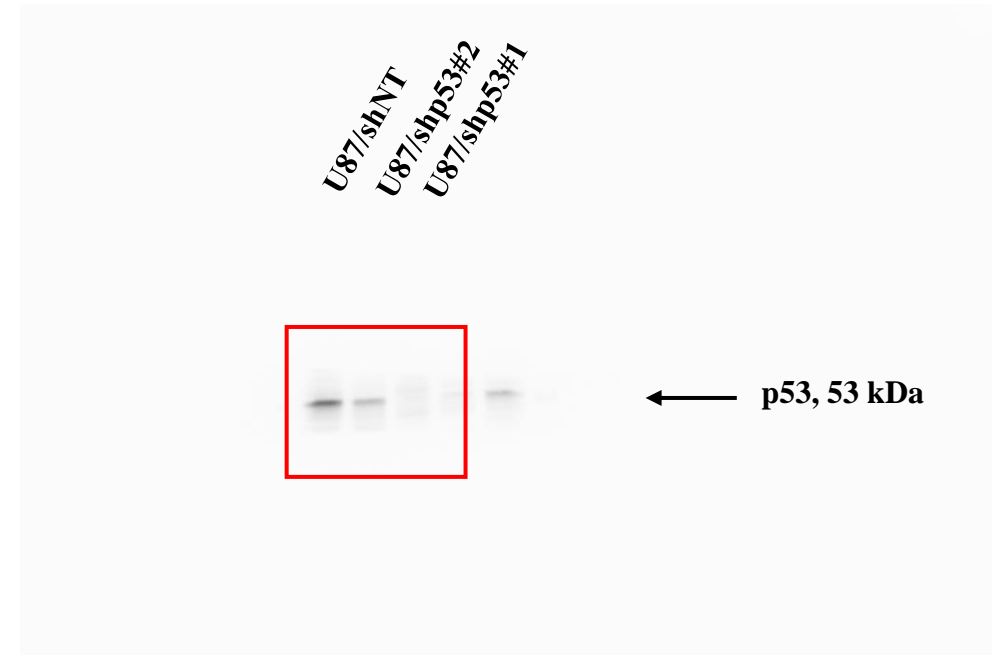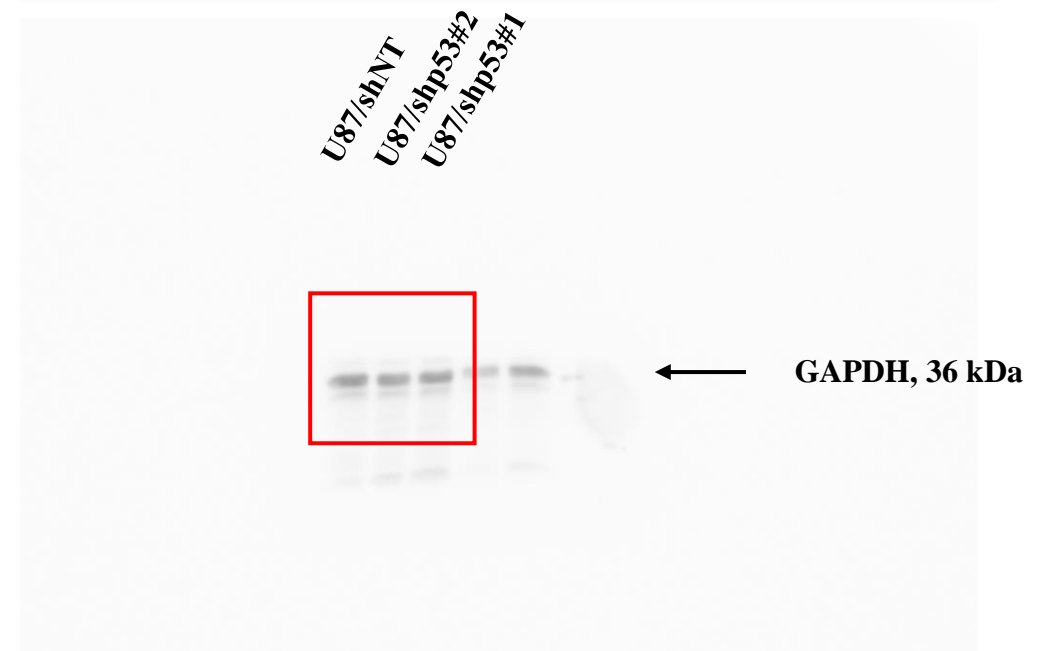

Supplement: Figure 5—figure supplement 2—source data 2. [file elife-88256-fig5-figsupp2-data2.pdf]

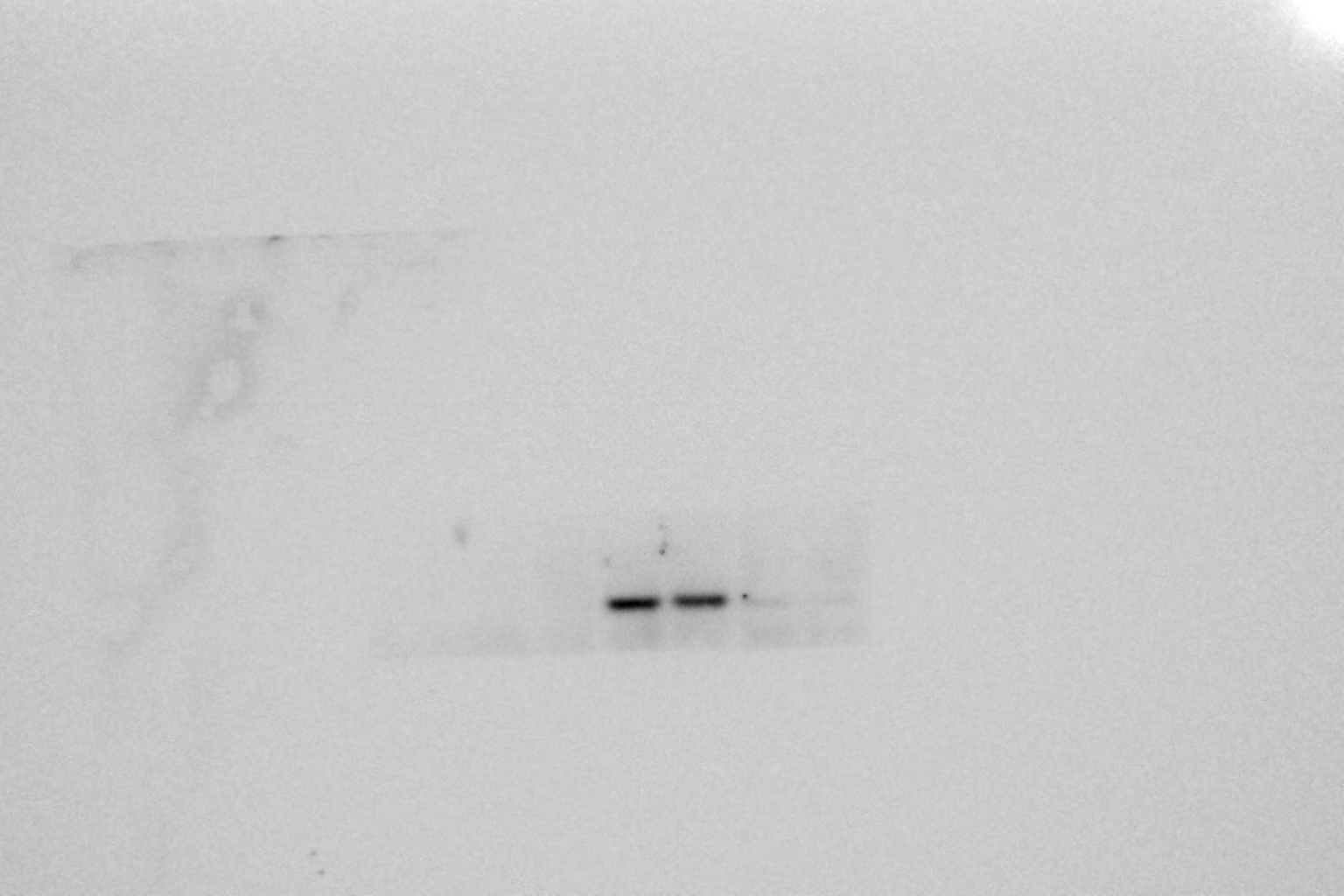

Supplement: Figure 6—source data 1. [file elife-88256-fig6-data1.zip › Figure 6-source data 1. Raw unedited blots for (Figure 6)/Figure 6B/Ac-p53.tif]

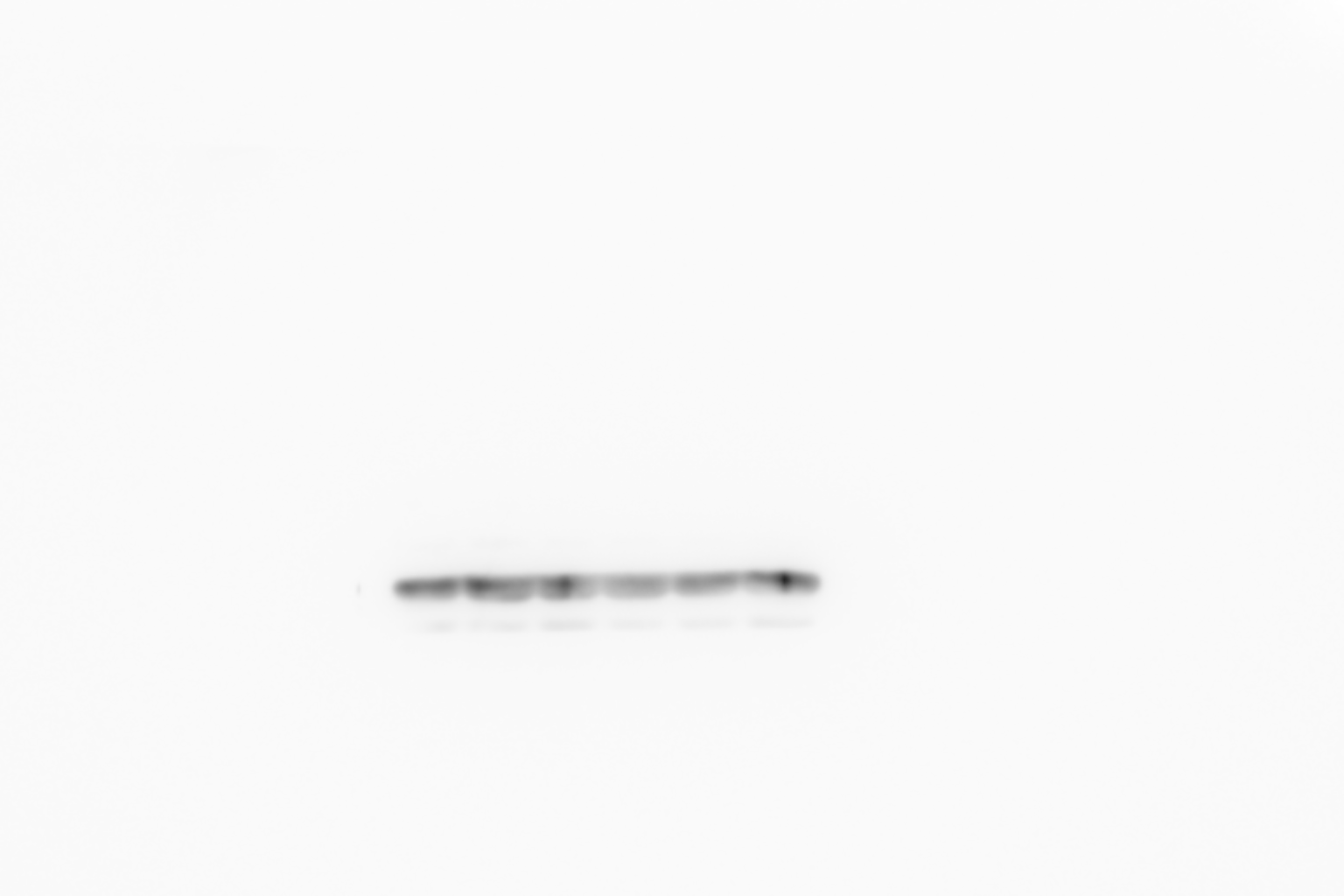

Supplement: Figure 6—source data 1. [file elife-88256-fig6-data1.zip › Figure 6-source data 1. Raw unedited blots for (Figure 6)/Figure 6B/GAPDH (Ac-p53).tif]

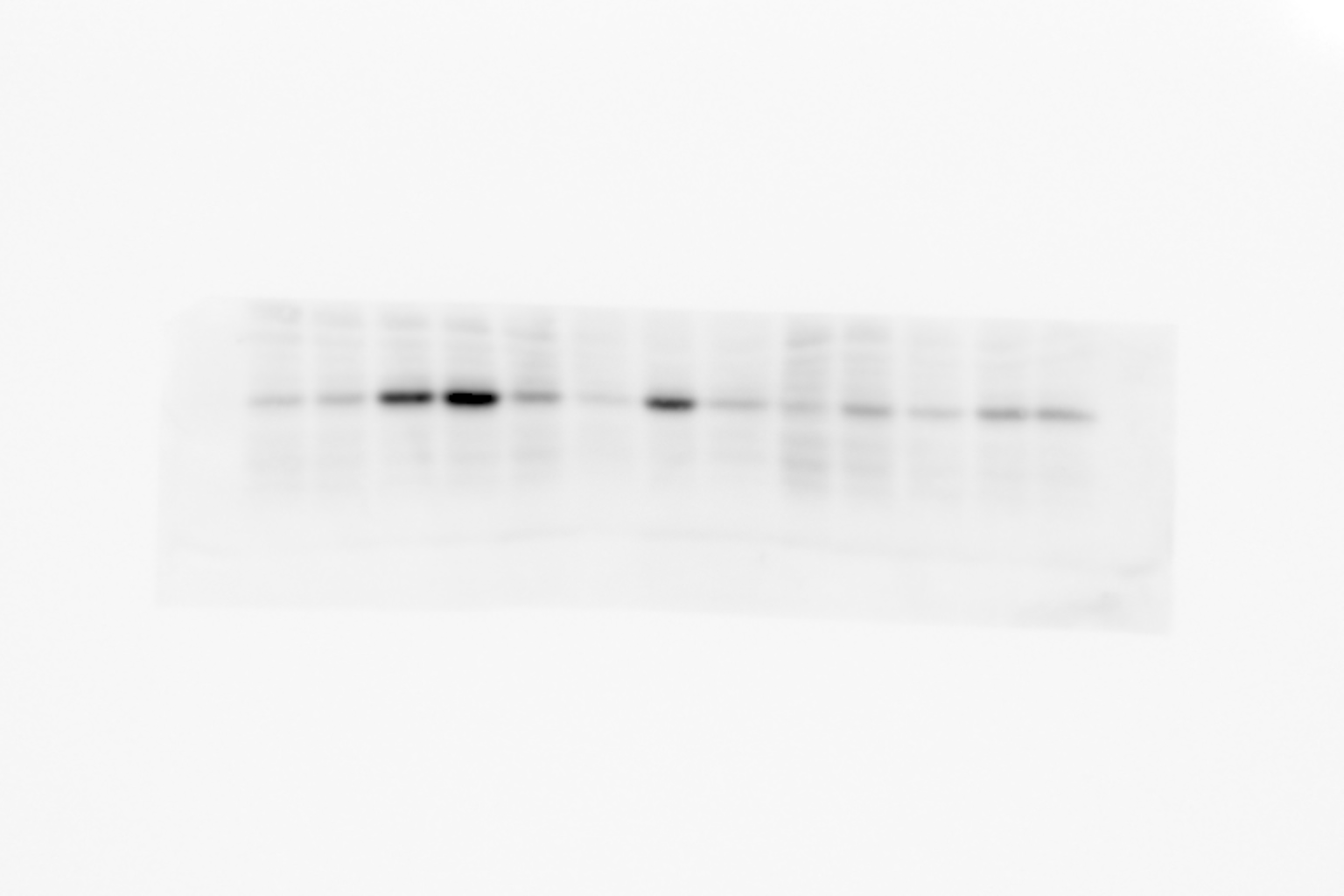

Supplement: Figure 6—source data 1. [file elife-88256-fig6-data1.zip › Figure 6-source data 1. Raw unedited blots for (Figure 6)/Figure 6B/p21.tif]

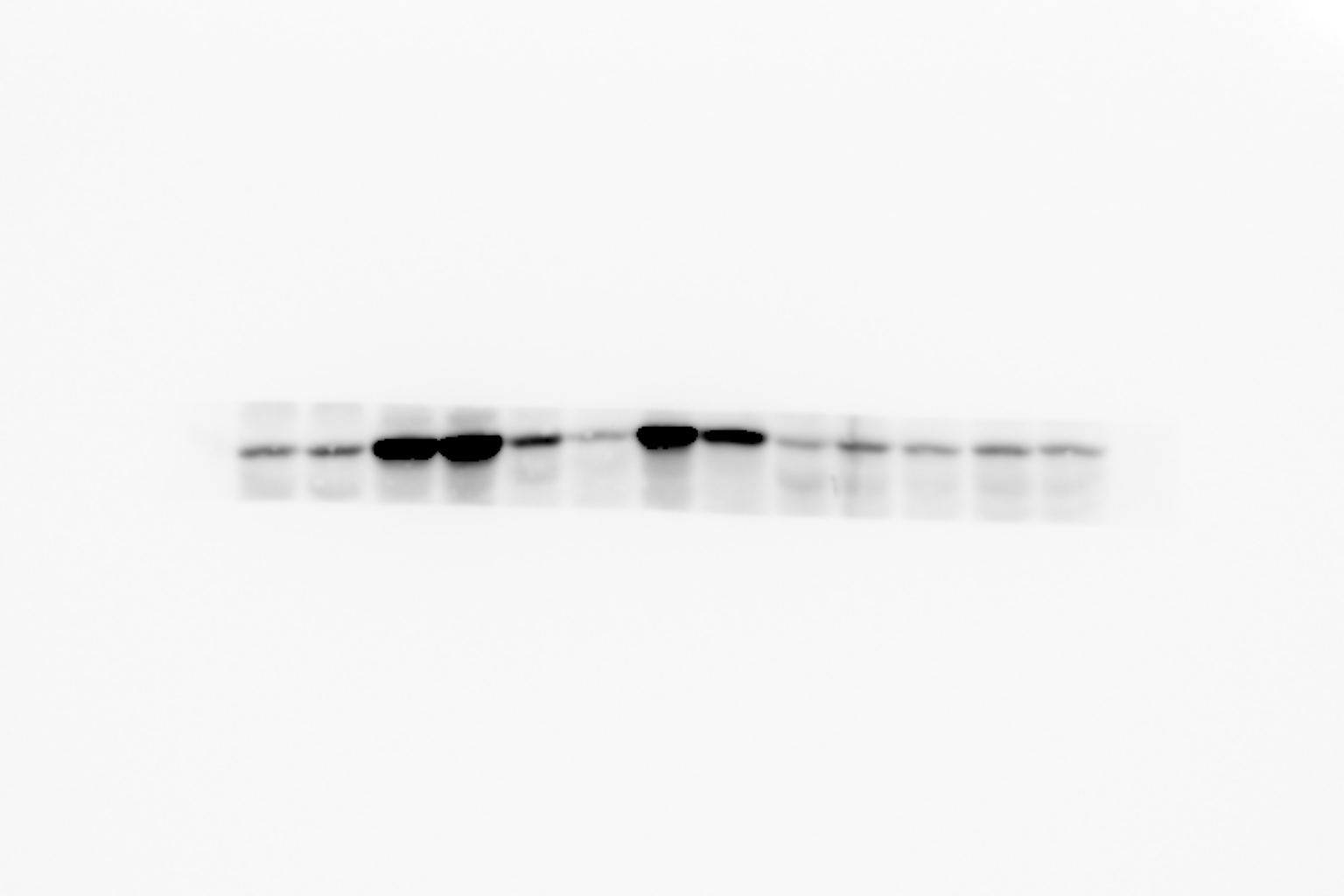

Supplement: Figure 6—source data 1. [file elife-88256-fig6-data1.zip › Figure 6-source data 1. Raw unedited blots for (Figure 6)/Figure 6B/p53.tif]

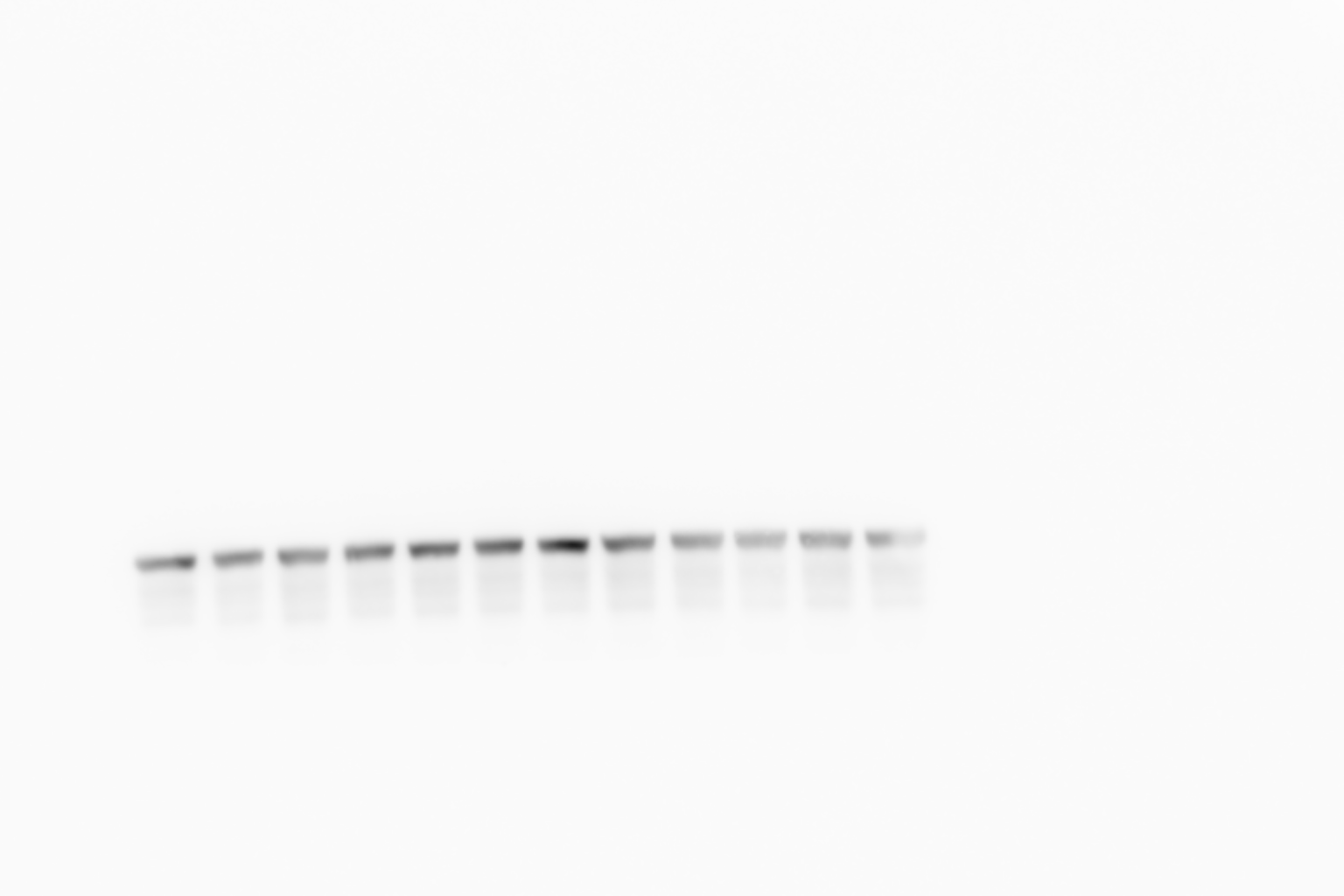

Supplement: Figure 6—source data 1. [file elife-88256-fig6-data1.zip › Figure 6-source data 1. Raw unedited blots for (Figure 6)/Figure 6B/TRIM28.tif]

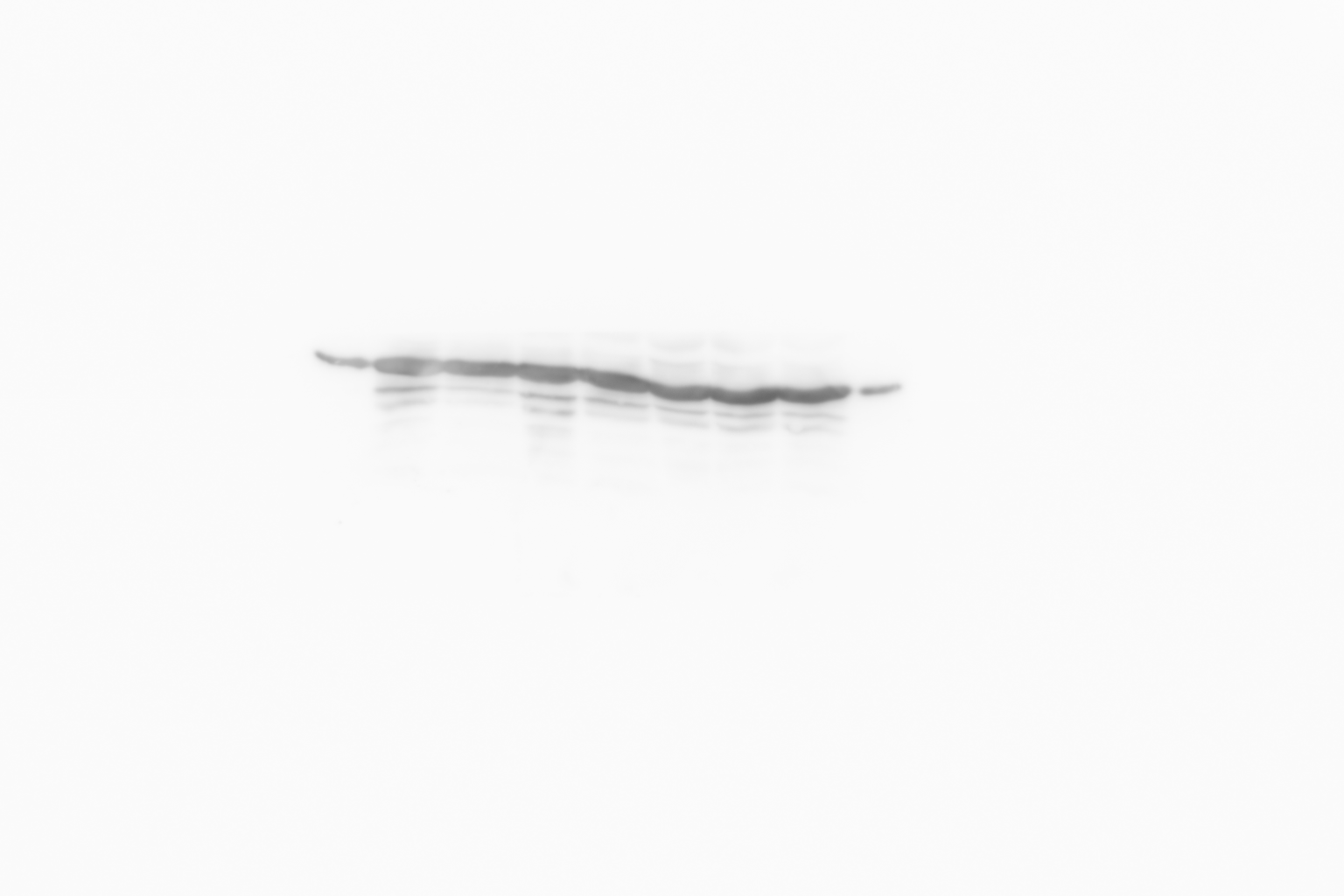

Supplement: Figure 6—source data 1. [file elife-88256-fig6-data1.zip › Figure 6-source data 1. Raw unedited blots for (Figure 6)/Figure 6F/GAPDH.tif]

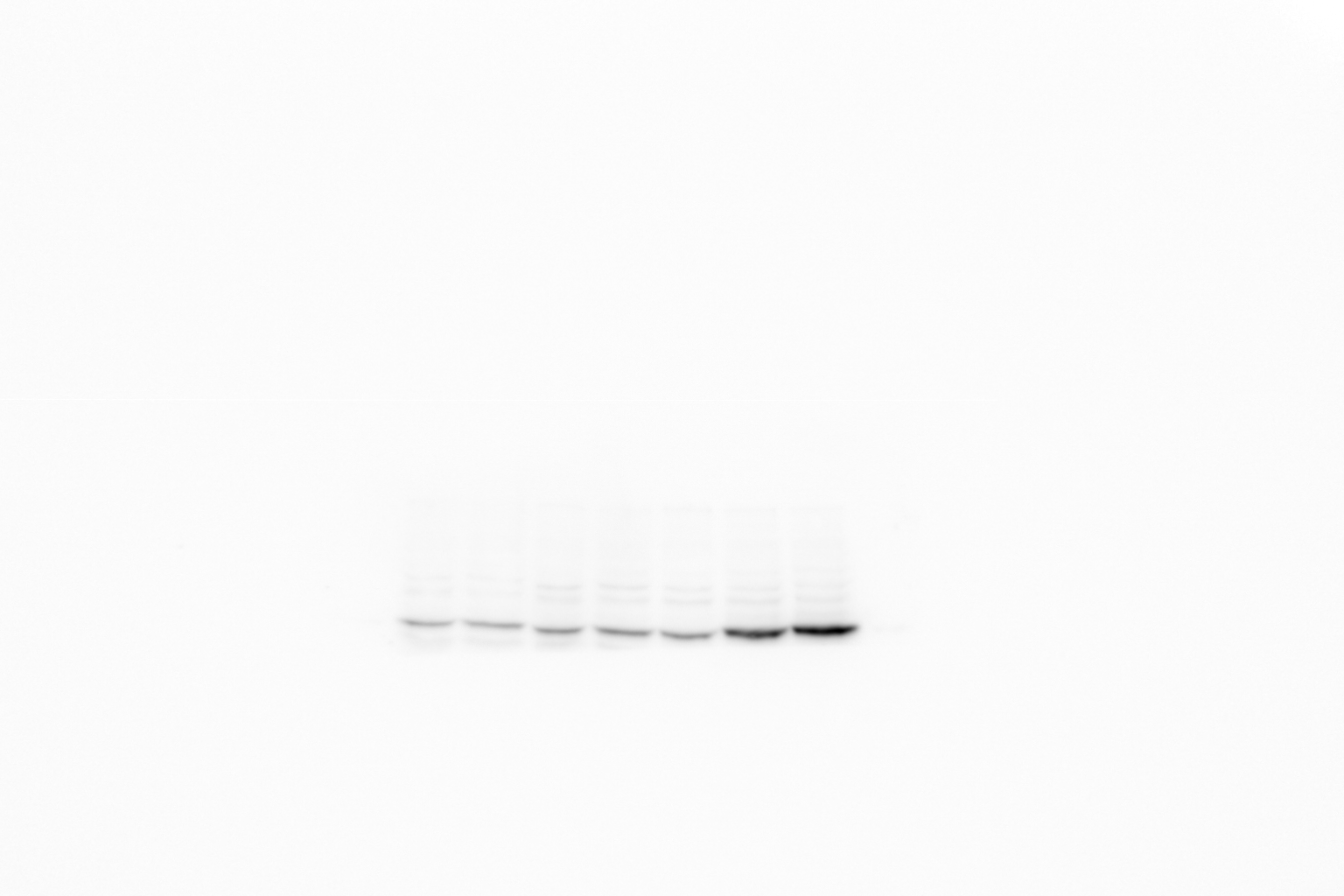

Supplement: Figure 6—source data 1. [file elife-88256-fig6-data1.zip › Figure 6-source data 1. Raw unedited blots for (Figure 6)/Figure 6F/p53.tif]

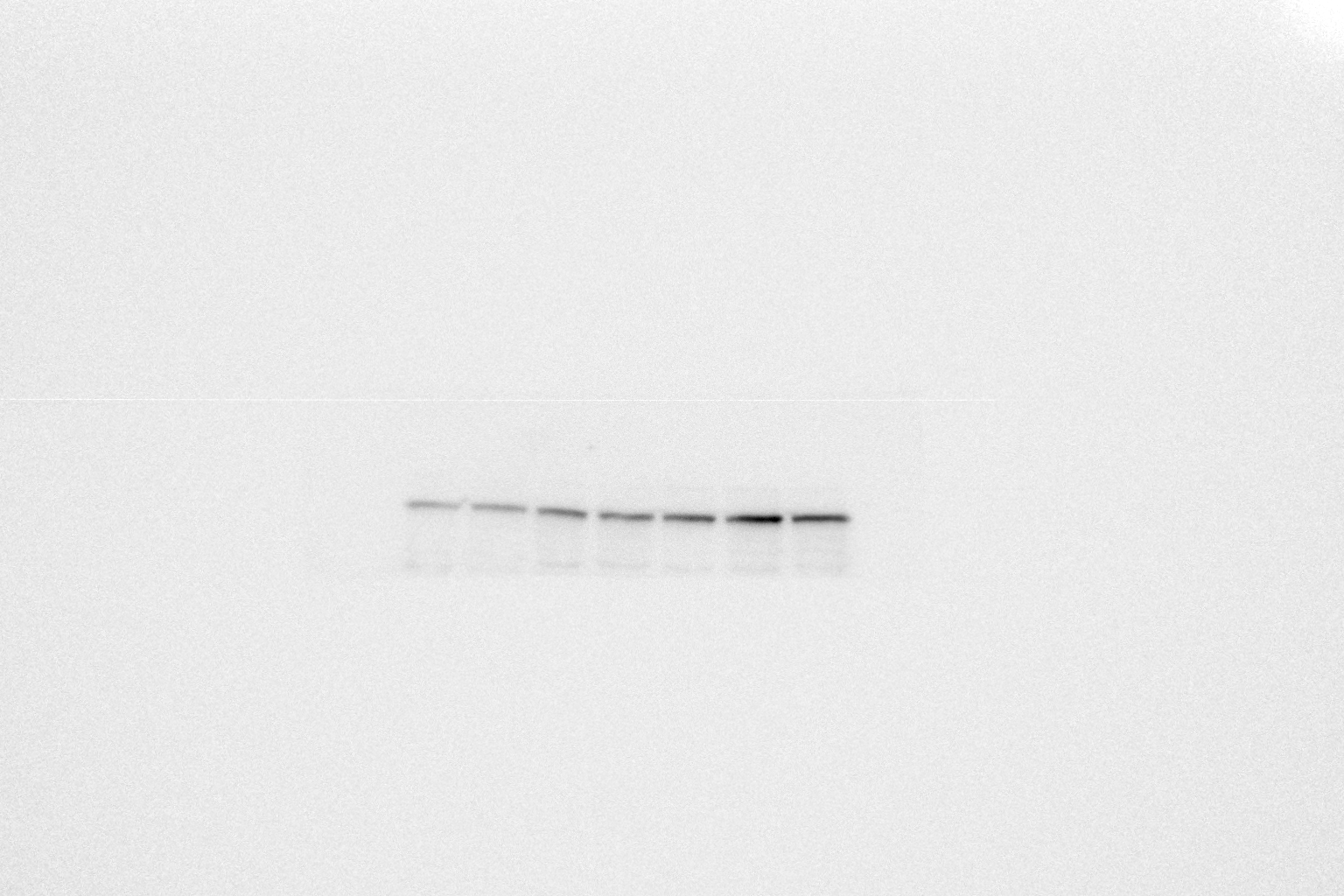

Supplement: Figure 6—source data 1. [file elife-88256-fig6-data1.zip › Figure 6-source data 1. Raw unedited blots for (Figure 6)/Figure 6F/TRIM28.tif]

**Figure 6B:**

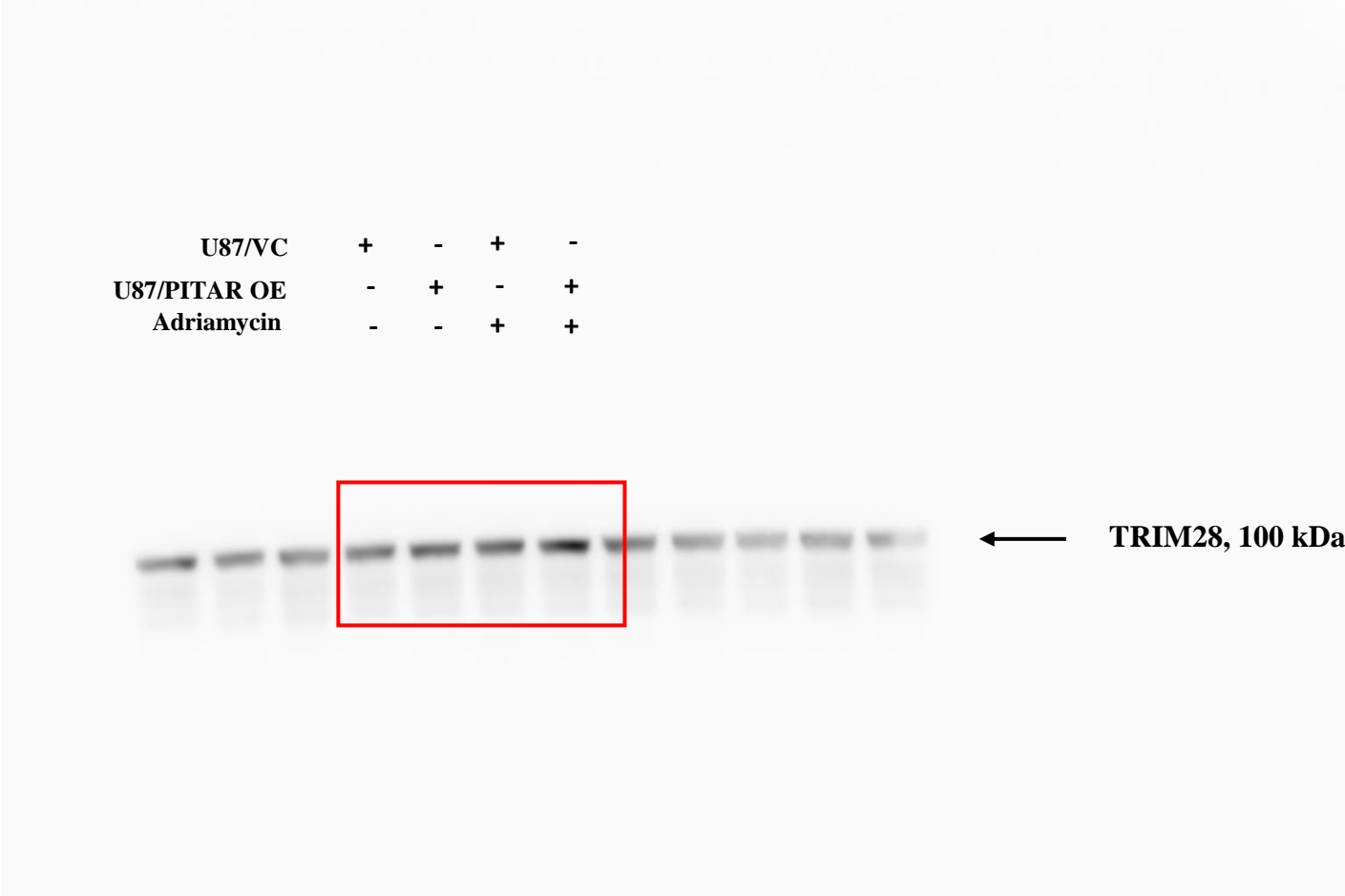

Figure 6B:

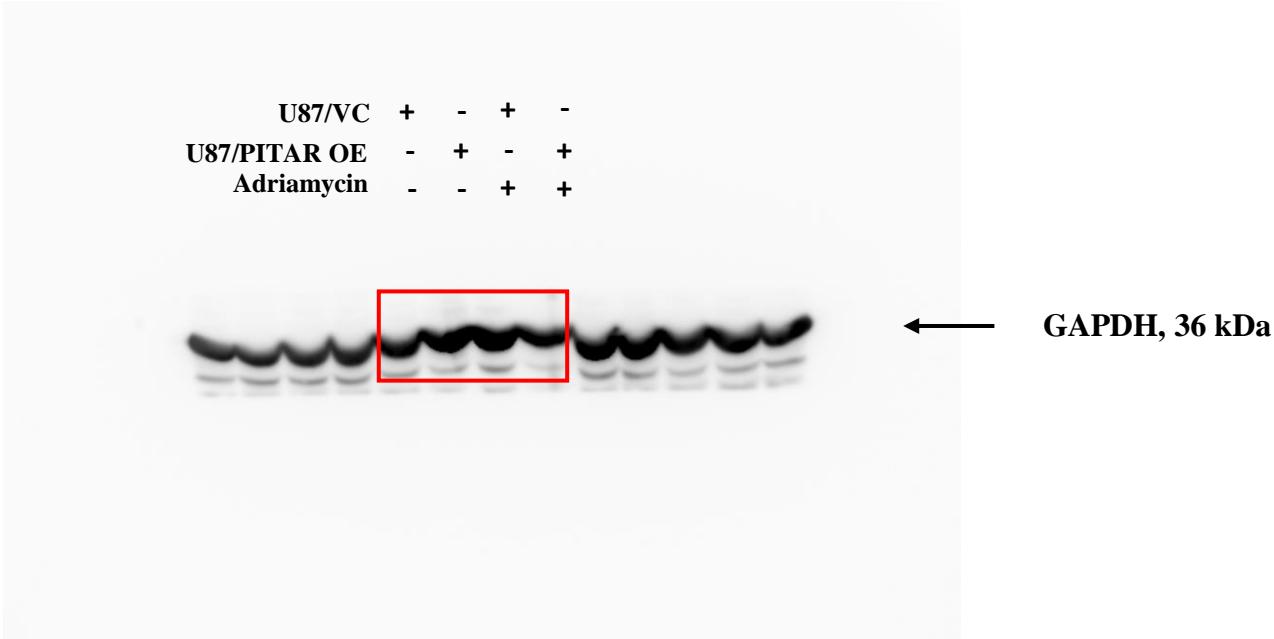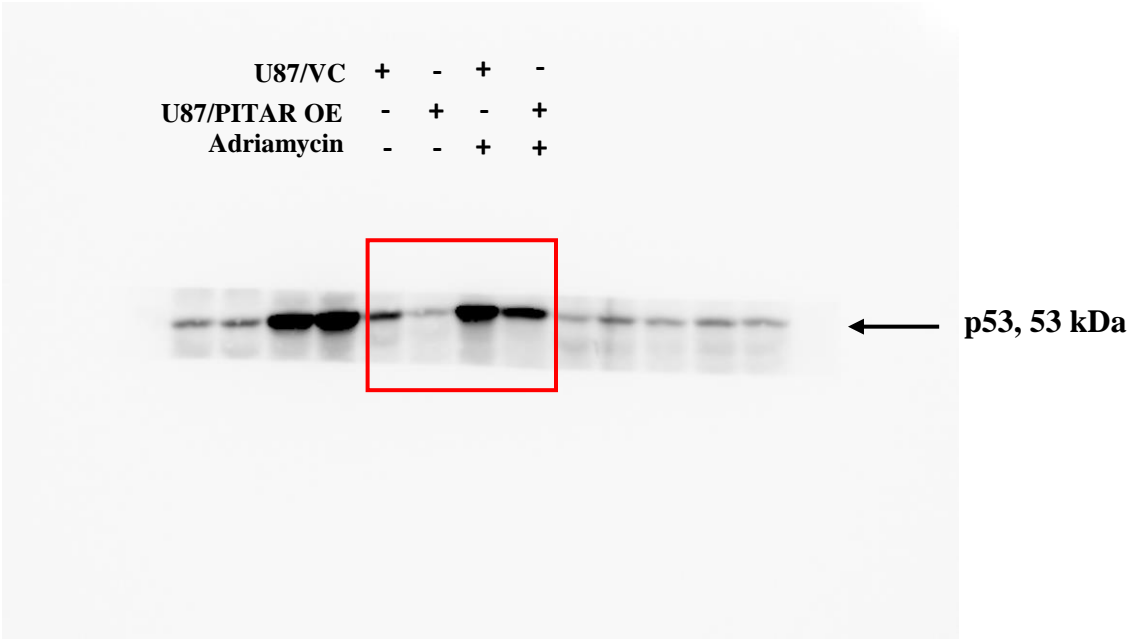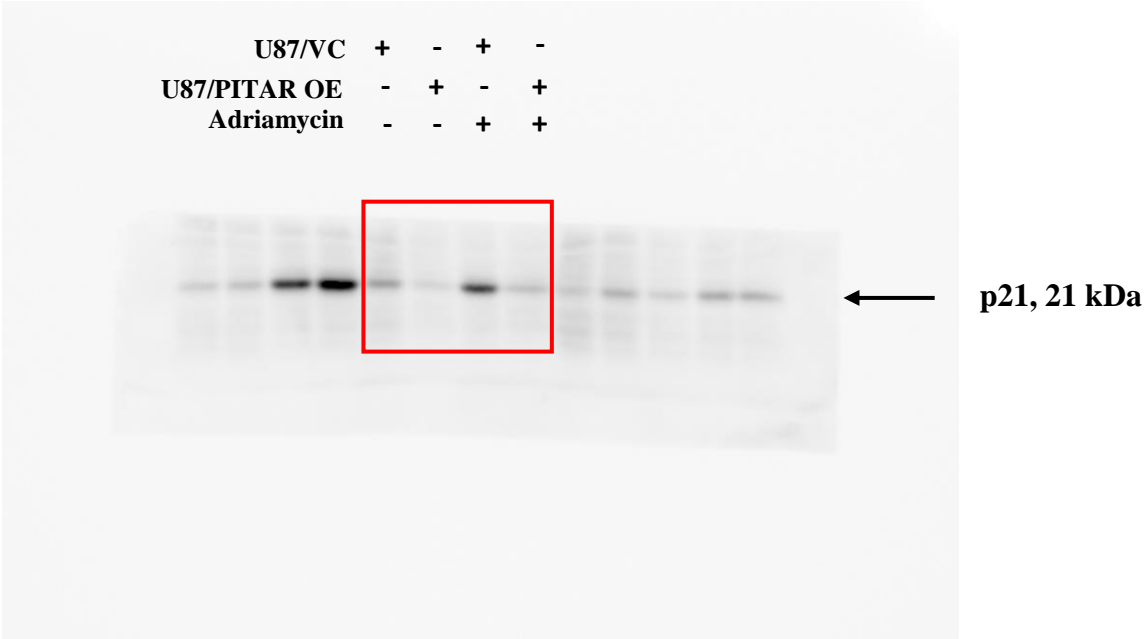

**Figure 6B:**

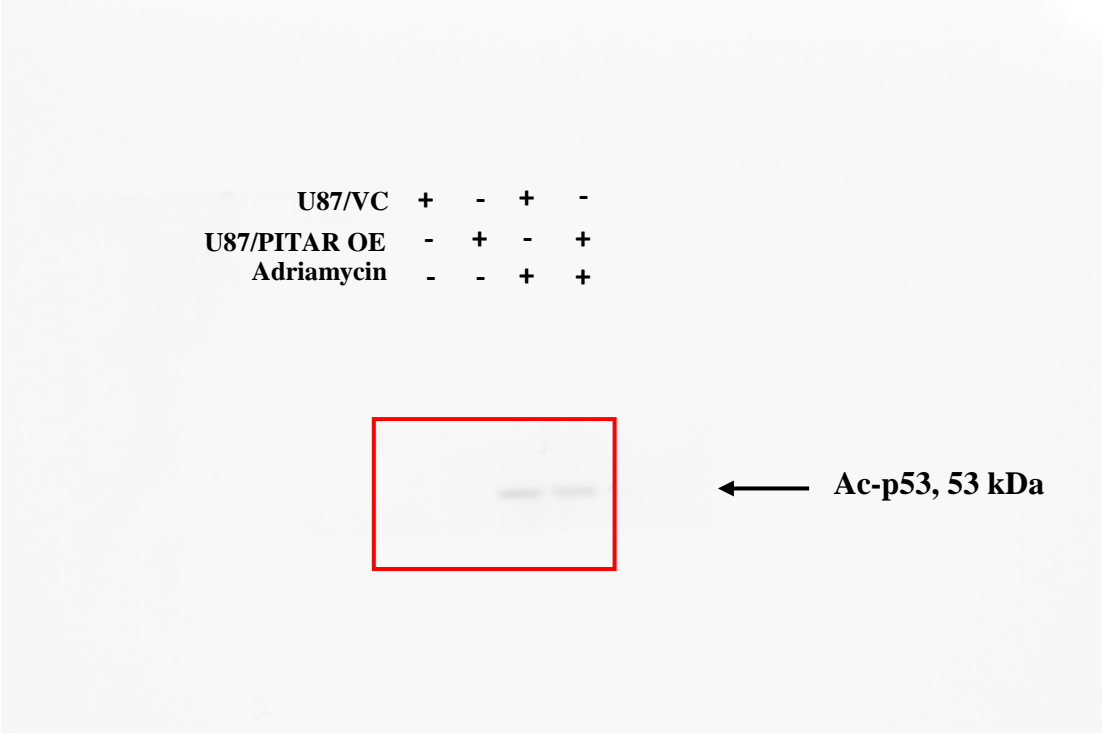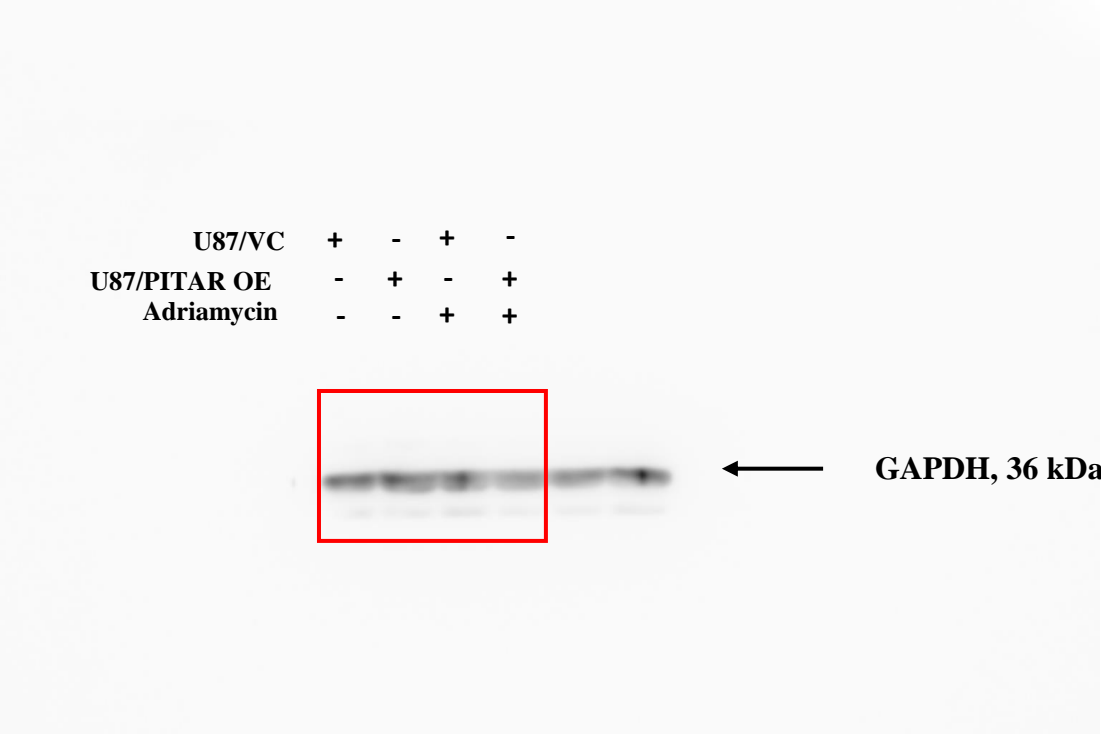

**Figure 6F:**

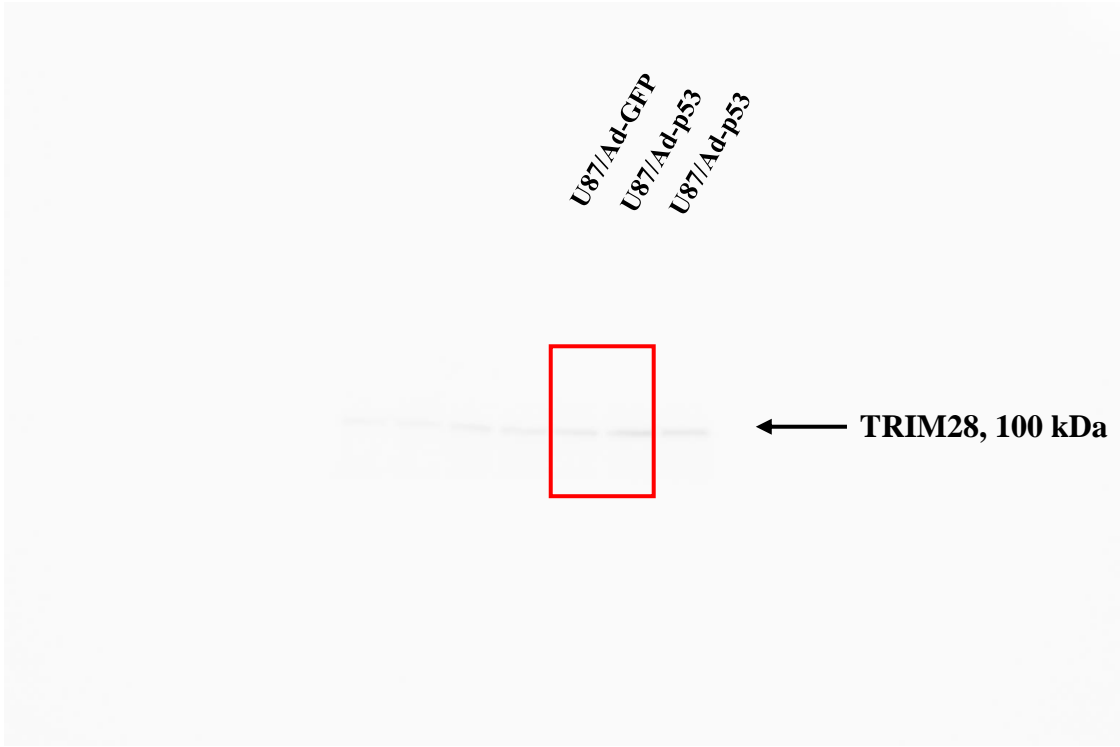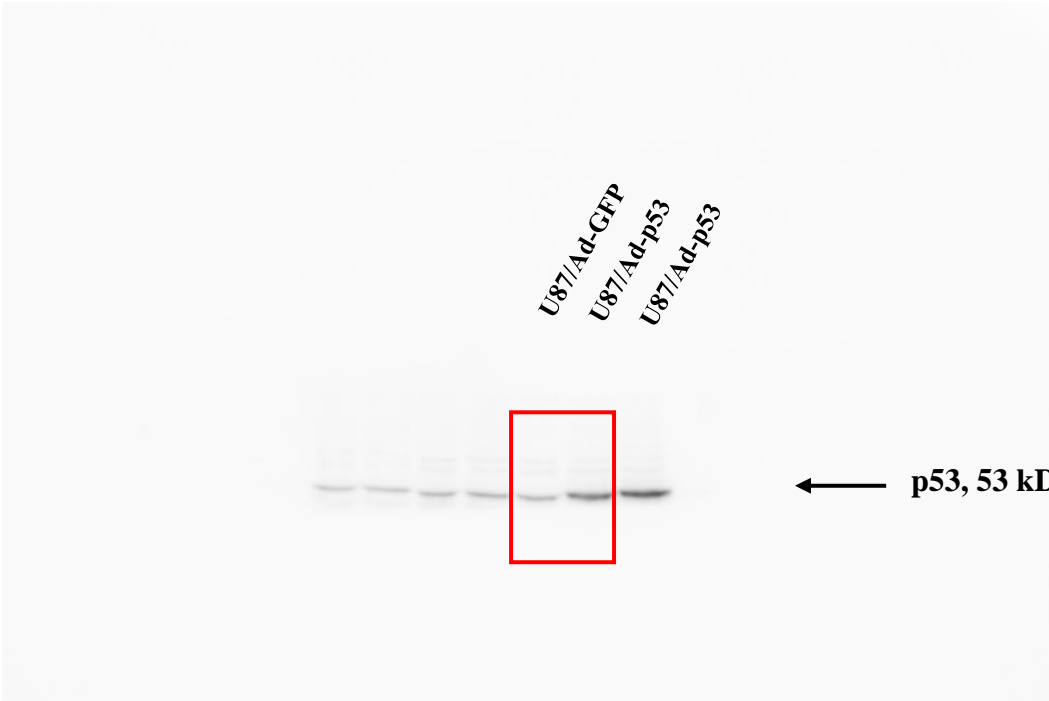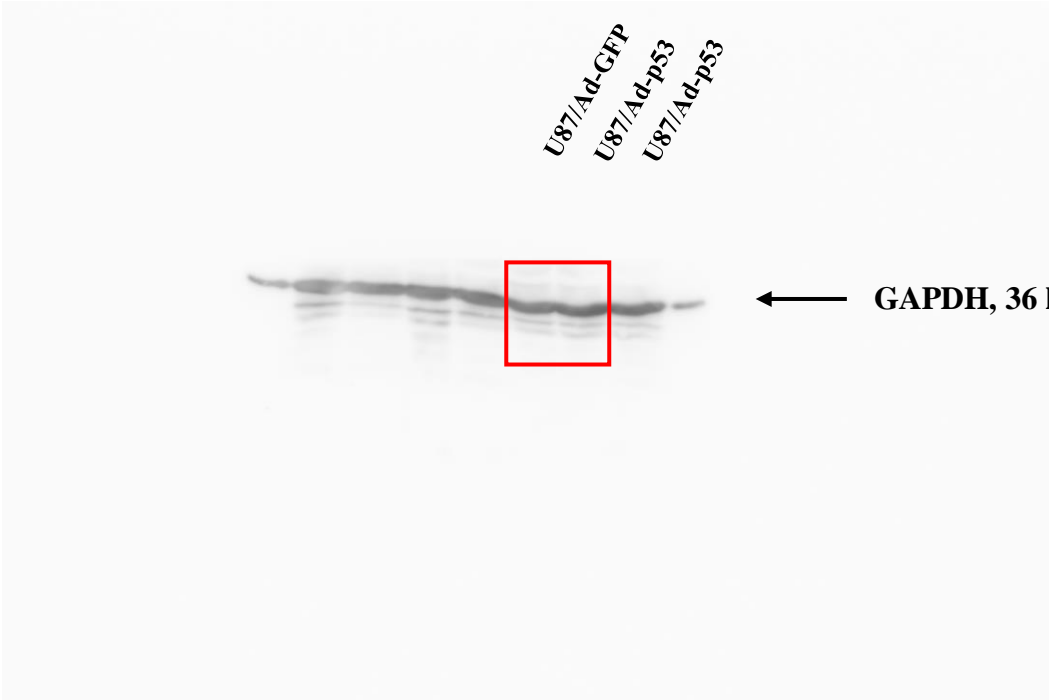

**Figure 6H:**

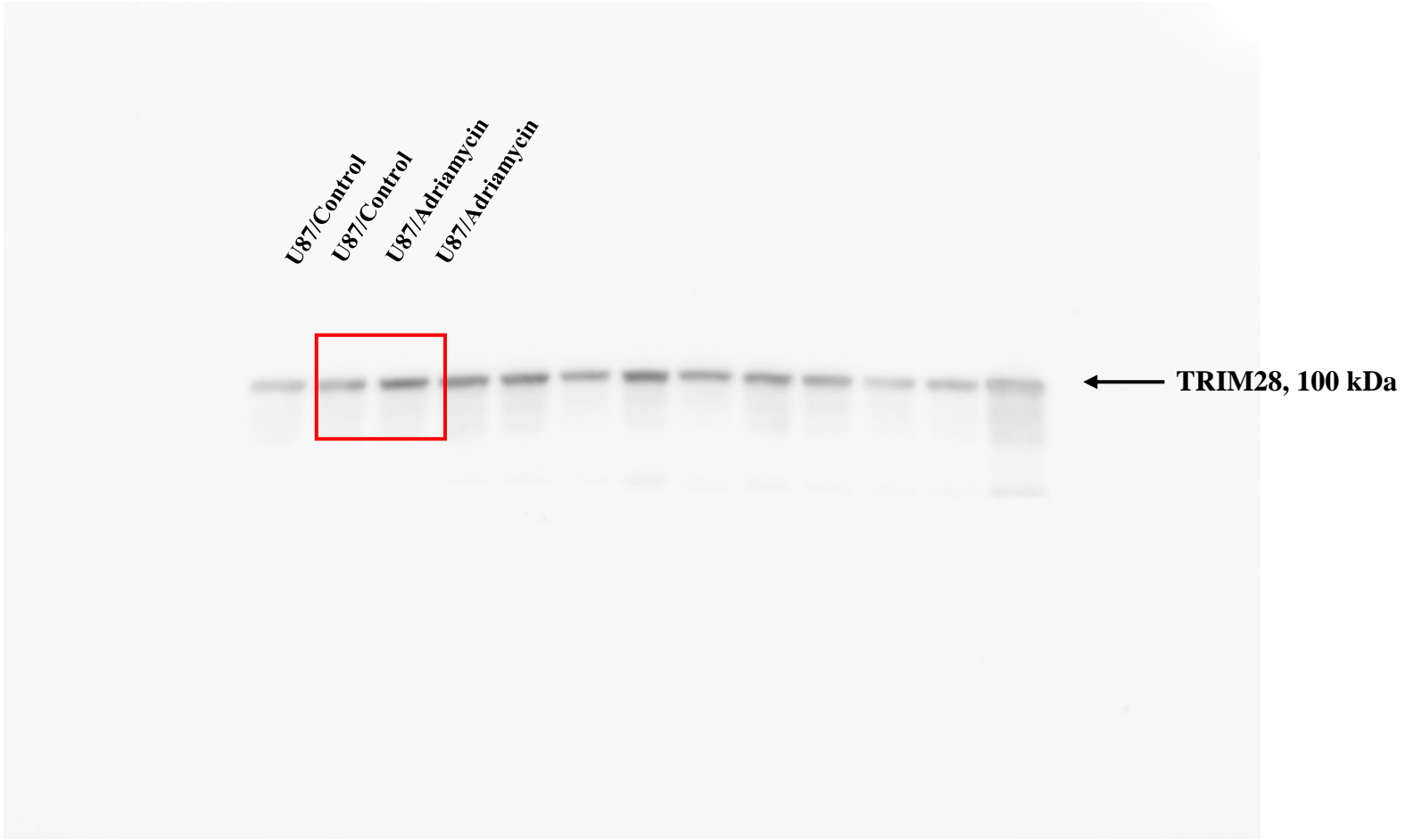

**Figure 6H:**

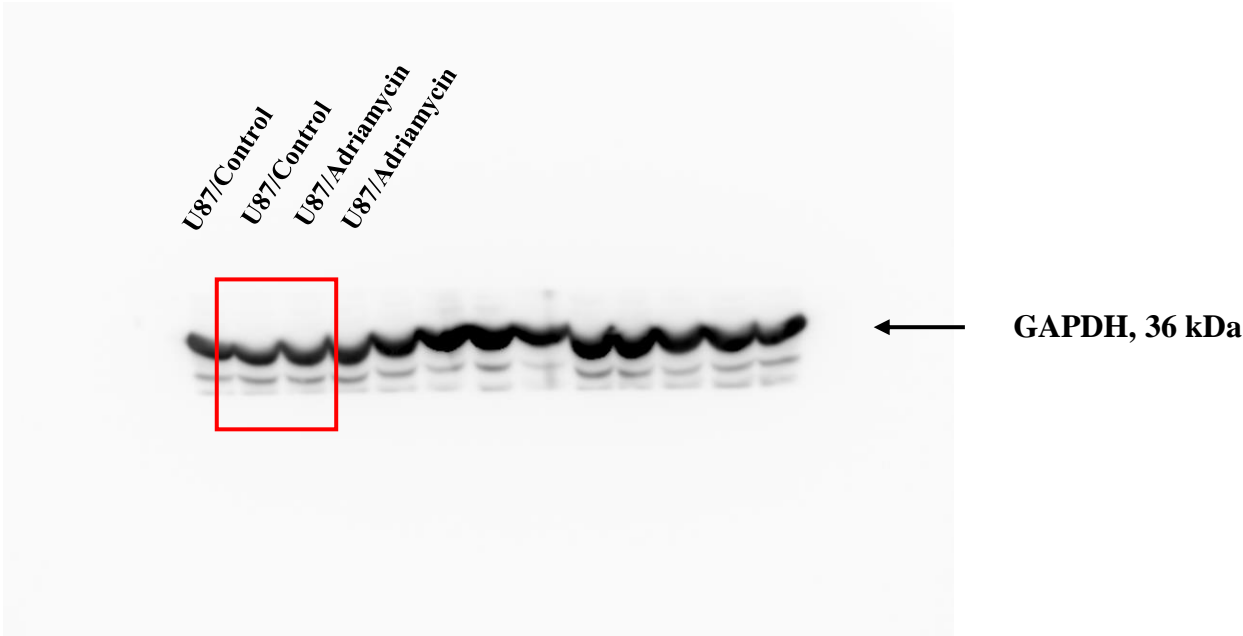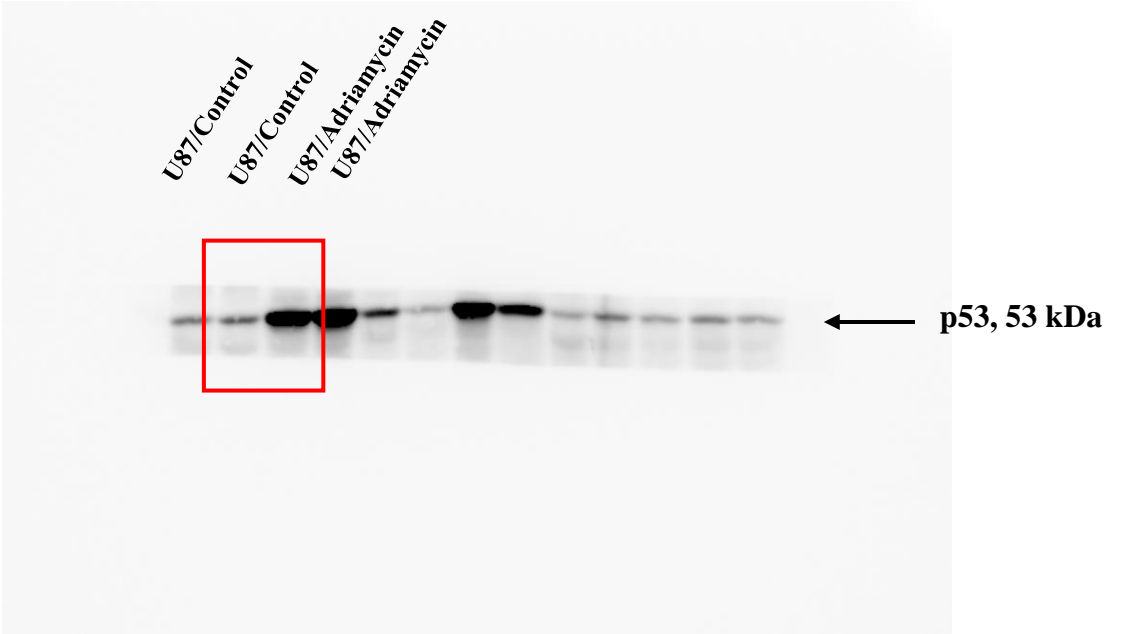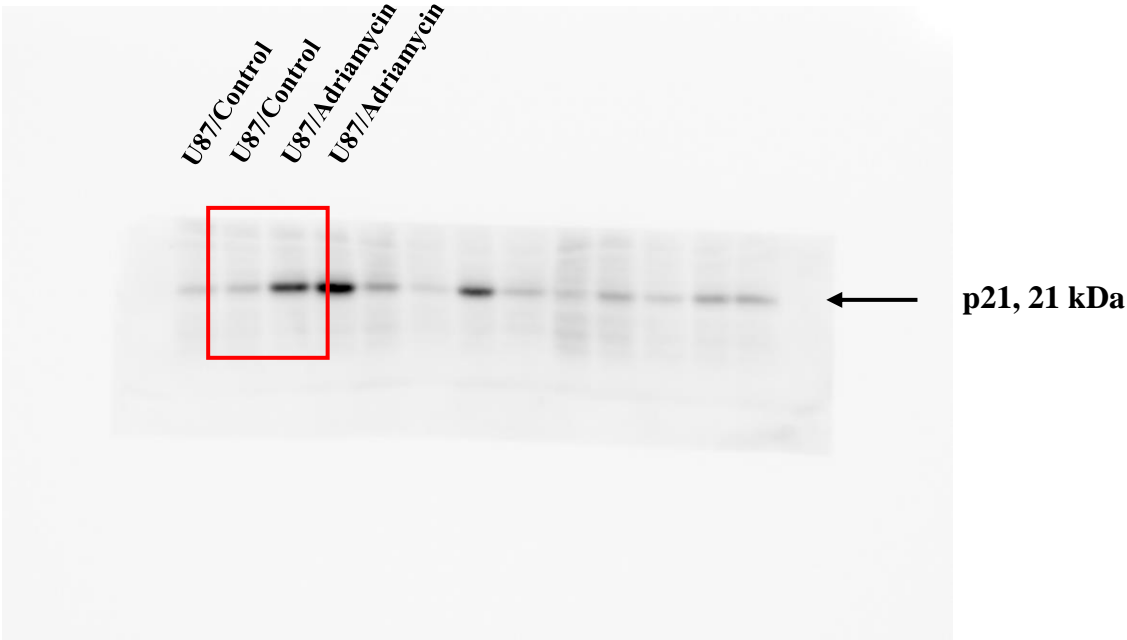

Supplement: Figure 6—source data 2. [file elife-88256-fig6-data2.pdf]

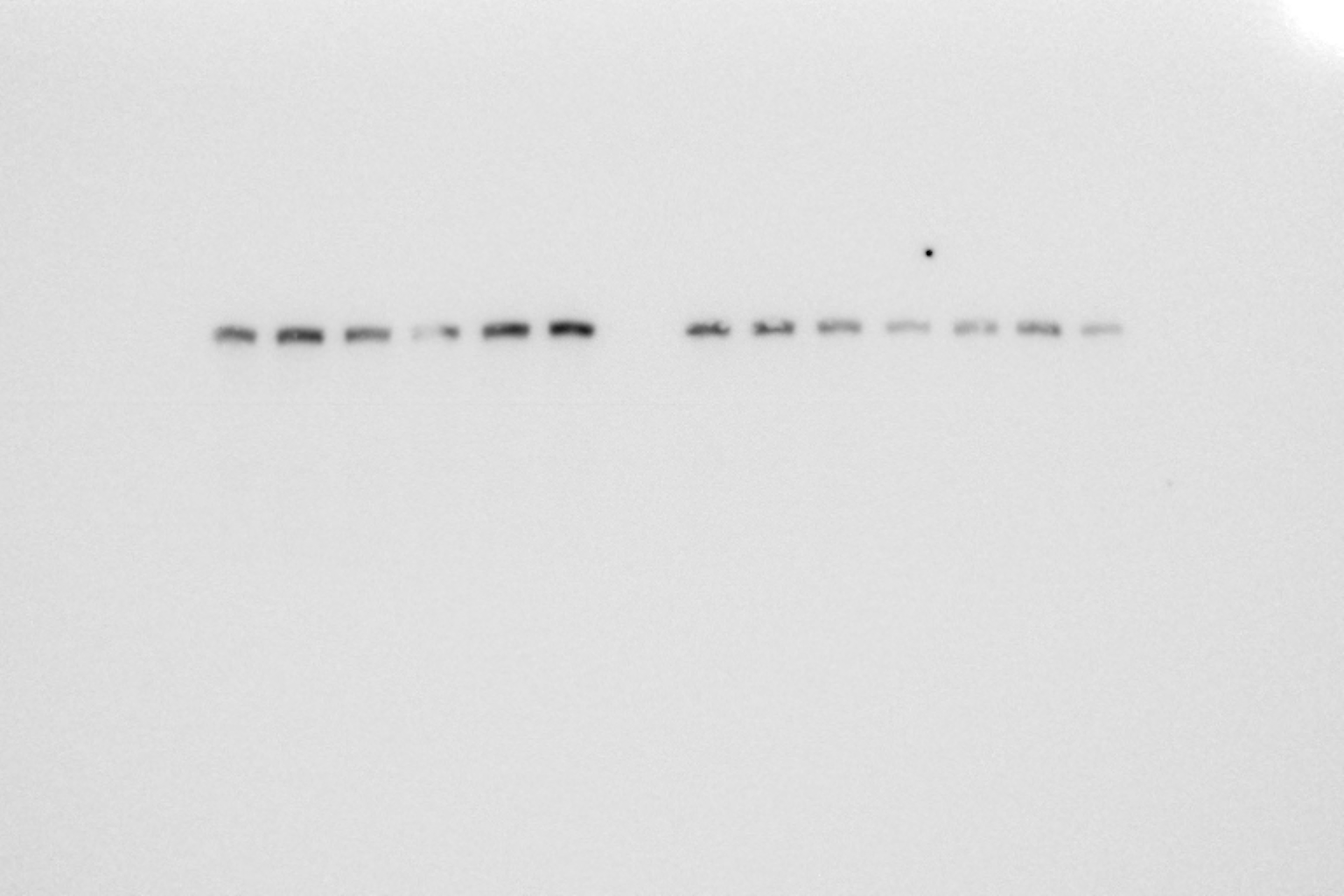

Supplement: Figure 6—figure supplement 1—source data 1. [file elife-88256-fig6-figsupp1-data1.zip › Figure 6-figure supplement 1-source data 1. Raw unedited blots for (Figure 6-figure supplement 1)/Figure 6C/TRIM28.tif]

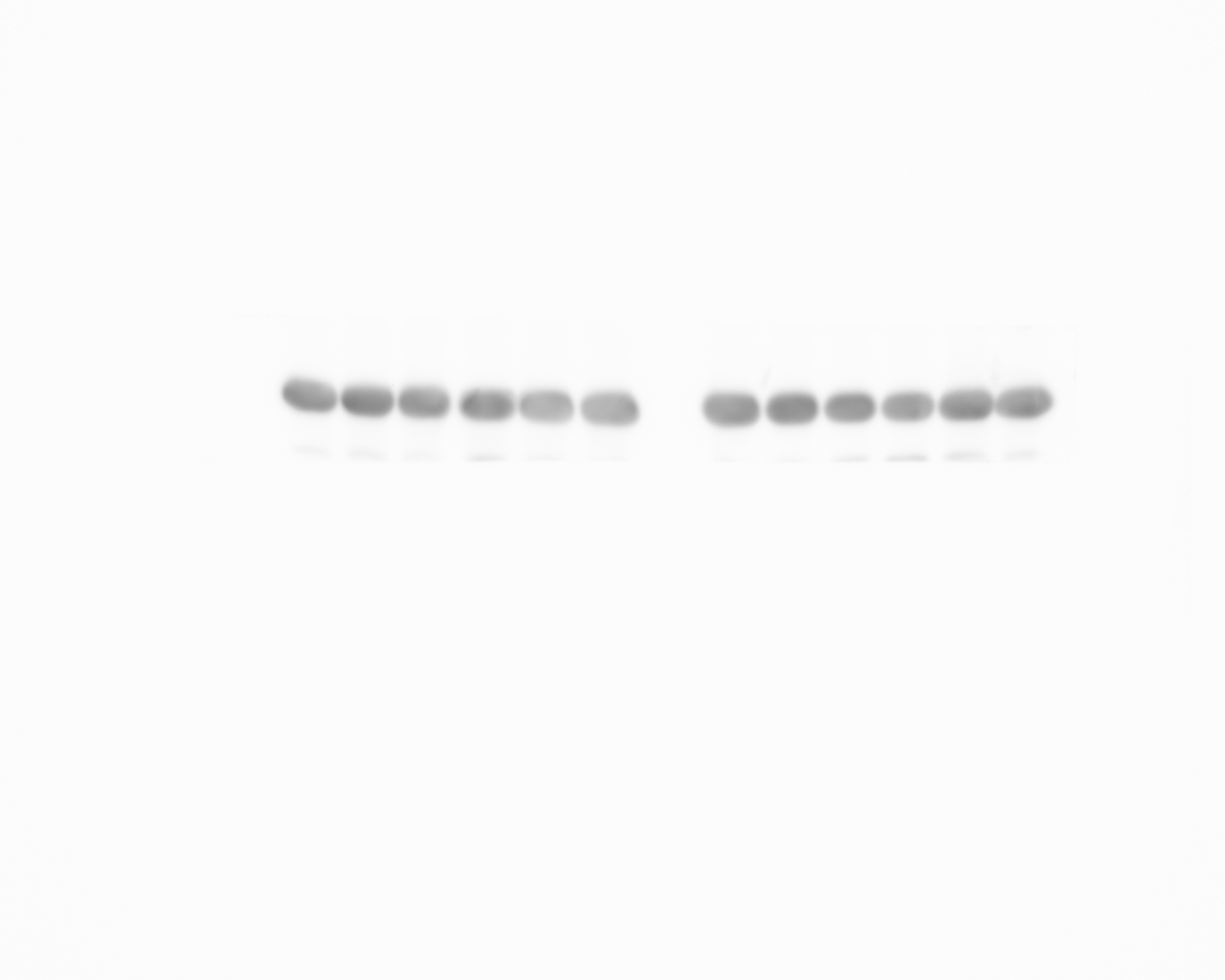

Supplement: Figure 6—figure supplement 1—source data 1. [file elife-88256-fig6-figsupp1-data1.zip › Figure 6-figure supplement 1-source data 1. Raw unedited blots for (Figure 6-figure supplement 1)/Figure 6E/GAPDH.tif]

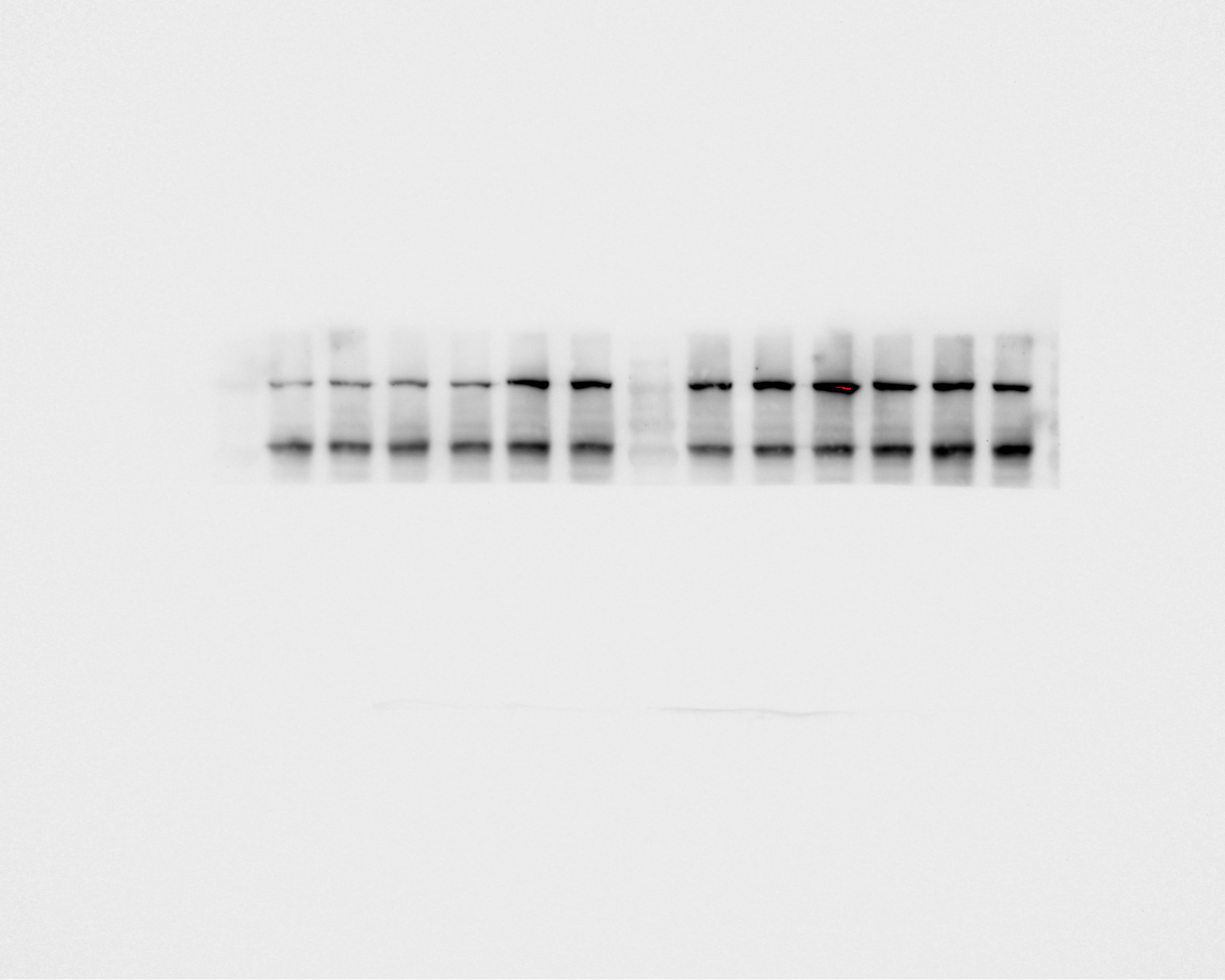

Supplement: Figure 6—figure supplement 1—source data 1. [file elife-88256-fig6-figsupp1-data1.zip › Figure 6-figure supplement 1-source data 1. Raw unedited blots for (Figure 6-figure supplement 1)/Figure 6E/Mdm2.tif]

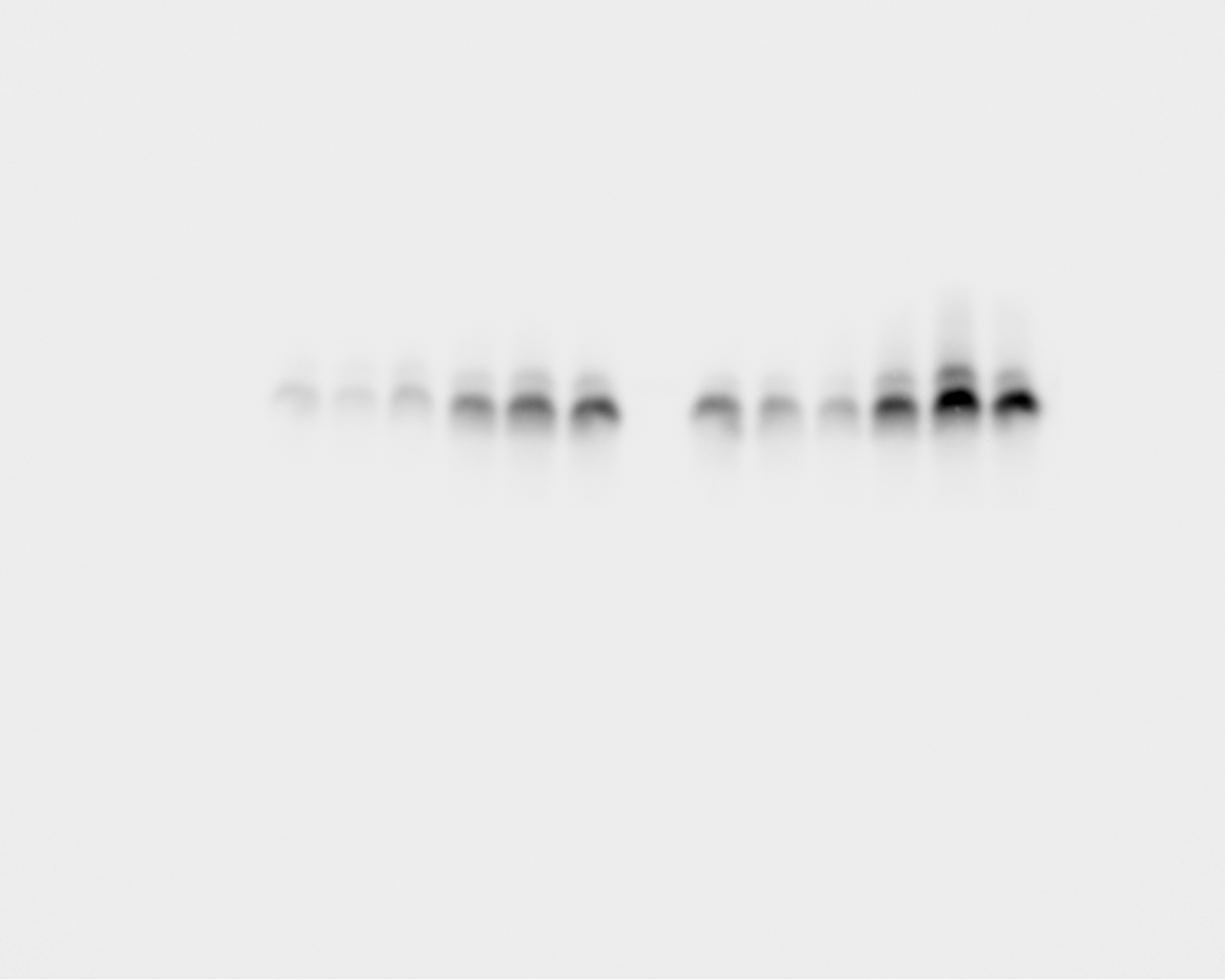

Supplement: Figure 6—figure supplement 1—source data 1. [file elife-88256-fig6-figsupp1-data1.zip › Figure 6-figure supplement 1-source data 1. Raw unedited blots for (Figure 6-figure supplement 1)/Figure 6E/p21.tif]

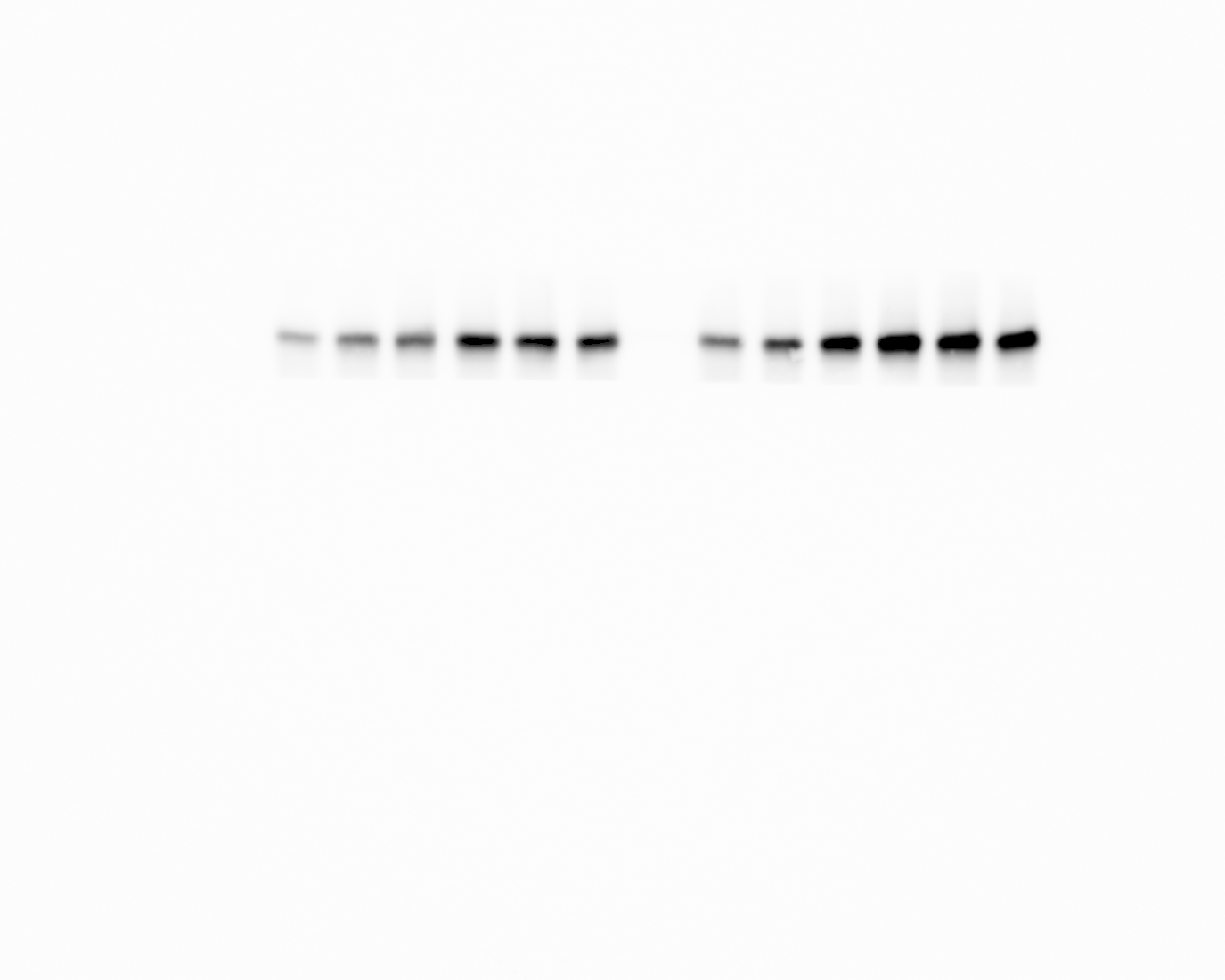

Supplement: Figure 6—figure supplement 1—source data 1. [file elife-88256-fig6-figsupp1-data1.zip › Figure 6-figure supplement 1-source data 1. Raw unedited blots for (Figure 6-figure supplement 1)/Figure 6E/p53.tif]
