# Supplementary material for: PITAR, a DNA damage-inducible cancer/testis long noncoding RNA, inactivates p53 by binding and stabilizing TRIM28 mRNA
Source: eLife. 2024 Sep 20;12:RP88256. doi: 10.7554/eLife.88256 (PMC11415074; doi:10.7554/eLife.88256)
Supplement: Figure 5—source data 2. [file elife-88256-fig5-data2.pdf]

Figure 5B:

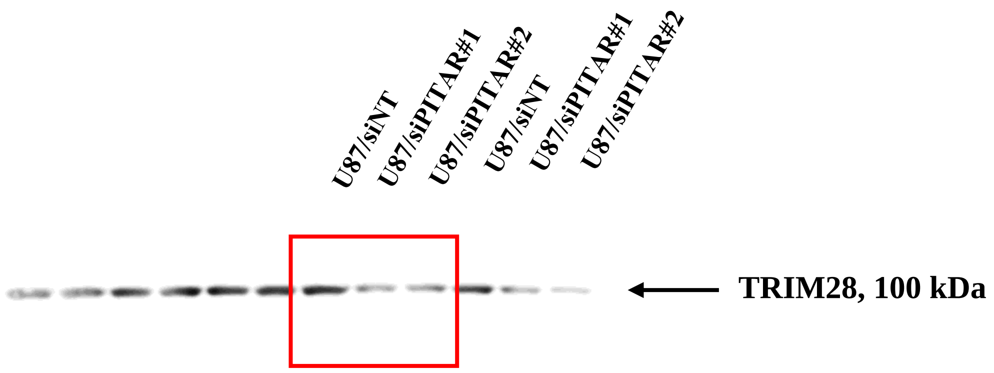
U87/siNT
U87/siPITAR#1
U87/siPITAR#2
U87/siNT
U87/siPITAR#1
U87/siPITAR#2
TRIM28, 100 kDa

Figure 5B:

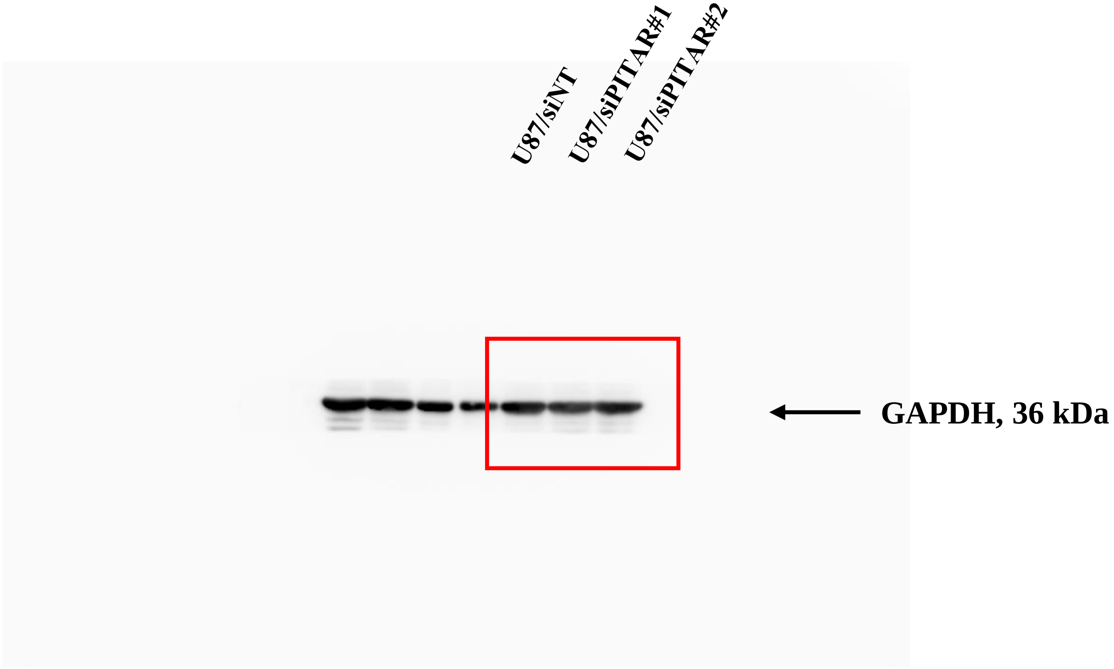
U87/siNT
U87/siPITAR#1
U87/siPITAR#2
GAPDH, 36 kDa

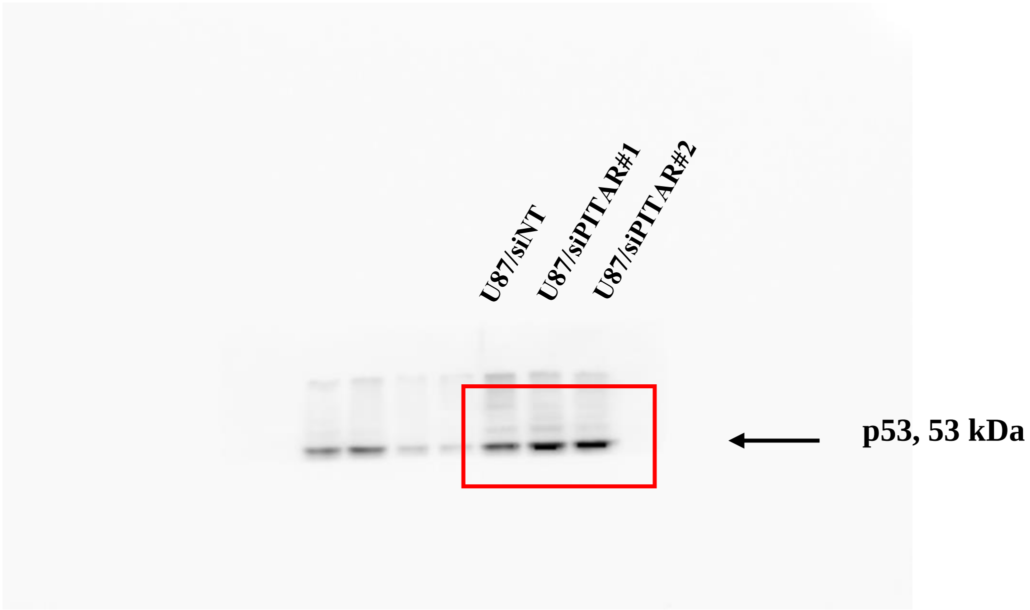
U87/siNT
U87/siPITAR#1
U87/siPITAR#2
p53, 53 kDa

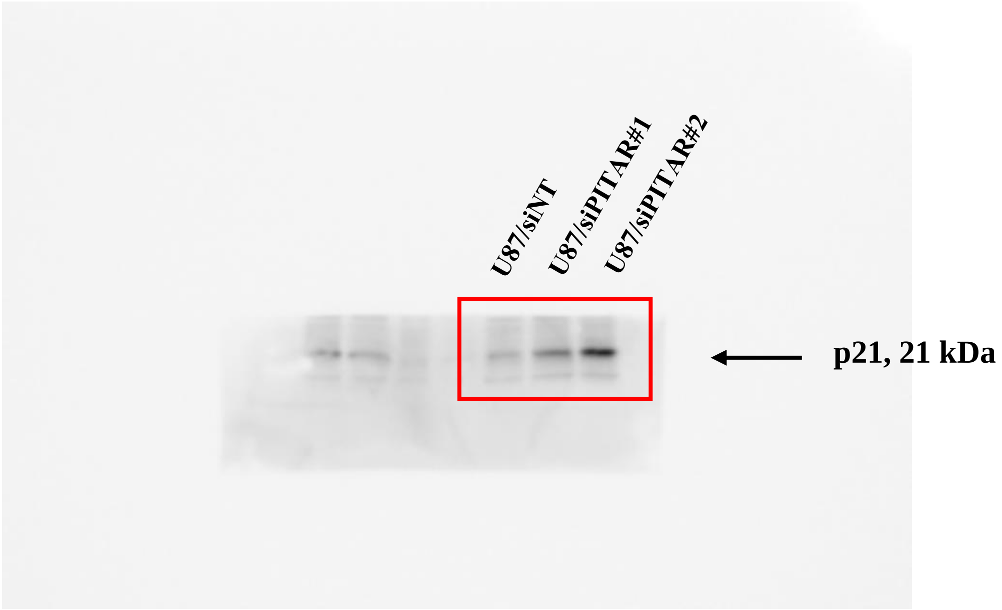
U87/siNT
U87/siPITAR#1
U87/siPITAR#2
p21, 21 kDa

Figure 5D:

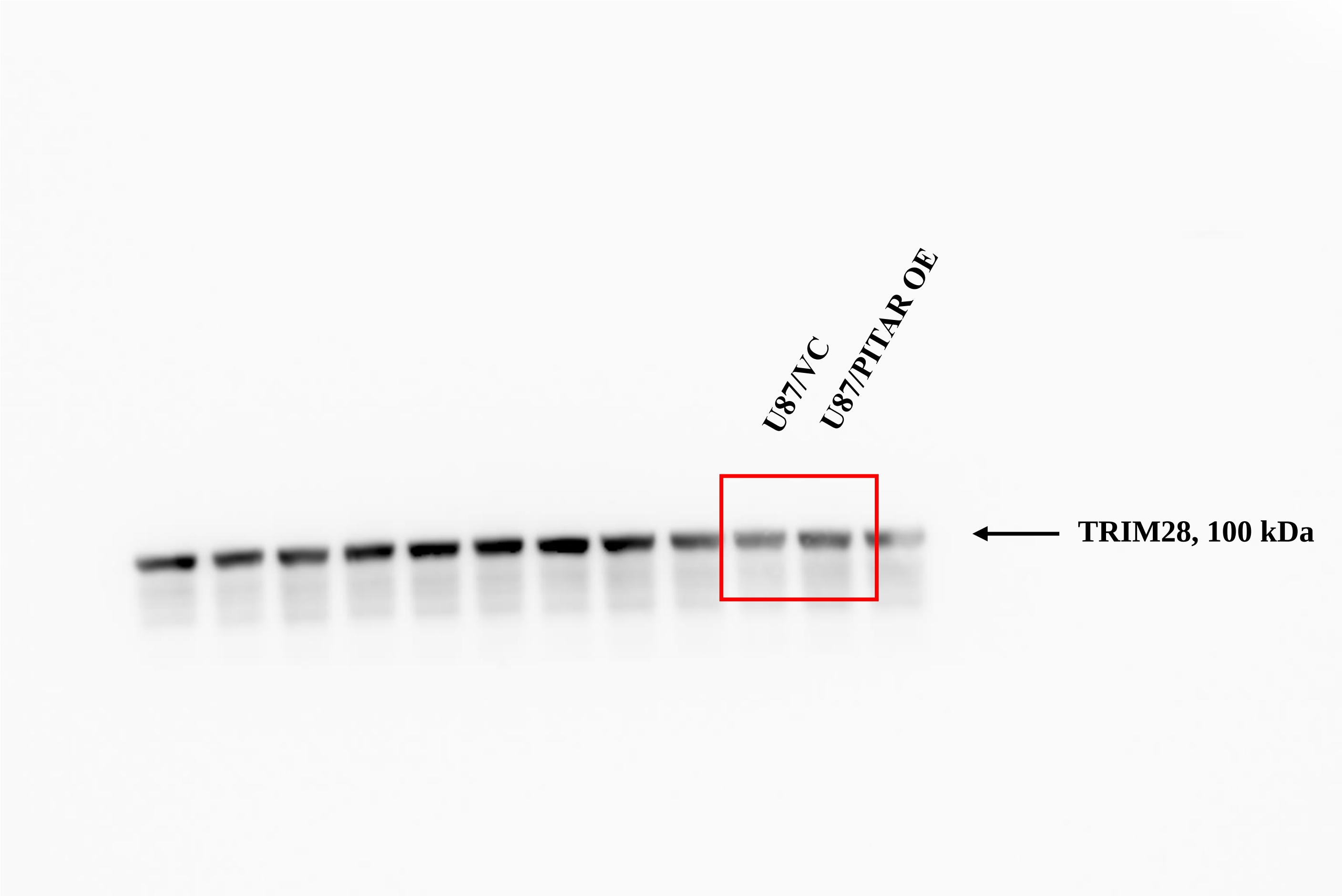

Figure 5D:

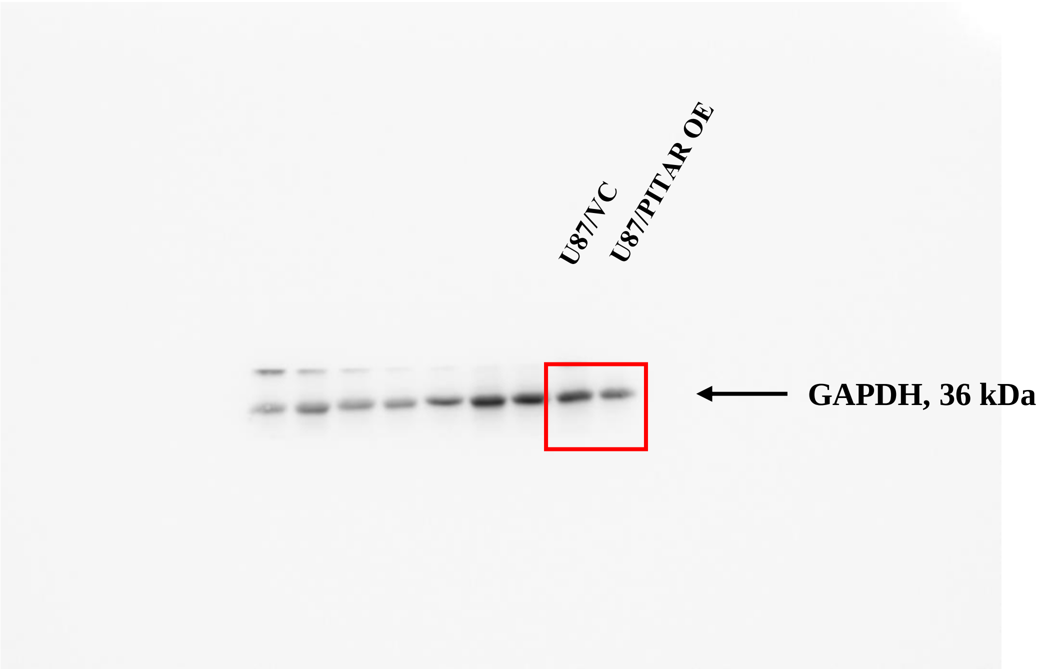
U87/VC
U87/PITAR OE
GAPDH, 36 kDa

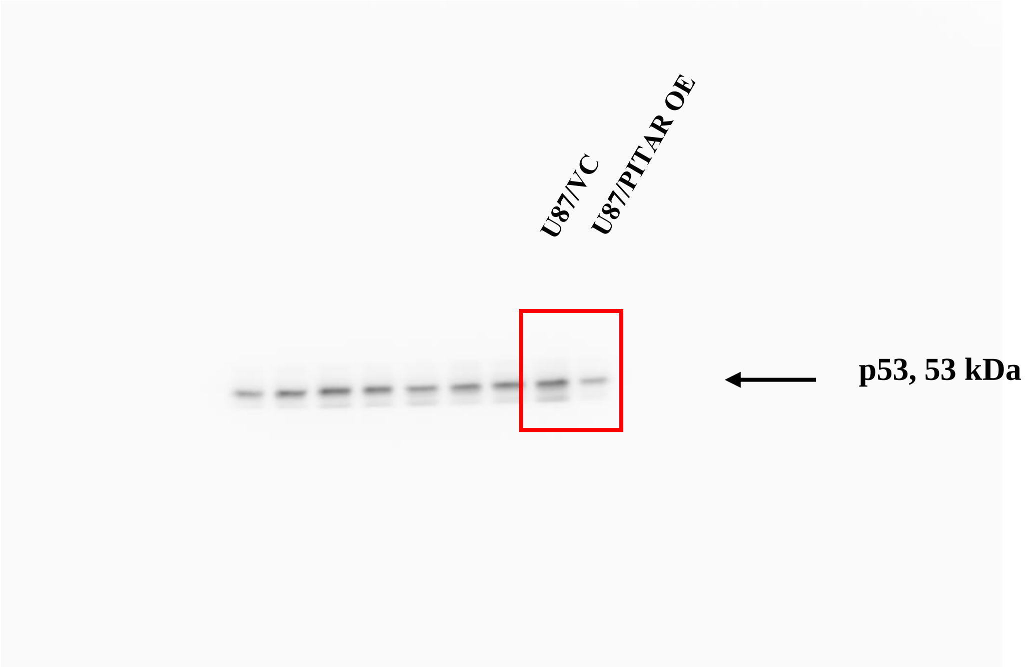
U87/VC
U87/PITAR OE
p53, 53 kDa

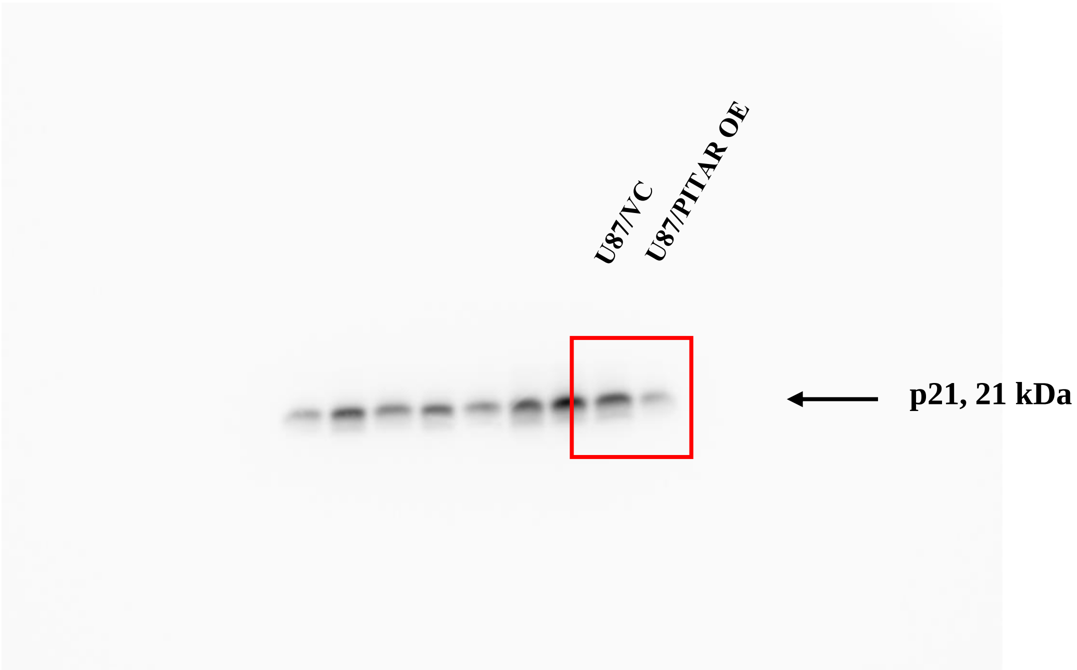
U87/VC
U87/PITAR OE
p21, 21 kDa

Figure 5E:

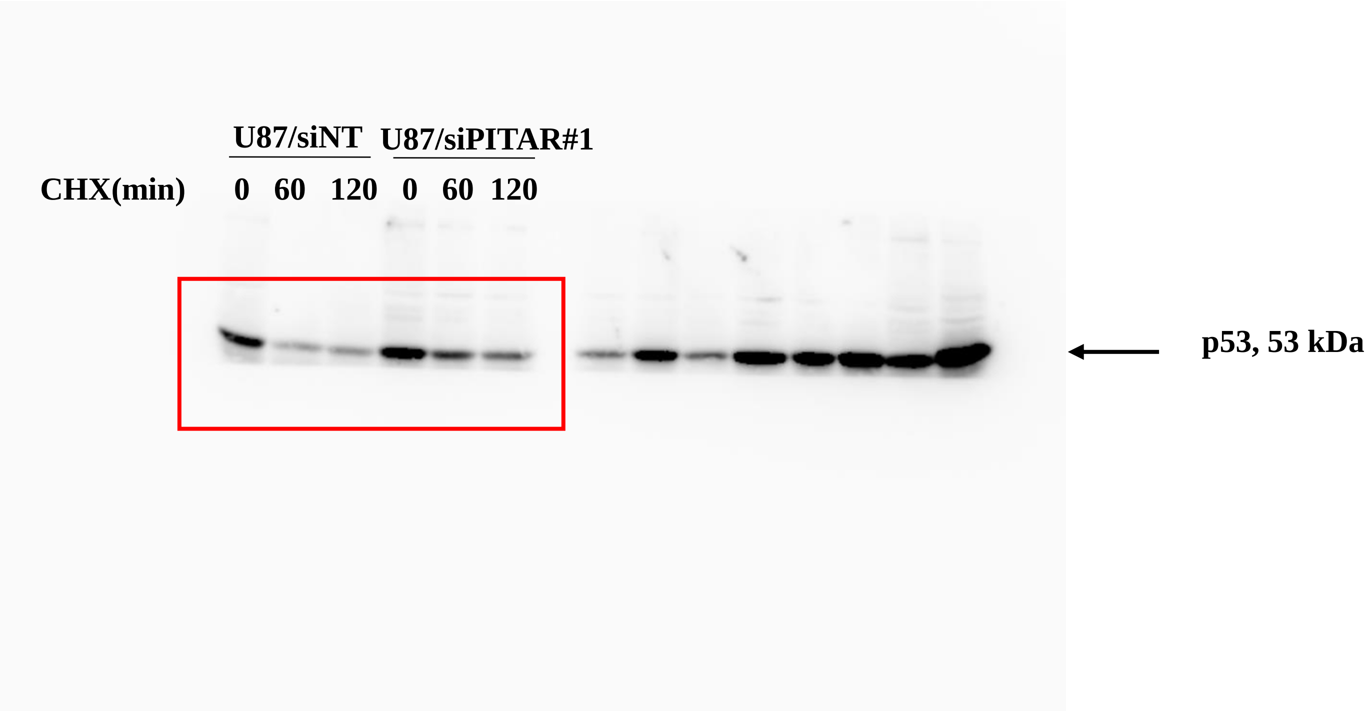

Figure 5E:

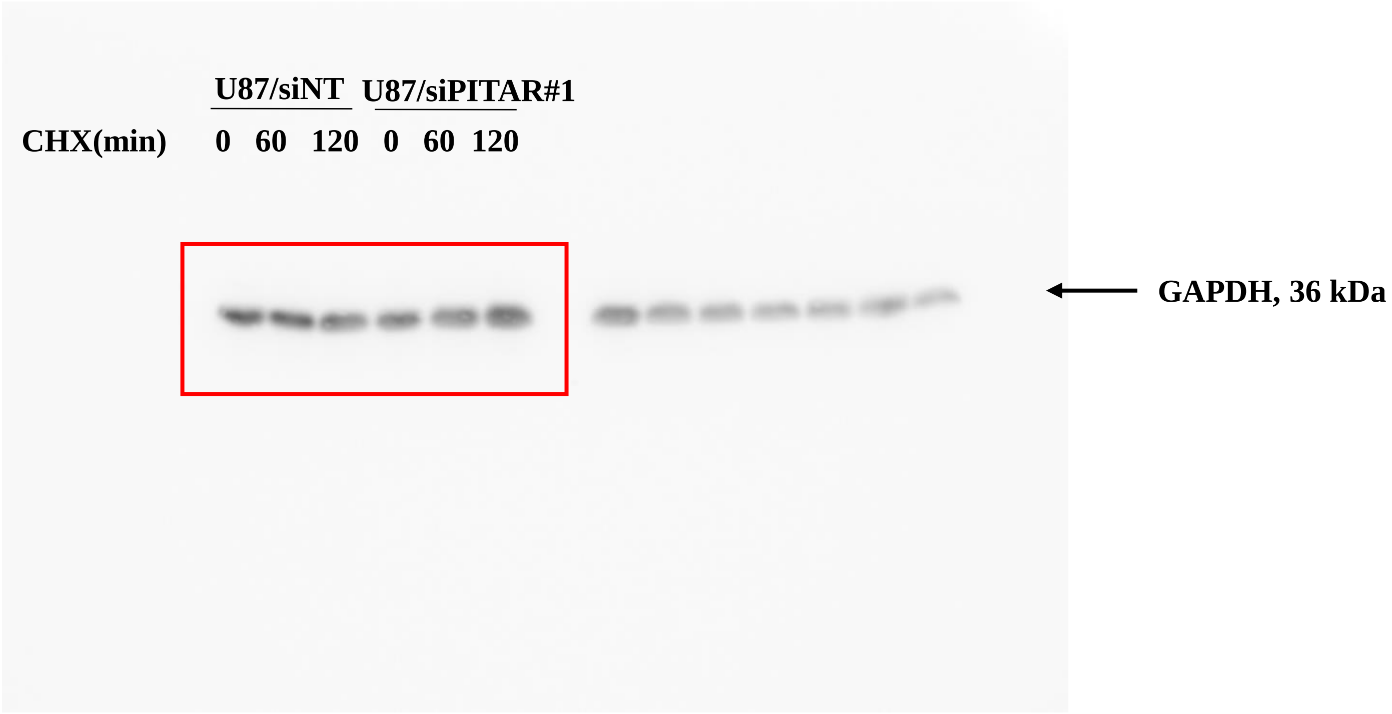

Figure 5F:

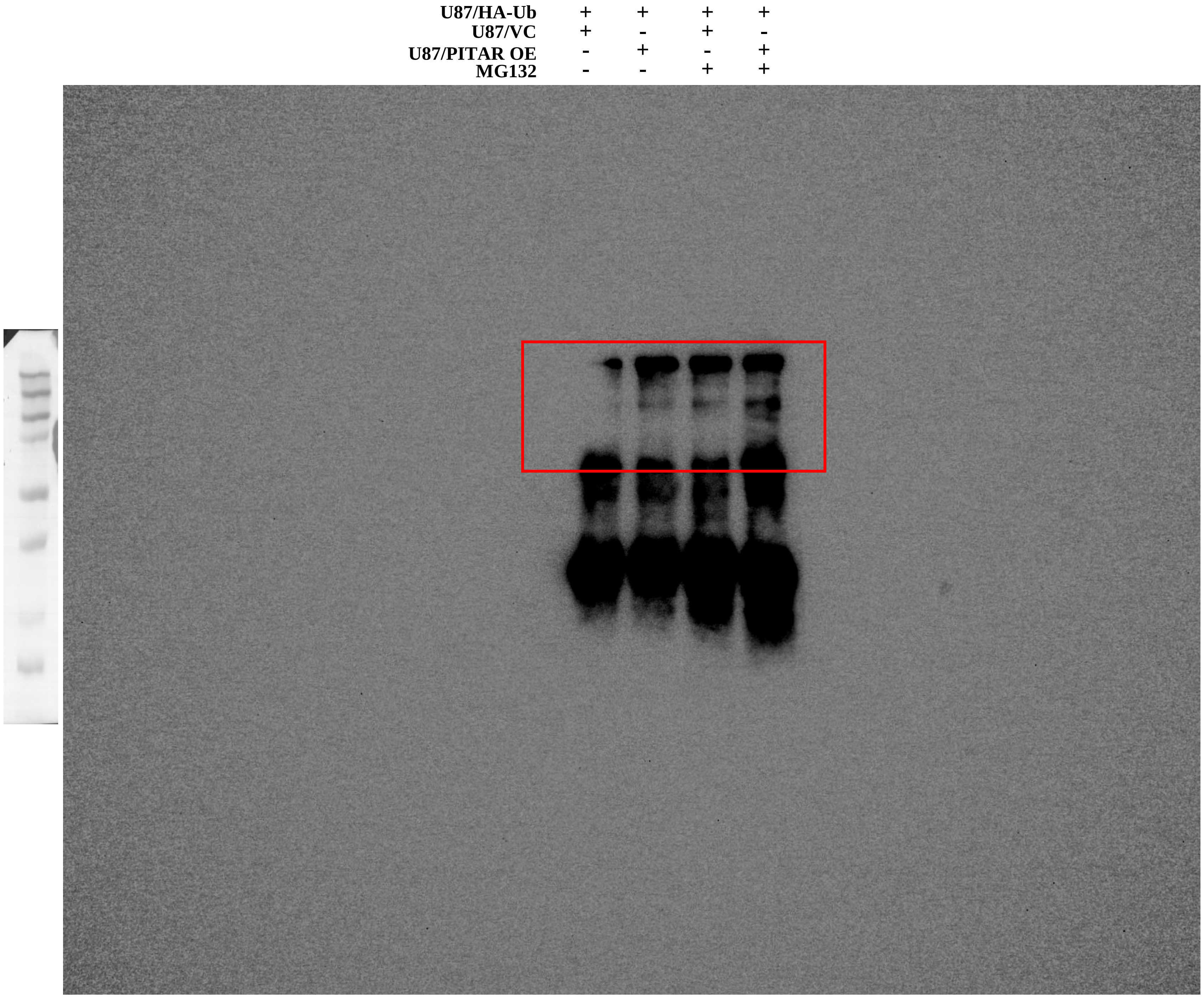

Figure 5F:

U87/HA-Ub	+	+	+	+
U87/VC	+	-	+	-
U87/PITAR OE	-	+	-	+
MG132	-	-	+	+

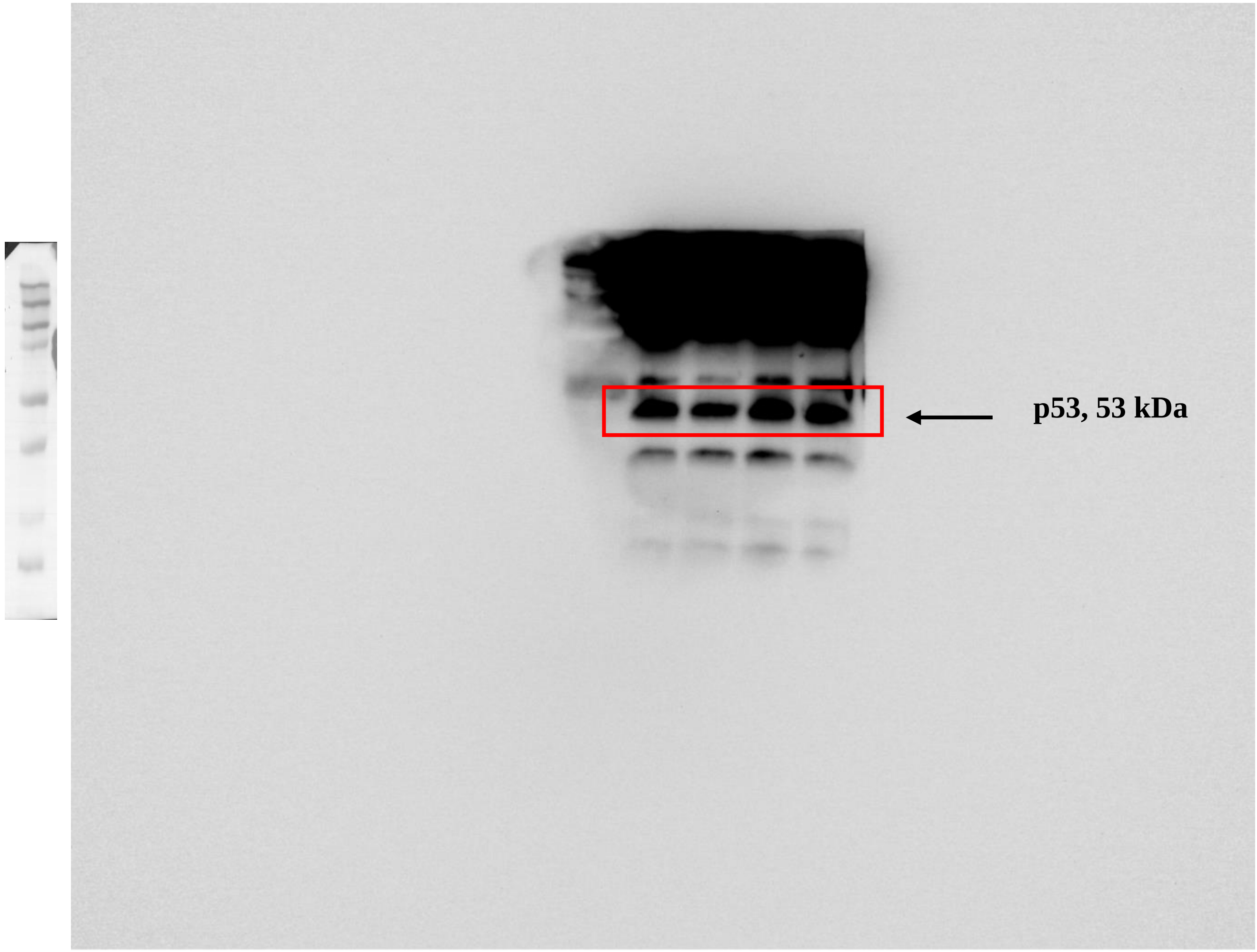

Figure 5F:

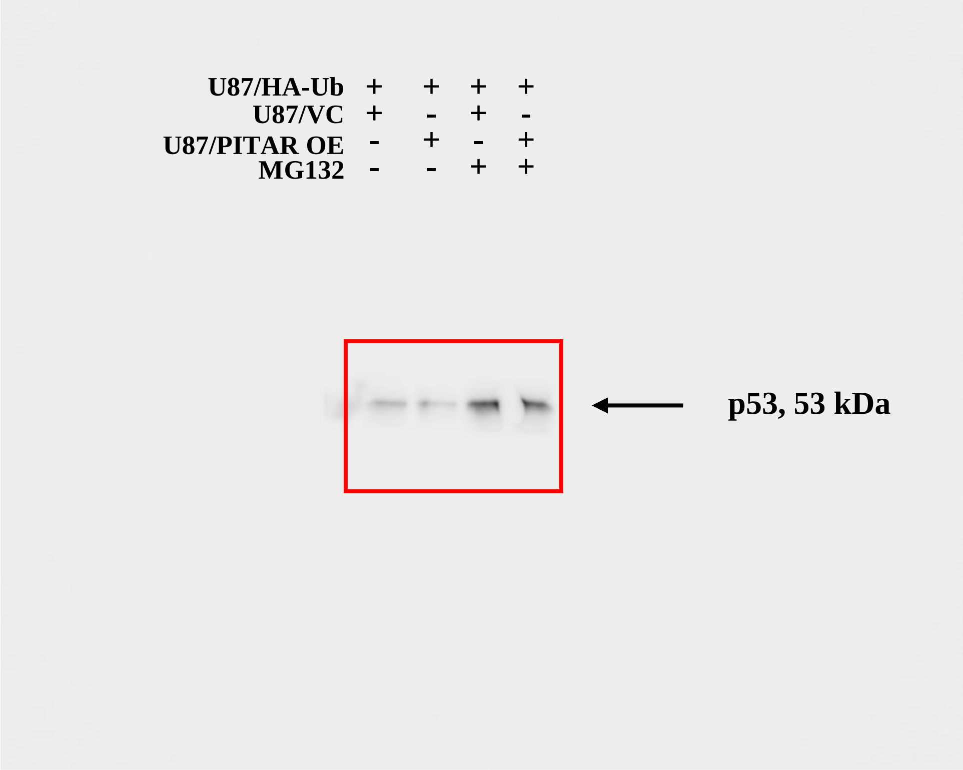
U87/HA-Ub + + + +
U87/VC + - + -
U87/PITAR OE - + - +
MG132 - - + +
p53, 53 kDa

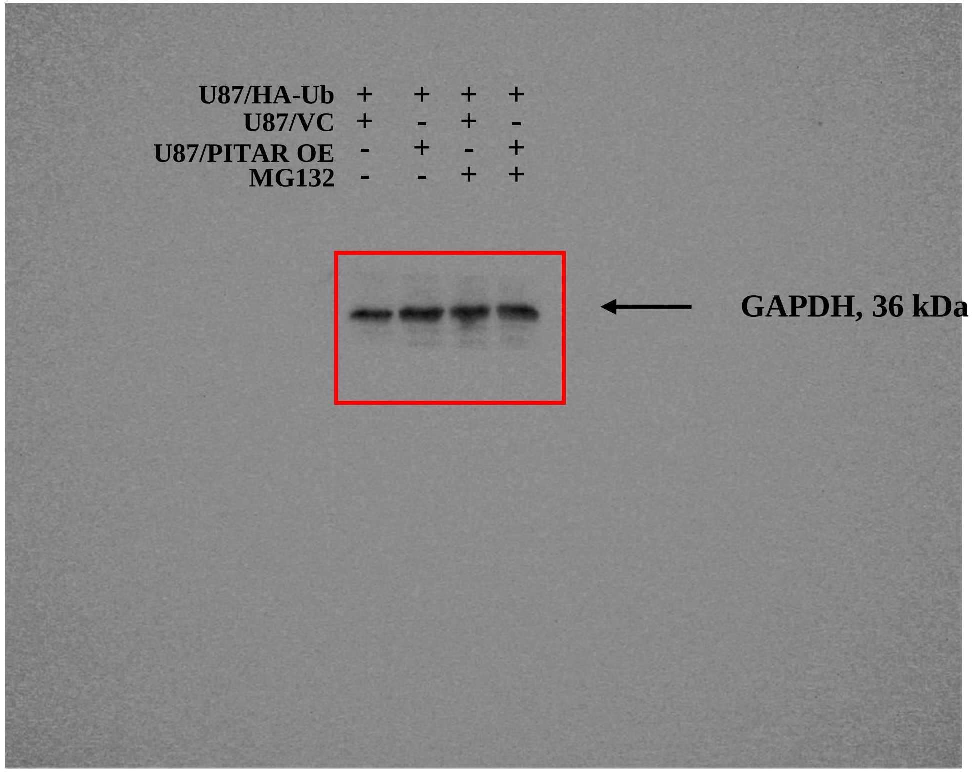
U87/HA-Ub + + + +
U87/VC + - + -
U87/PITAR OE - + - +
MG132 - - + +
GAPDH, 36 kDa

Figure 5J:

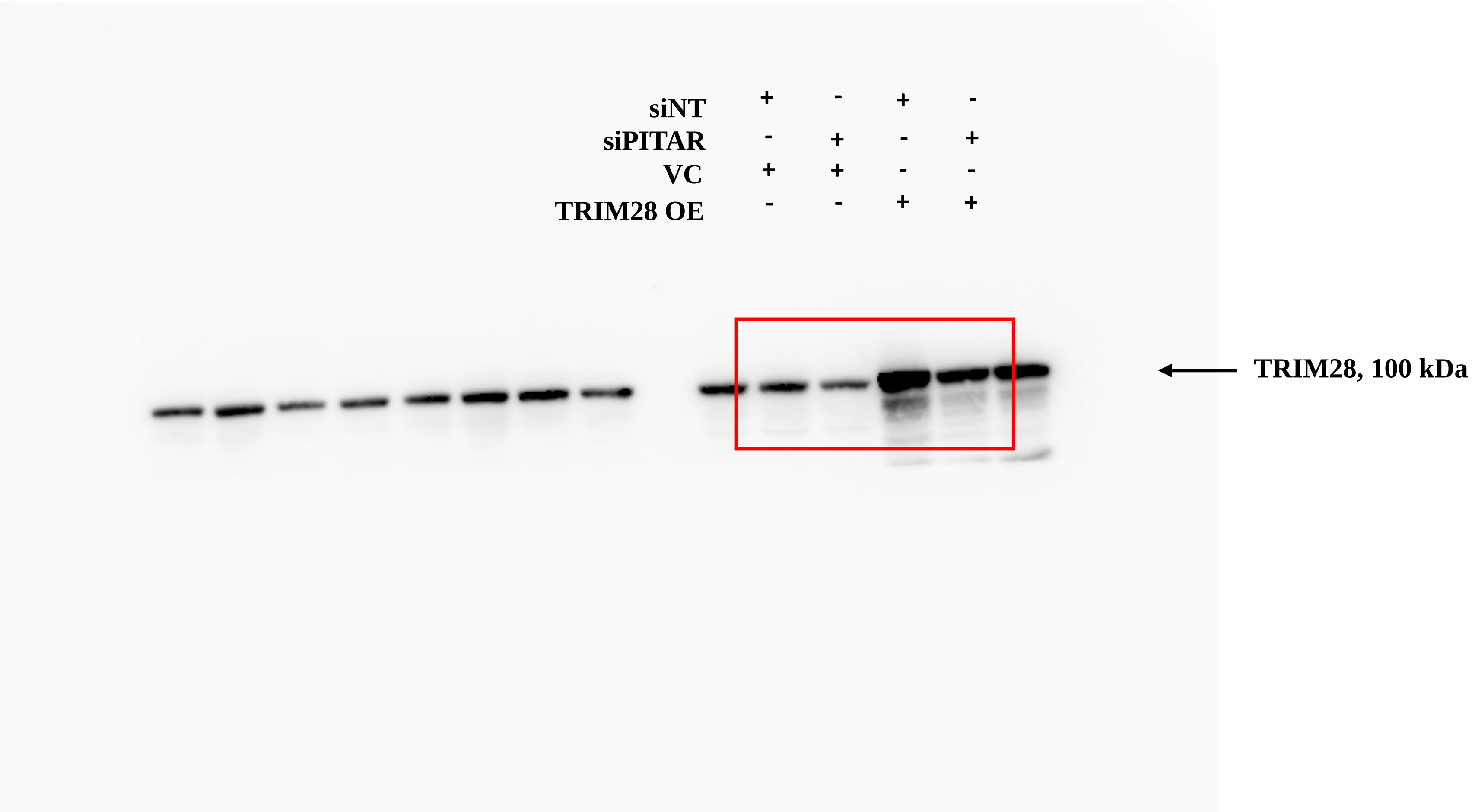

Figure 5J:

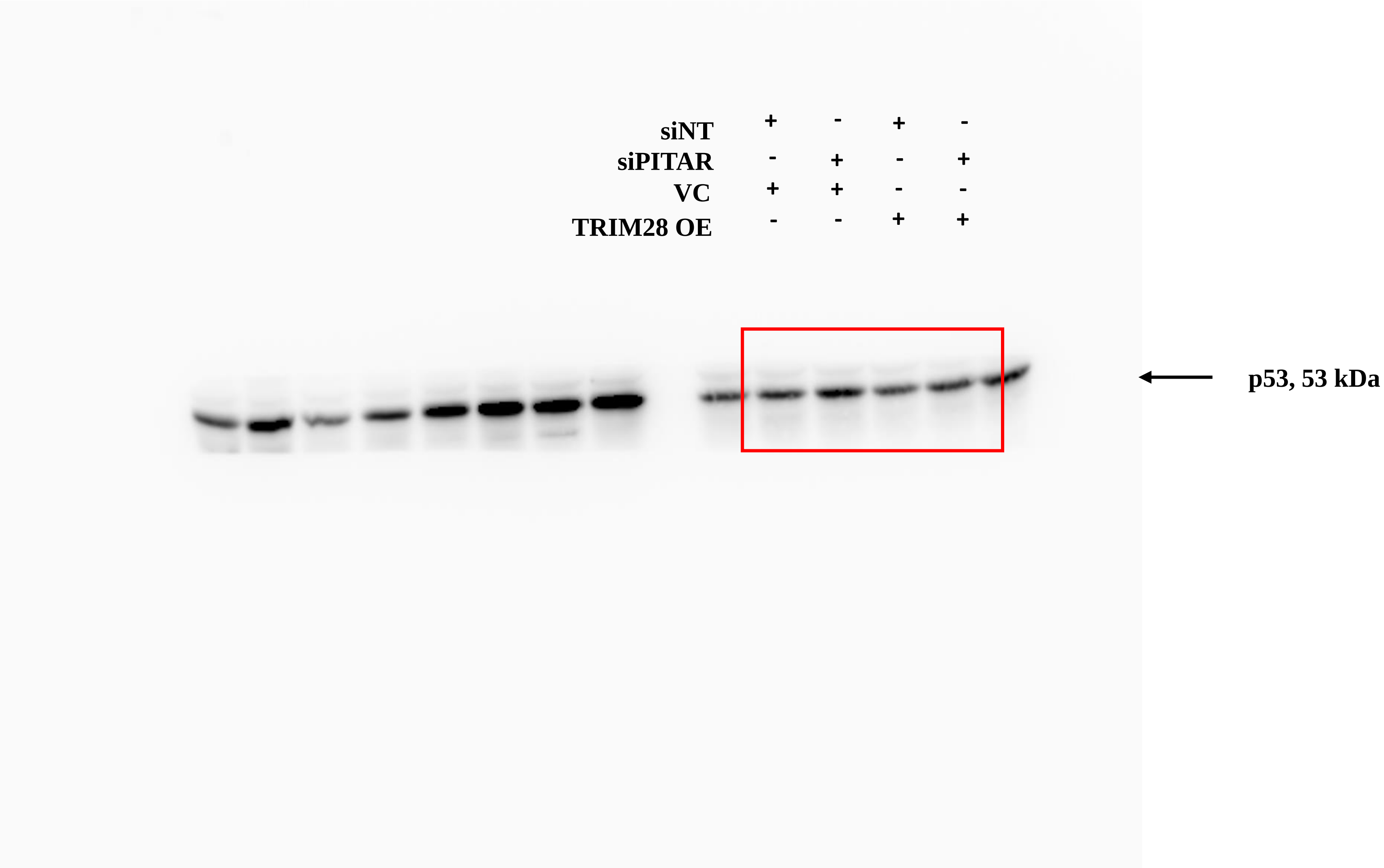

Figure 5J:

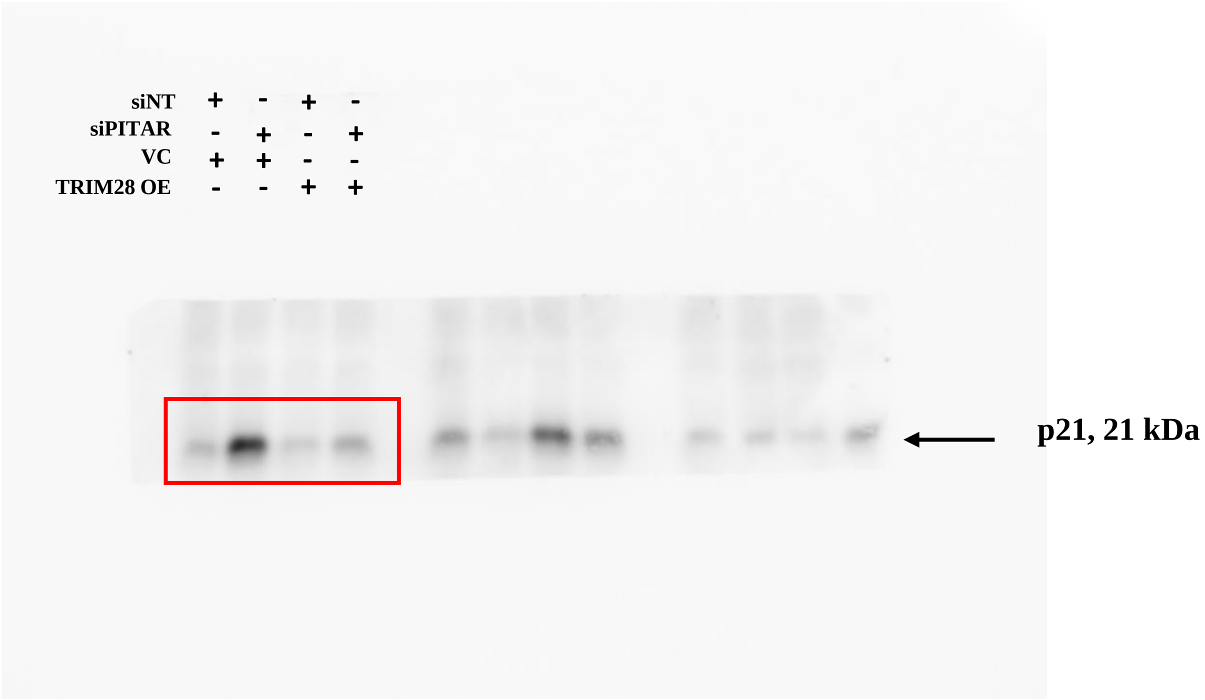

siNT + - + -
siPITAR - + - +
VC + + - -
TRIM28 OE - - + +
p21, 21 kDa

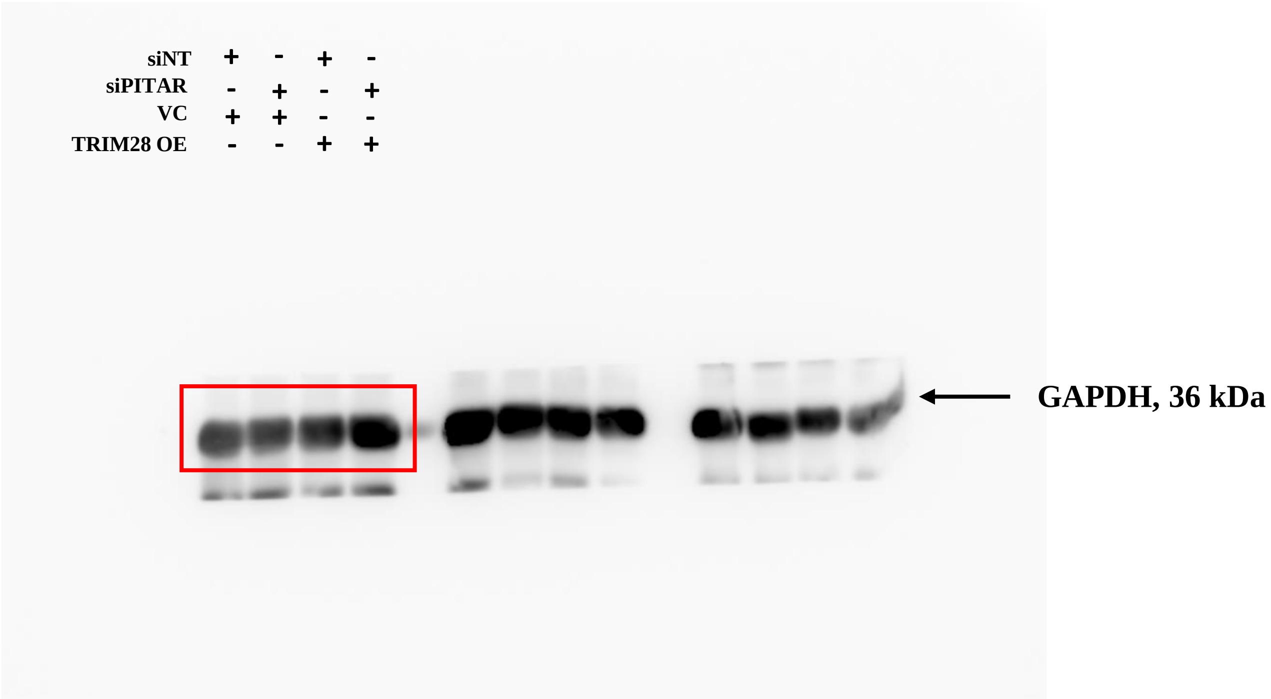

siNT + - + -
siPITAR - + - +
VC + + - -
TRIM28 OE - - + +
GAPDH, 36 kDa

Figure 5N:

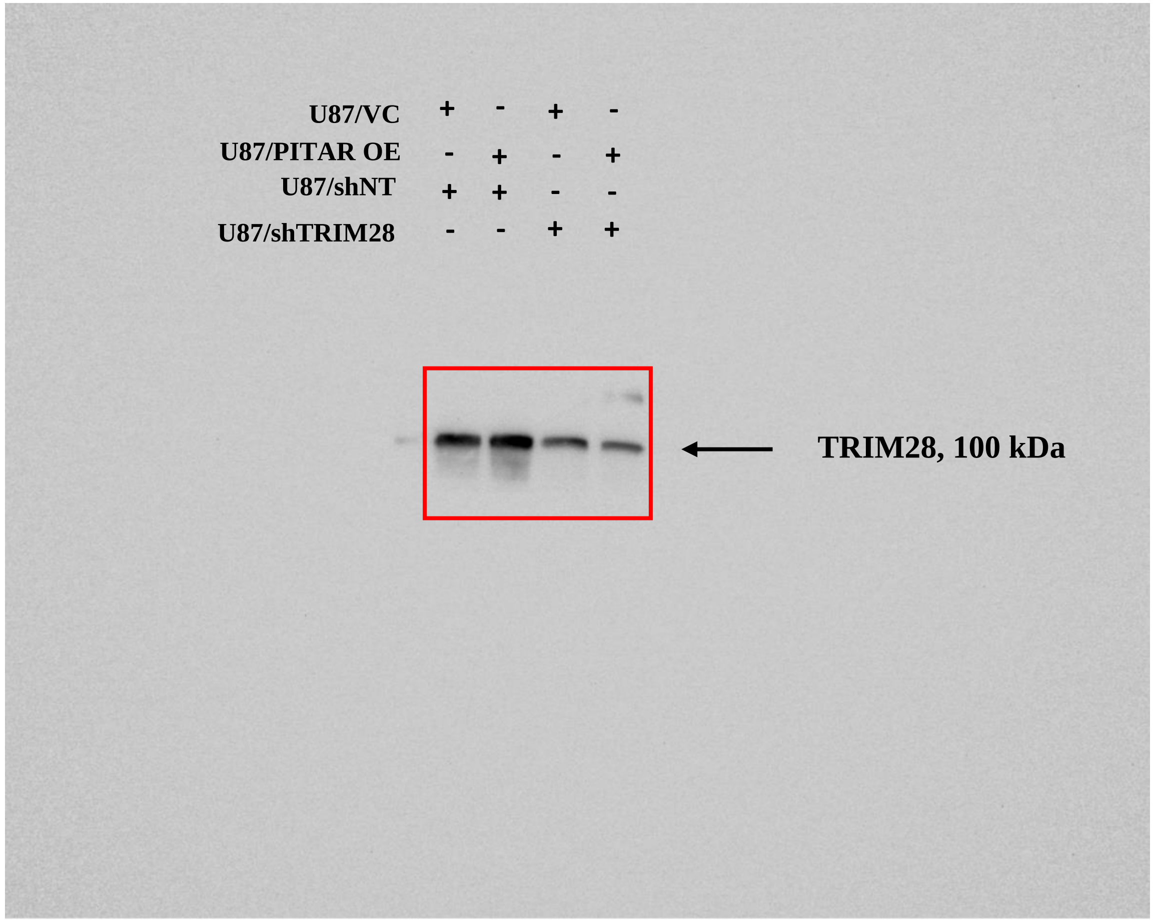

U87/VC + - + -
U87/PITAR OE - + - +
U87/shNT + + - -
U87/shTRIM28 - - + +
TRIM28, 100 kDa

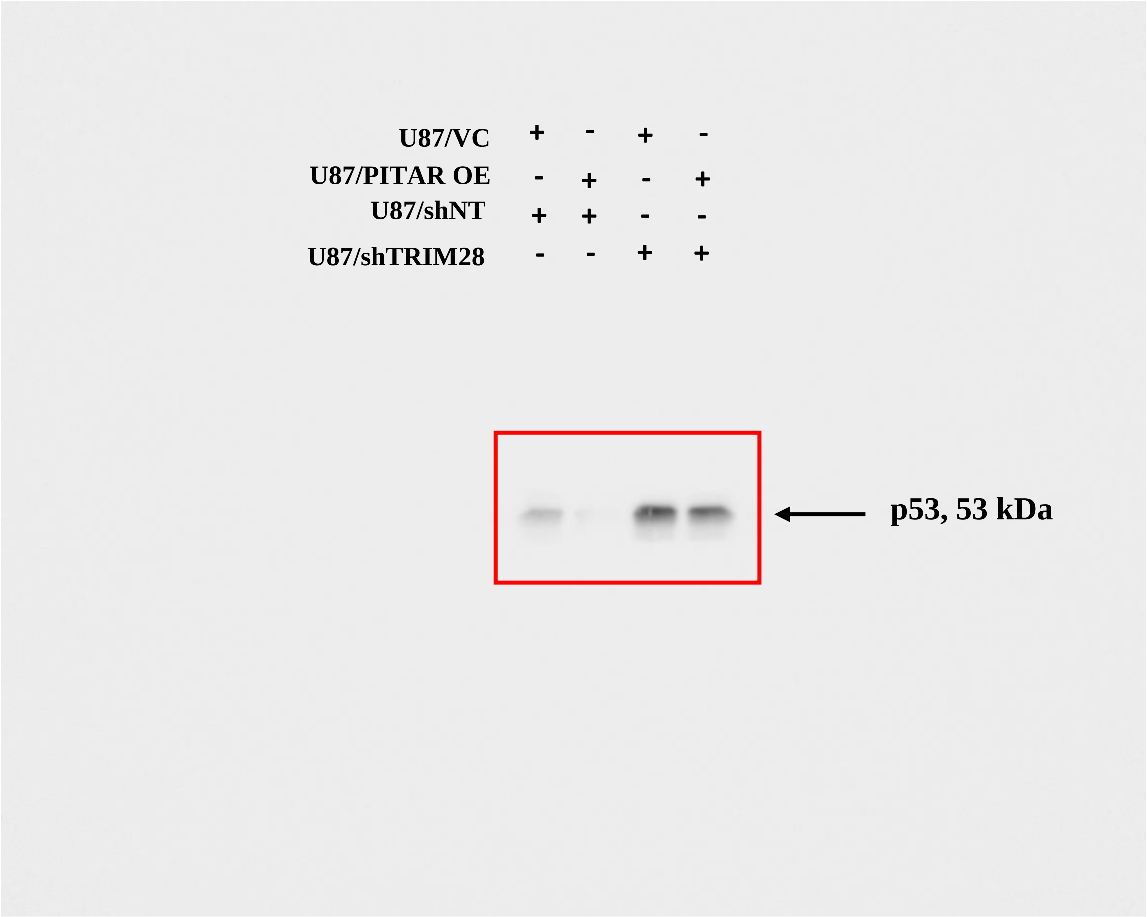

U87/VC + - + -
U87/PITAR OE - + - +
U87/shNT + + - -
U87/shTRIM28 - - + +
p53, 53 kDa

Figure 5N:

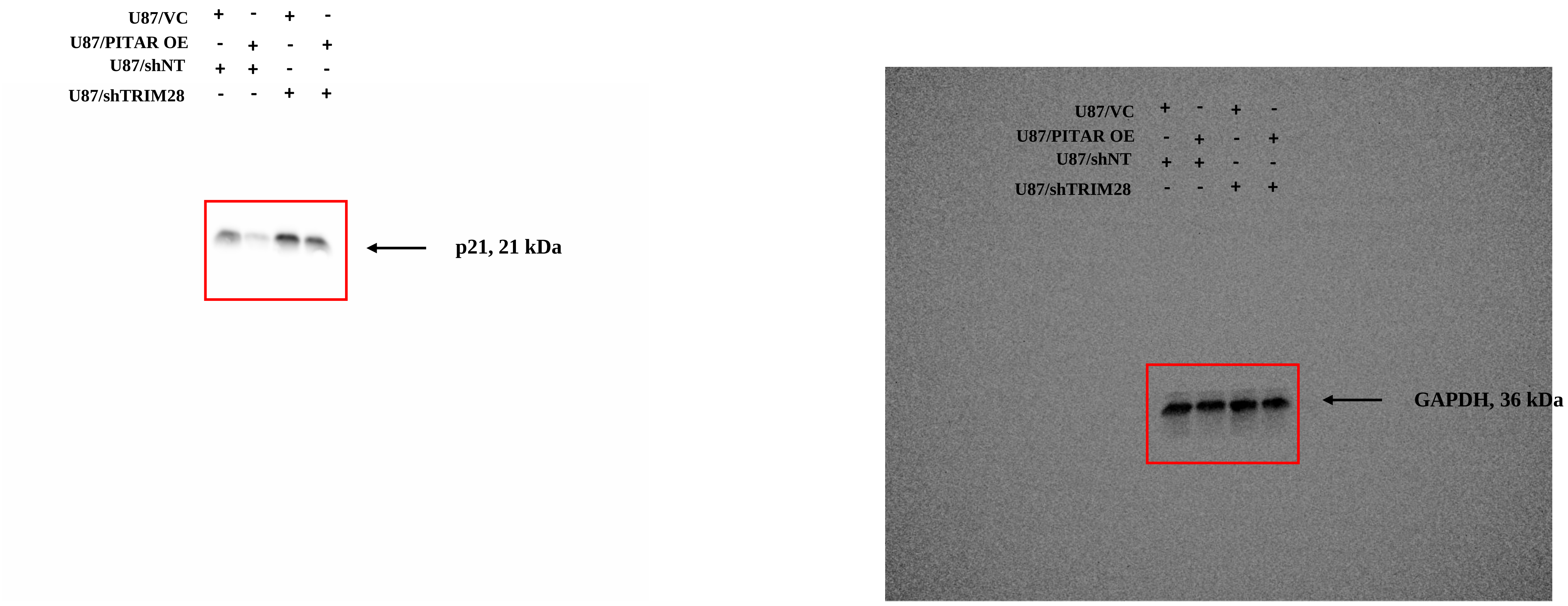
U87/VC + - + -
U87/PITAR OE - + - +
U87/shNT + + - -
U87/shTRIM28 - - + +
p21, 21 kDa
U87/VC + - + -
U87/PITAR OE - + - +
U87/shNT + + - -
U87/shTRIM28 - - + +
GAPDH, 36 kDa
