# Supplementary material for: PITAR, a DNA damage-inducible cancer/testis long noncoding RNA, inactivates p53 by binding and stabilizing TRIM28 mRNA
Source: eLife. 2024 Sep 20;12:RP88256. doi: 10.7554/eLife.88256 (PMC11415074; doi:10.7554/eLife.88256)

Figure 6-figure supplement 1C:

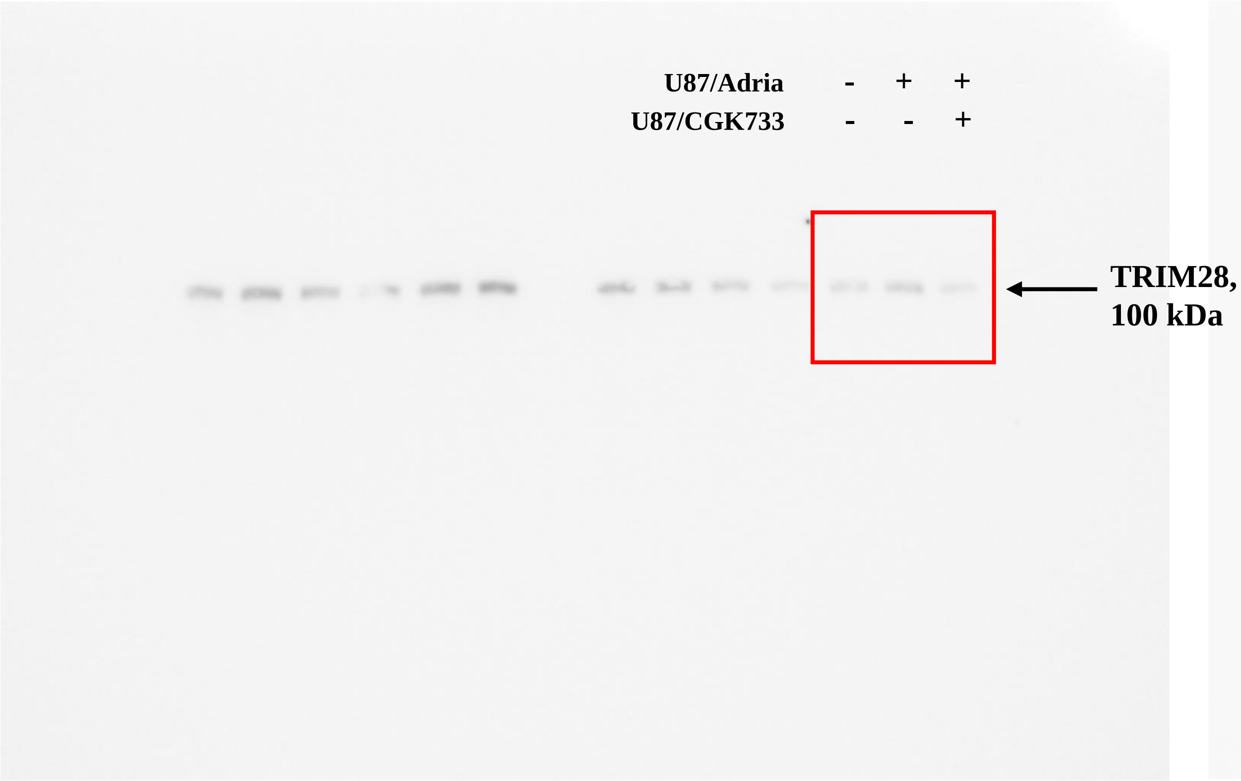

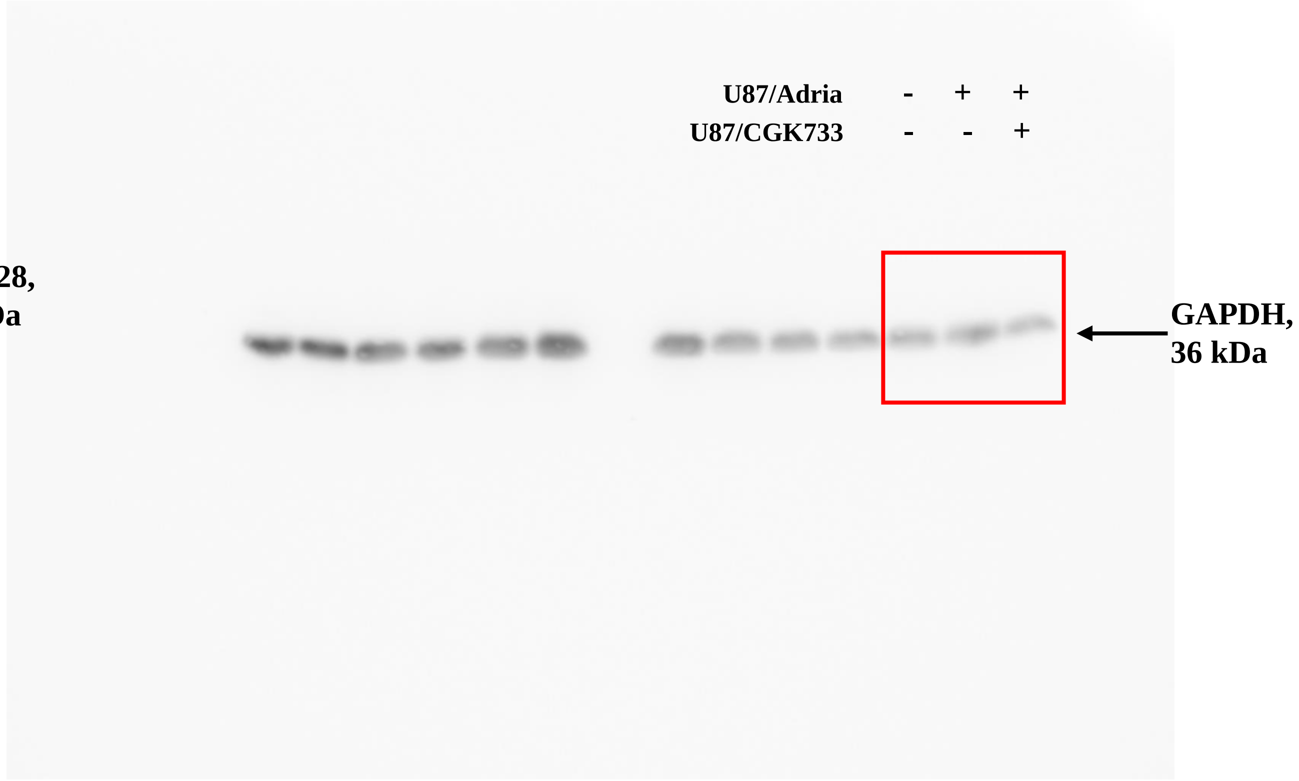

Figure 6-figure supplement 1E:

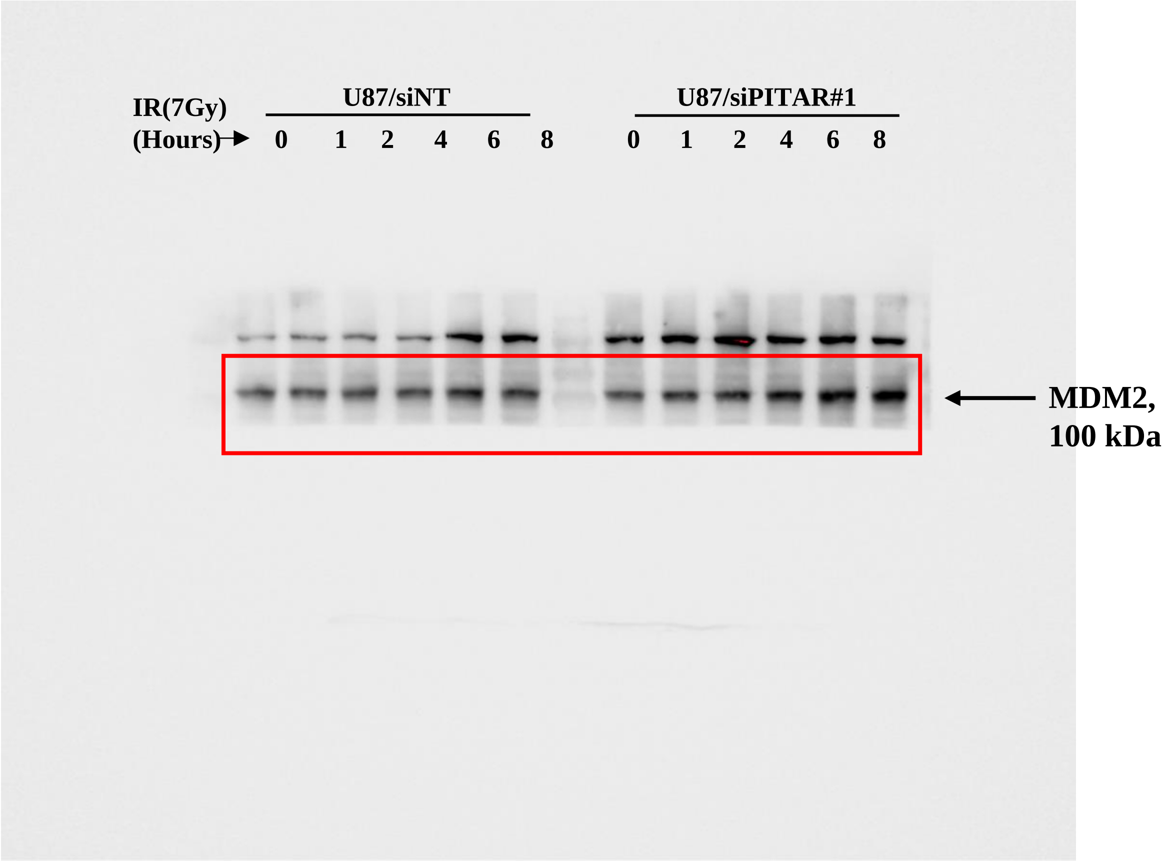

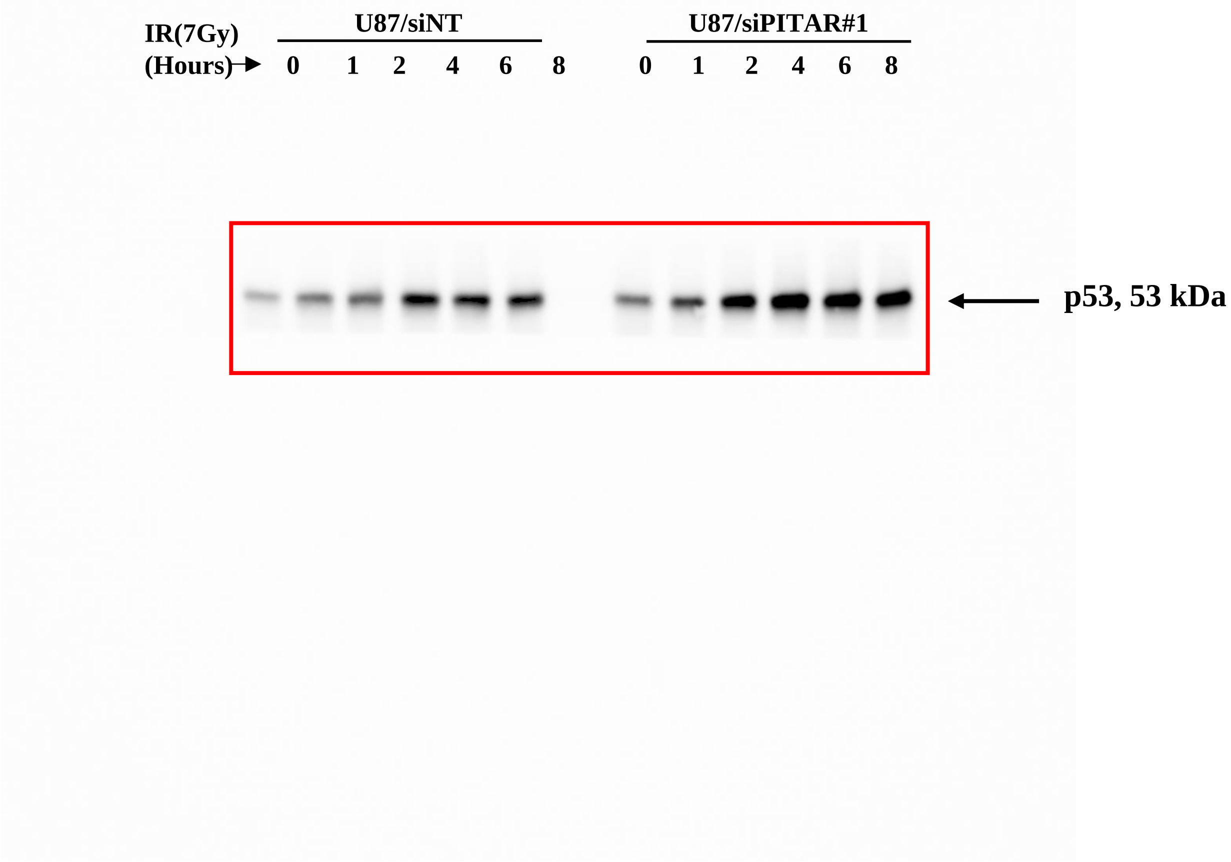

Figure 6-figure supplement 1E:

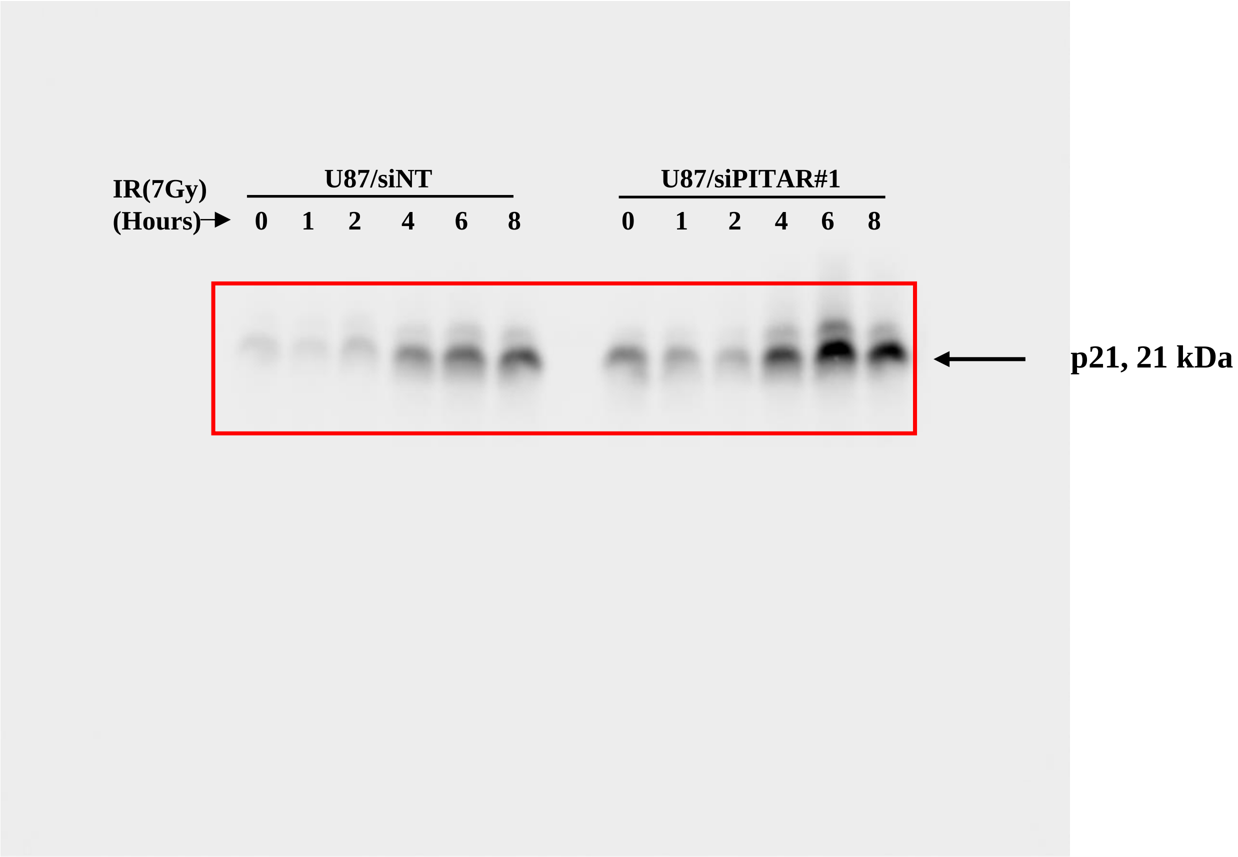

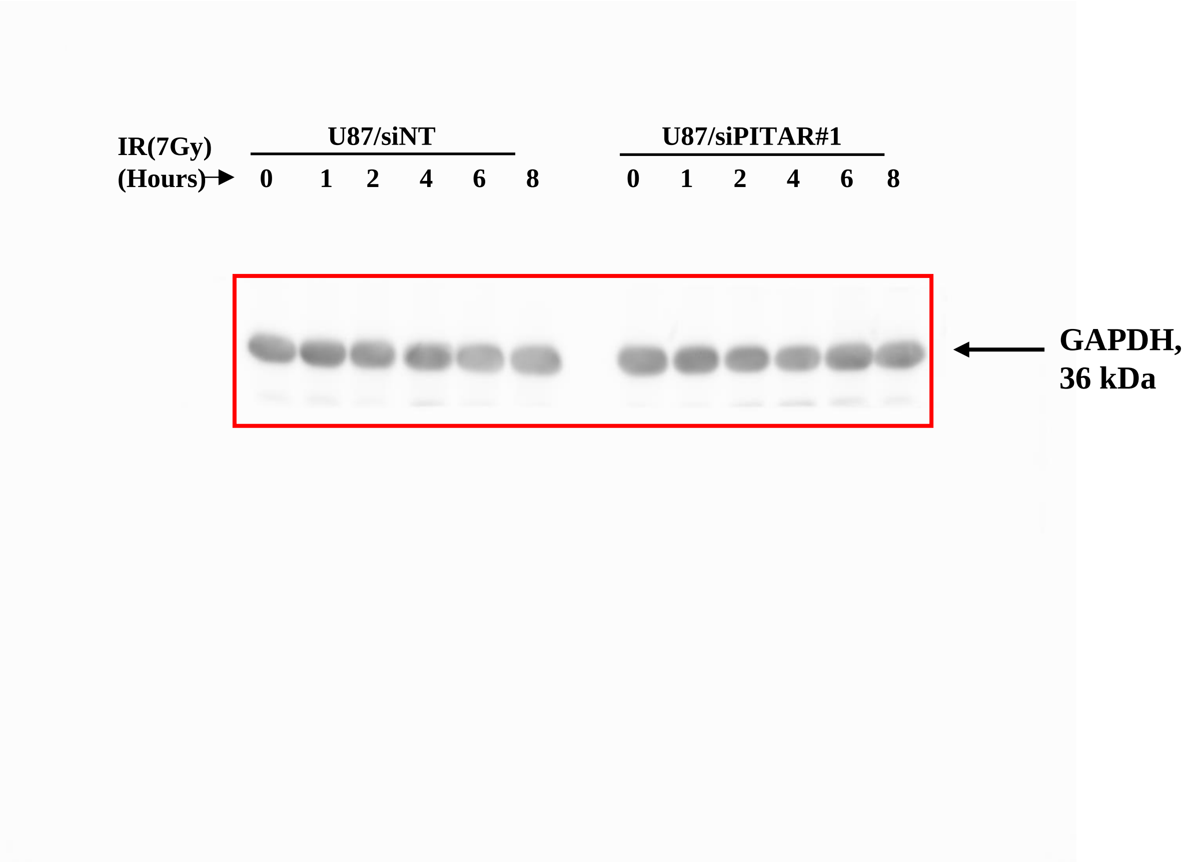

Supplement: Figure 6—figure supplement 1—source data 2. [file elife-88256-fig6-figsupp1-data2.pdf]
